# Safety and efficiency of stem cell therapy for COVID-19: a systematic review and meta-analysis

**DOI:** 10.1186/s41256-022-00251-5

**Published:** 2022-06-23

**Authors:** Minghe Zhang, Xinchun Yan, Minghui Shi, Ruihang Li, Ziwei Pi, Xiangying Ren, Yongbo Wang, Siyu Yan, Yunyun Wang, Yinghui Jin, Xinghuan Wang

**Affiliations:** 1 Center for Evidence-Based and Translational Medicine, Zhongnan Hospital of Wuhan University, Wuhan, 430071 China; 2 Department of Hepatobiliary and Pancreatic Surgery, Zhongnan Hospital of Wuhan University, Wuhan, 430071 China; 3 Department of Anesthesiology, Zhongnan Hospital of Wuhan University, Wuhan, 430071 China; 4 Department of Neurosurgery, Zhongnan Hospital of Wuhan University, Wuhan, 430071 China; 5 Department of Urology, Zhongnan Hospital of Wuhan University, Wuhan, 430071 China

**Keywords:** Stem cells, COVID-19, SARS-COV-2, Systematic review, Meta-analysis

## Abstract

**Background:**

With the COVID-19 pandemic continuing, various treatments have become widely practiced. Stem cells have a wide range of applications in the treatment of lung diseases and have therefore been experimentally used to treat patients with COVID-19, but whether the expanded use of stem cells is safe and reliable still lacks enough evidence. To address this issue, we systematically reviewed the safety and efficiency of stem cell therapy in COVID-19 cases.

**Methods:**

We searched PubMed, Embase, Web of Science, The Cochrane Library, CNKI, WanFang, VIP and SinoMed up to January 18, 2022. The included studies were assessed using the Risk-of-bias tool 1.0 and MINORS instrument. The adverse events, mortality, length of hospital day and laboratory parameters were analyzed by meta-analysis. We adhered to PRISMA reporting guideline.

**Results:**

We have included 17 studies meeting the inclusion data. There were no significant differences in AEs (OR = 0·39, 95% CI = 0·12 to 1·33, *P* = 0·13, I^2^ = 58%) and SAEs (OR = 0·21, 95% CI = 0·04 to 1·03, *P* = 0·05, I^2^ = 0%) between stem cell therapy group and control group. The analysis showed that stem cell treatment could significantly reduce the mortality rate(OR = 0·24, 95% CI = 0·13 to 0·45, *P* < 0·01, I^2^ = 0%), but was not able to cause changes in length of hospital stay or most laboratory parameters.

**Conclusions:**

The present study shows that stem cell therapy for COVID-19 has a remarkable effect on efficiency without increasing risks of adverse events and length of hospital stay. It is potentially necessary to establish the criteria for COVID-19 for stem cell therapy.

**Supplementary Information:**

The online version contains supplementary material available at 10.1186/s41256-022-00251-5.

## Introduction

In January 2020, the World Health Organization (WHO) Disease Outbreak News (DONs) confirmed the outbreak of coronavirus disease (COVID-19) caused by severe acute respiratory syndrome coronavirus 2 (SARS-CoV-2) [1]. COVID-19 has proved to be more infectious than severe acute respiratory syndrome (SARS) and Middle East respiratory syndrome (MERS), which are also caused by a coronavirus. By March 28th 2022, 480.9 million cumulative cases around the world have been reported, with 6.1 million deaths [2]. There is an urgent need to find an effective treatment in order to triumph over the pandemic.

COVID-19 has mainly caused lung injury and can also occasionally involve heart, kidney, and other organs, with manifestations varying from person to person. In the pathological progression of COVID-19, cytokine storm caused by virus induced over activation of immune cells is directly related to the occurrence of acute respiratory distress syndrome (ARDS) and contributes to the high mortality rate in severe cases [3]. Although the pathogenesis of COVID-19 has been clearly identified, there is still no specific therapy for this disease. As mentioned above, clinical studies have shown that COVID-19 patients have higher serum level of cytokines (including TNF-α, IFN-γ, IL-6) and C-reactive protein (CRP), which may account for these patients' severe symptoms and a higher case fatality rate [4]. Consequently, researchers are constantly striving to find an effective way of suppressing cytokine storm to reduce the mortality of COVID-19, so as to save patients’ lives.

A large number of clinical studies have shown that stem cell therapy has the functions of immune regulation, repair, and regeneration, which comes from stem cells’ ability of self-renewal and differentiation. Notably, among all kinds of stem cells, mesenchymal stem cells (MSCs) are the most promising type in clinical application [5]. MSCs can interact with immune cells and secret a variety of factors to inhibit excessive inflammatory reaction in COVID-19 patients, resulting in improved patient symptoms and reduced patient mortality [6]. In addition to this, several other types of stem cells are being used in pilot treatments of COVID-19 [7].

Therefore, stem cell treatment is proposed as a possible and effective way to prevent death and disability in COVID-19 patients. However, because treatment efficacy apparently varies according to dose, route of administration, and type of stem cells [8], more clinical work is needed to explore the best treatment options. In this article, we have systematically reviewed the safety and efficacy of the stem cell therapy for COVID-19.

## Methods

### Data sources and search strategy

We systematically searched PubMed, Embase, Web of Science, Cochrane Library and 4 other Chinese databases (Wanfang database, VIP database, China National Knowledge Internet (CNKI) and SinoMed database) using the keywords “SARS-CoV-2”, “COVID-19”, “Stem Cells”, and “Cell Therapy”. The complete search strategies for each database are shown in Additional file 1. All included articles were published before January 18th 2022.

### Study selection

Inclusion criteria were as follows: (1) studies should be related to stem cell therapy for COVID-19 patients with RT-PCR confirmation; (2) the outcome indicators included at least one of the following: adverse events (AEs), mortality, length of hospital stay, and laboratory parameters; (3) original research where study design was either randomized controlled trial (RCT) or non-randomized controlled trial (NRCT); (4) the treatment of the control group should be placebo or standard care. We did not restrict the type of stem cells, their dosage or mode of application. All the articles included were in English or Chinese, as only databases in these two languages were searched. The study selection follows the recommendations of the Preferred Reporting Items for Systematic Reviews and Meta-Analyses (PRISMA) [9].

We used EndNote X9.3.3 software to eliminate duplicate imported documents. Four authors (Z.M.H., Y.X.C., S.M.H, and. L.R.H.) selected the included articles according to the title and abstract. Then, the same authors screened the articles for the second time by reading the full text. Where there were disagreements between screeners, this was resolved by discussion with researchers from the Evidence-Based Medicine Center of Zhongnan Hospital of Wuhan University.

### Data extraction

Two reviewers extracted data from all eligible studies, and discrepancies were resolved by consensus. General information including author and publication year, country, number of patients, study design, cell type, administration method, control group treatment, number of transplanted cells, and frequency of cell treatment was recorded.

The primary outcome was safety based on AEs. To describe the occurrence of AEs, we extracted data from studies which reported the number of AEs and number of patients with AEs after removing duplicated samples. In one article which only mentioned no adverse reaction, AEs are considered as not reported. Only deaths that were reported explicitly as serious adverse events (SAEs) were counted in the data extraction. The treatment-related AEs were defined as adverse events that were stated explicitly in involved studies to be associated to the infusion, or at least probably to be related to the treatment.

The secondary outcome was efficiency based on mortality, length of hospital stay, and laboratory parameters. Laboratory outcomes included white blood cells (WBC) count, neutrophiles count, lymphocytes count, platelets (PLT) count, CRP level, IL-6 level, tumor necrosis factor α (TNF-α) level, D-dimer level, fibrinogen level, and ferritin level. To account for the different times of measurement of laboratory parameters, we set two time periods when combining the data on laboratory indicators: 0–4 days and 5–8 days, in order to conduct a meta-analysis on the data of a particular parameter within the same time period.

### Quality assessment

According to the type of research article, we included 17 clinical controlled studies. The risk of bias of ten RCT articles was assessed by using the criteria of the Cochrane back review group, Risk-of-bias tool 1·0. This tool evaluates the quality of articles using seven aspects: random sequence generation, allocation concealment, blinding of participants and personnel, blinding of outcome assessment, incomplete outcome data, selective reporting, and other biases. The evaluation results are high risk, unclear and low risk. While the risk of bias of seven NRCT articles was assessed by using the Methodological index for non-randomized studies (MINORS) instrument [10], which consists of twelve items. With 0 ~ 2 points for each item, 0 for not reported, 1 for reported without sufficient information and 2 for reported with adequate information, giving a total score of 24 points.

### Statistical analysis

We only included literatures describing the original data to extract data. Standardized mean difference (SMD) and relative risk (RR) with corresponding 95% confidence intervals (CIs) were reported for continuous and binary variables, respectively. For continuous variables, if the authors only reported medians, ranges and/or interquartile ranges, we used a web tool to calculate the sample mean and standard deviation [11, 12]. To assess the heterogeneity across each study, I^2^ statistics was used. I^2^ > 50% was considered to have significant heterogeneity. Publication bias was tested by using Egger’s test and Begg’s test [13, 14]. We only detected publication bias of studies included in the mortality meta-analysis, and the studies included in the meta-analysis of other outcomes were not assessed due to small numbers.

Fixed effect model (FEM) was used when we combined data from different time points within the same time period (e·g·,0–4 day or 5–8 day) from the same study, and random effect model (REM) was used when we combined data from different studies within the same time period. To discern whether our conclusions were influenced by different study types (RCTs and NRCTs), we performed subgroup analyses of all outcomes that included more than 3 pieces of literature. All statistical analysis was performed using the package "meta"(version 5·2·0) of R 4·1·2 software. For binary variables of sparse data, hypergeometric normal model (HNM) was used to synthesize using the “metabin” function. Continuous variables were synthesized through the “metacount” function.

## Results

### Study characteristics

**Fig. 1:**
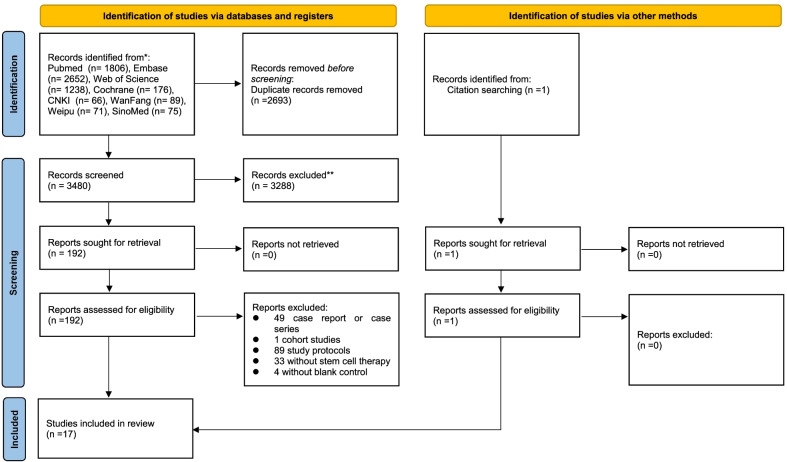
PRISMA flow diagram. Summary of evidence search and study slection

The initial literature search identified a total of 6174 potentially relevant records. After removing 2693 duplicates, 3481 articles were screened by titles and abstracts, and 3288 articles were excluded. 193 studies were reviewed using the full texts and finally 17 articles met the inclusion criteria and were included in the systematic review and meta-analysis (Fig. 1). Excluded studies and the reason are shown in Additional file 2. All included studies were clinical trials, of which ten [15–24] were RCTs while other seven were NRCTs [25–31]. Four studies [16, 17, 23, 24] were from the same clinical trial, and in addition to safety and efficacy of stem cell therapy, they also reported other characteristics such as radiological changes. Two studies [18, 19] by Shu et al. reported on the same trial, the first giving the outcome after 28 days and the second being the follow-up report after one year. The specific design features of the included studies are presented in Table 1.

**Table 1:** Clinical and study characteristics

Article	Country	Number of patients	Patients condition	Study design	Cell type	Administration	Control group treatment	Number of transplanted cells	Frequency of cell treatment
Lanzoni et al. [15]	America	24	mild-to-moderate or moderate-to-severe	Double-blind, phase 1/2a, RCT	UC-MSCs	IV	placebo	100 ± 20 × 10^6 (a)	2 doses
Ventura-Carmenate et al. [16]	United Arab Emirates	139	moderate, severe or critically severe	Openlabel, phase 1/2, RCT	PBNHESCC	Nebulization	standard care	2 × 10^6 (c)	2 doses
Torres Zambrano et al. [17]	United Arab Emirates	139	moderate, severe or critically severe	openlabel, phase 1/2, RCT	PBNHESCC	Nebulization	standard care	2 × 10^6 (c)	2 doses
Shi et al. [18]	China	100	severe	Double-blind, phase 2, RCT	UC-MSCs	IV	placebo	4.0 × 10^7 (a)	3 doses
Shi et al. [19]	China	100	severe	double-blind, phase 2, RCT	UC-MSCs	IV	placebo	4.0 × 10^7 (a)	3 doses
Zhu et al. [20]	China	58	severe	single-blind, phase 2, RCT	UC-MSCs	IV	placebo	1 × 10^6 (b)	1 dose
Adas et al. [21]	Turkey	30	severe	prospective, 3-parallel armed, RCT	UC-MSCs	IV	conventional treatment	3 × 10^6 (b)	3 doses
Dilogo et al. [22]	Indonesia	40	critically severe	double-blind, multicentered, RCT	UC-MSCs	IV	standard care	1×10^6 (b)	1 dose
Torres Zambrano et al. [23]	United Arab Emirates	44	critically severe	openlabel, phase 1/2, RCT	PBNHESCC	Nebulization	standard care	2 × 10^6 (c)	2 doses
Torres Zambrano et al. [24]	United Arab Emirates	139	moderate, severe or critically severe	openlabel, phase 1/2, RCT	PBNHESCC	Nebulization	standard care	2 × 10^6 (c)	2 doses
Wei et al. [25]	China	25	moderate, severe or critically severe	pilot trial, NRCT	hUC-MSCs	IV	standard care	1 ×10^6 (b)	NR
Xu et al. [26]	China	44	severe or critically severe	multicenter, open-label, phase 1, NRCT	MenSCs	IV	standard care	3 ×10^7 (a)	3 doses
Meng et al. [27]	China	18	moderate or severe	phase 1, NRCT	UC-MSCs	IV	standard care	3 × 10^7 (a)	3 doses
Häberle et al. [28]	Germany	23	severe	single center, open-label, NRCT	hBM-MSCs	IV	standard care	1×10^6 (b)	2 or 3 doses
O.Ercelen et al. [29]	Turkey	11	severe or critically severe	open-label, phase I, NRCT	MSCs	IV	placebo	1×10^6 (b)	1 dose
Leng et al. [30]	China	10	moderate, severe or critically severe	pilot trial, NRCT	MSCs	IV	placebo	1 × 10^6 (b)	1 dose
Shu et al. [31]	China	41	severe	open-label, pilot study, IRGT	hUC-MSCs	IV	standard care	2 × 10^6 (b)	NR

*RCT* randomized control trials, *IRGT* individually randomized group treatment, *NR* no report; a, cells per infusion, *b* per kg, *c* total dose, *IV* intravenous, *MSCs* mesenchymal stem cells, *UC-MSCs* umbilical cord mesenchymal stem cells, *hUC-MSCs* human umbilical cord mesenchymal stem cells, *PB‑NHESC‑C* peripheral blood non‑hematopoietic enriched stem cell cocktail, *hBM-MSC* human bone marrow mesenchymal stem cells, *MenSCs* menstrual blood-derived mesenchymal stem cells

### Study quality assessment

For RCT studies, two of them [20, 21] lacked comprehensive information on random sequences in the original text, and one study [16] had obvious missing data. The study by Zhu, R et al. [20] adopted a single-blind method, and the study by Yendry Ventura‑Carmenate et al. [16] is an open-label trial, therefore, the item of blinding of participants and personnel were rated as high risk for these studies. For NRCT studies, all seven of them had control groups. The mean score was 17·86 (range 13–20) out of a total of 24 points. None of the studies counted sample sizes. Additional file 3. and Additional file 4. respectively show authors' judgments about each risk of bias item for RCT and scores of MINORS Scale for NRCT.

### Patient characteristics

17 clinical studies were conducted in 6 countries with the total number of patients by country as follows: China (n = 296), United Arab Emirates (n = 139), Turkey (n = 41), Indonesia (n = 40), USA (n = 24), Germany (n = 23). The specific inclusion and exclusion criteria for patients in each study are shown in Additional file 5.

### Intervention characteristics

In the 17 articles included, four types of cell therapies were used: (1) umbilical cord mesenchymal stem cells (UC-MSCs) [15, 18–22, 25, 27, 31]; (2) human bone marrow mesenchymal stem cells (BM-MSCs) [28]; (3) menstrual blood-derived mesenchymal stem cells (MenSCs) [26]; (4) peripheral blood non‑hematopoietic enriched stem cell cocktail (PB‑NHESC‑C) [16, 17, 23, 24]. Two other studies used mesenchymal stem cells but did not mention the source [29, 30]. Except for PB‑NHESC‑C administered by nebulization, other cells were administered by intravenous injection. The specific information of therapy dose and frequency can be found in Table 1.

### Primary outcome: Safety

#### Adverse Events (AEs)

In the 17 studies involved, six studies mentioned AEs in both experimental and control groups [15, 16, 18–20, 26], while five studies mentioned AE in the experimental group only [22, 25, 27, 29, 30]. The other six studies did not mention AEs [17, 21, 23, 24, 28, 31]. Detailed information can be found in Table 2. It is worth mentioning that two studies reported all AEs from grade 1 to grade 4 according to Common Terminology Criteria for Adverse Events (CTCAE) version 5.0 [15, 16] while another two studies reported AEs from grade 1 to grade 4 without stating whether CTCAE was referenced [18, 19, 26]. Other studies that mentioned AEs did not describe the specific criteria for AEs or SAEs [20, 22, 25, 27, 29, 30]. There were two studies which mentioned that no adverse effects were observed in the treatment group, and did not describe any adverse events, in these studies AEs were considered as not reported [23, 29].

**Table 2:** Numbers of AEs and patients with AEs

Article	Numbers of patients with AEs, n(%), Experimental; Ctrl	Numbers of AEs, n, experimental; Ctrl	Numbers of patients with SAEs, n(%), experimental; Ctrl	Numbers of SAEs, n, experimental; Ctrl	Number of AEs related to teatment, n
Lanzoni et al. [15]	8(66.7) ; 11(91.67)	46 ; 53	2(16.7) ; 8(66.7)	6 ; 16	1*
Ventura-Carmenate et al. [16]**	50(72.5) ; 51(72.9)	107 ; 133	NR ; NR	NR ; NR	0
Torres Zambrano et al. [17]**	NR ; NR	NR ; NR	NR ; NR	NR ; NR	NR
Shi et al. [18]***	37(56.9) ; 21(60)	72 ; 36	1(1.5) ; 0	1 ; NR	0
Shi et al. [19]***	54(83.1) ; 26(74.3)	106 ; 54	1(1.5) ; 0	1 ; NR	0
Zhu et al. [20]	3(10.3) ; 13(44.8)	20 ; 34	0 ; 0	0 ; 0	0
Adas et al. [21]	NR ; NR	NR ; NR	NR ; NR	NR ; NR	NR
Dilogo et al. [22]	0 ; NR	0 ; NR	0 ; NR	0 ; NR	0
Torres Zambrano et al. [23]**	NR ; NR	NR ; NR	NR ; NR	NR ; NR	NR
Torres Zambrano et al. [24]**	NR ; NR	NR ; NR	NR ; NR	NR ; NR	NR
Wei et al. [25]	1(8.3) ; NR	3 ; NR	1(8.3) ; NR	3 ; NR	0
Xu et al. [26]	20(76.9) ; 18(100)	56 ; 59	NR ; NR	10 ; 15	0
Meng et al. [27]	3(33.3) ; NR	4 ; NR	0 ; NR	0 ; NR	3*
Häberle et al. [28]	NR ; NR	NR ; NR	NR ; NR	NR ; NR	NR
O.Ercelen et al. [29]	NR ; NR	NR; NR	0 ; NR	0 ; NR	0
Leng et al. [30]	NR ; NR	NR ; NR	0 ; NR	0 : NR	0
Shu et al. [31]	NR ; NR	NR ; NR	NR ; NR	NR ; NR	0

*AEs* adverse events, *SAEs* serious adverse events

*The former study [15] mentioned three “infusion associated events” during the first round of infusion. The latter study [27] reported number of AEs in relationship to treatment and only one AE was considered “Probable”, which is the highest degree in reported relationship.

**These four studies are based on one clinical trial. The first study [16] focused on the safety and efficacy of stem cell therapy. The second study emphasized the radiographic outcome [17]. The third study [23] focused only on the renal involvement of the critically ill patients in the trial, and the last study [24] explored secondary sepsis and urinary tract infections in patients.

***These two studies are based on the same patients. The latter [19] is a one-year follow-up of the former and contains all AEs of the former [18].

There were 308 and 315 AEs reported in experimental and control groups respectively. In studies which mentioned AEs in both groups, AEs were reported in 61·69% (124/201) of patients in the experimental group, while there were 74·39% (122/164) in the control. We screened AEs with a frequency of more than 10, and the number of patients with elevated ALT and LDH in stem cell treatment group was more than that in control group, while the occurrence of ARDS, sepsis, and multiple organ failure was less than that in control group (Table 3). The remaining AEs reported in all studies are shown in Additional file 6.

**Table 3:** Most frequent adverse events

AEs*	Experimental	Control	Total	Articles
Increased respiratory rate	30	34	64	[16]
Increased blood pressure	20	20	40	[16, 20, 26]
Fever	17	22	39	[15, 16, 20, 26]
Elevated ALT	19	15	34	[18, 20]
Anemia	15	17	32	[15, 16, 26]
Sepsis	6	15	21	[15, 16]
Deaths with no cause reported	6	14	20	[15, 16]
Decreased absolute lymphocyte	8	9	17	[16]
Elevated LDH	10	7	17	[18, 26]
Disease progression	5	9	14	[16]
Hypokalemia	9	3	12	[15, 18, 26]
Bacterial infections	9	3	12	[15, 18, 25]
Metabolic alkalosis	5	6	11	[16, 18]
Acute Respiratory Distress Syndrome	4	7	11	[16, 26]
Multiple organ failure	3	8	11	[16, 26]

^*^AEs occurring a total of more than 10 times in all studies

**Fig. 2:**
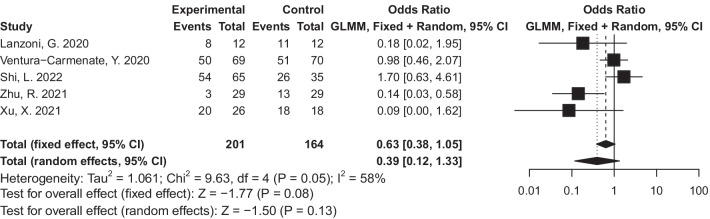
Forest plot of adverse events (AEs): odds ratio (95% CI) and pooled estimates

A meta-analysis has been performed to compare the number of adverse events between the stem cell group and the control group and there was no significant difference (OR = 0·39, 95% CI = 0·12 to 1·33, *P* = 0·13, I^2^ = 58%). (Fig. 2).

#### Serious adverse events (SAEs)

In the 17 studies involved, five studies reported the occurrence of serious adverse events [15, 16, 18, 25, 26]. There were 18 and 31 SAEs reported in the experimental and control groups respectively, and none of the SAEs were MSC treatment-related according to the authors. In studies which mentioned SAEs in both groups (no matter whether any SAE occurred), SAEs occurred in 4.94% (3/106) of patients in the experimental group, and 10.53% (8/76) in the control (Table 2). All the SAEs which occurred are listed in Additional file 7.

Shi, L et al. only reported a pneumothorax (CTCAE grade 3) in the stem cell group, and the patient recovered after conservative treatment [18]. Wei, F et al. reported a death due to respiratory failure, circulatory failure, and secondary infection, which was judged to be unrelated to MSC infusion [25]. Lanzoni, G et al. reported 2 and 16 SAEs while Ventura.et al. reported 35 and 57 SAEs in experimental and control groups respectively, without specific descriptions [15, 21].

**Fig. 3:**
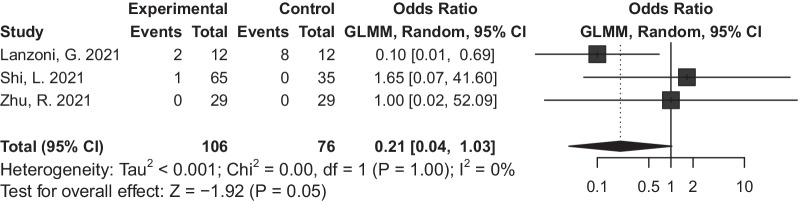
Forest plot of severe adverse events (SAEs): odds ratio (95% CI) and pooled estimates

In studies mentioning the number of patients with SAE, we also compare the number of patients having SAEs between experimental and control group and there was no significant difference. (OR = 0·21, 95% CI = 0·04 to 1·03, *P* = 0·05, I^2^ = 0%) (Fig. 3).

#### AEs Related to stem cell treatment

In the included studies, four infusion-related AEs were reported in two studies [15, 16]. A patient with bradycardia experienced a worsening of bradycardia and required brief vasopressors treatment [15]. Two patients in the experimental group experienced transient facial flushing and fever immediately on infusion, which resolved within 4 h [27]. The study also reported a serious hypoxemia within 12 h of infusion, which is considered to be associated with the progression of COVID-19, and the patient recovered after humidified high-flow nasal catheter oxygen therapy [27].

There were no other treatment-related AEs reported in the remaining studies.

### Secondary outcome: efficiency

#### Mortality

**Fig. 4:**
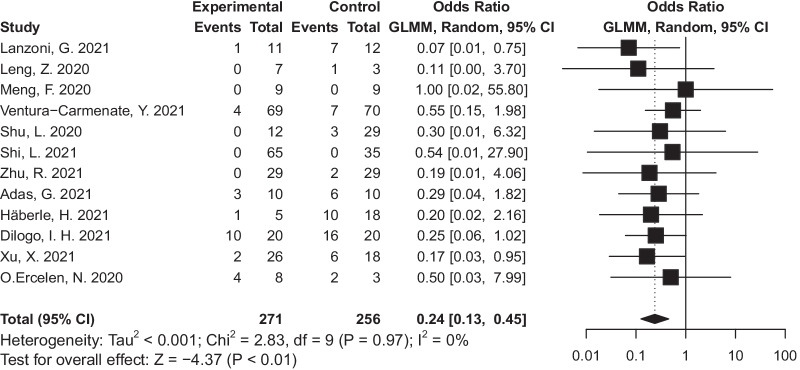
Forest plot of mortality: odds ratio (95% CI) and pooled estimates

There were four studies that were excluded from the mortality analysis on account of no reported mortality [17, 25] and duplication of data [23, 24]. Apart from this, there was one study [19] which reported mortality in the same study [18] after 1 year of follow-up and this was also excluded. Therefore, there were 12 clinical trials reporting mortality, in two [18, 27] of which, all participating patients survived. The total mortality rate was 16·13% (85/527), of which the stem cell group was 9.23% (25/271) and 23.44% (60/256) for the control group. Our meta-analysis showed stem cells therapy could decrease the mortality and the difference between stem cells and controls was statistically significant (OR = 0·24, 95% CI = 0·13 to 0·45, *P* < 0·01, I^2^ = 0%) in all clinical trials (Fig. 4). There was no significant publication bias according to the results of Egger’s test (*P* = 0.53) and Begg’s test (*P* = 0.58).

#### Hospitalization Time

**Fig. 5:**
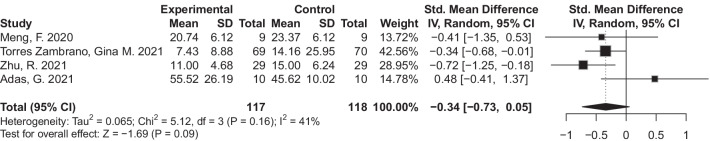
Forest plot of hospitalization time: odds ratio (95% CI) and pooled estimates

There were 6 [17, 20, 21, 23, 24, 27] studies that reported the duration from intervention to discharge or recovery, two of which was excluded due to being a duplicated report of the same clinical trial [23, 24]. The results of meta-analysis showed that hospitalization time in the stem cell treatment group was numerically shorter than that in control group, but there was no significant difference. (SMD = -0·34, 95% CI = -0·73 to 0·05, *P* = 0·09, I^2^ = 41%) (Fig. 5).

#### Laboratory parameters

**Fig. 6:**
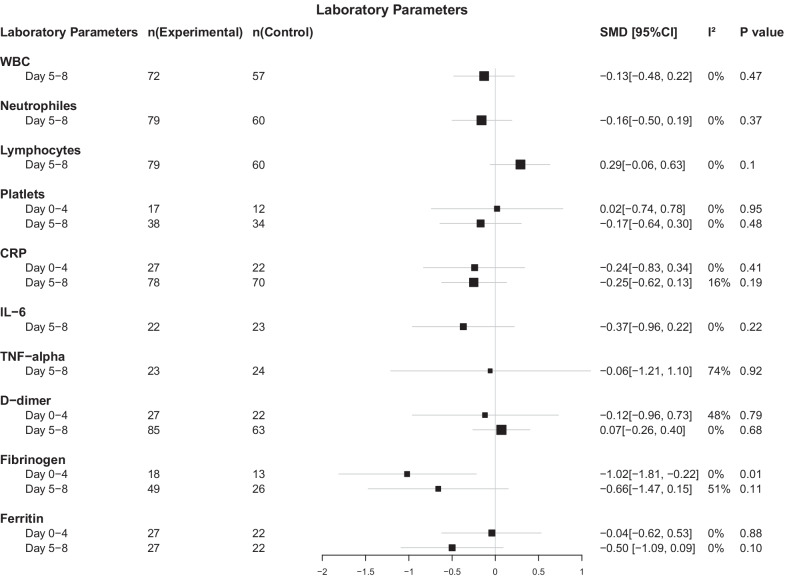
Forest plot of laboratory parameters: standard mean difference (95% CI) and pooled estimates

Six articles provided original data of laboratory parameters [15, 16, 21, 25, 27, 29]. In fifteen meta-analyses over two time periods for ten parameters, only fibrinogen level on day 0–4 was significantly lower in patients treated with stem cell than in the control group (SMD = -1·02, 95% CI = -1·81 to -0·22, *P* = 0·01, I^2^ = 0%). All other parameters showed no significant statistical difference between stem cell group and control group (Fig. 6). However, WBC (day 5–8), neutrophiles (day 5–8), lymphocytes (day 5–8), platelets (day 5–8), CRP (day 0–4 and day 5–8), IL-6 (day 5–8), TNF-α (day 5–8), D-dimer (day 0–4), fibrinogen (day 5–8), and ferritin (day 5–8) showed a numeric improvement after stem cell treatment. Among all parameters, platelets, and ferritin showed a better improvement on day 5–8 compared to that on day 0–4, although there is still no statistical difference. Detailed results of meta-analysis can be found in Additional file 8.

### Subgroup analysis

The results of all subgroup analysis were consistent with the original results (Table 4), indicating that study types of included articles didn’t influence the results. Detailed information can be found in Additional file 9.

**Table4:** Subgroup analyses results

Outcomes	Study Types	Effect Size [95%CI]	I^2^
AE	RCT	0.57 [0.21, 1.60]	69%
	NRCT	0.09 [0.00, 1.62]	–
	Combined Meta	0.39 [0.12, 1.33]	58%
Mortality	RCT	0.26 [0.12, 0.57]	0%
	NRCT	0.17 [0.05, 0.57]	0%
	Combined Meta	0.24 [0.13, 0.45]	0%
Length of hospital stay	RCT	− 0.30 [− 0.81, 0.20]	61%
	NRCT	− 0.41 [− 1.35, 0.53]	–
	Combined Meta	− 0.34 [− 0.73, 0.05]	41%
Neutrophils	RCT	− 0.24 [− 0.63, 0.16]	0%
	NRCT	0.08 [− 0.60, 0.76]	0%
	Combined Meta	− 0.16 [− 0.50, 0.19]	0%
Lymphocytes	RCT	0.25 [− 0.15, 0.64]	0%
	NRCT	0.41 [− 0.27, 1.10]	0%
	Combined Meta	0.29 [− 0.06, 0.63]	0%
PLT	RCT	− 0.39 [− 1.33, 0.54]	–
	NRCT	− 0.09 [− 0.64, 0.46]	0%
	Combined Meta	− 0.17 [− 0.64, 0.30]	0%
CRP	RCT	− 0.15 [− 0.56, 0.26]	0%
	NRCT	− 0.48 [− 1.43, 0.47]	59%
	Combined Meta	− 0.25 [− 0.62, 0.13]	16%
D-dimer	RCT	0.13 [− 0.24, 0.50]	0%
	NRCT	− 0.12 [− 1.15, 0.91]	39%
	Combined Meta	0.07 [− 0.26, 0.40]	0%

Subgroup analyses for AEs, mortality, length of hospital stay, neutrophils, lymphocytes, platelets, CRP and D-dimer according to type of study

## Discussion

Stem cells have been used extensively in clinical trials for the treatment of respiratory diseases due to their differentiation and regenerative properties [32–34]. Currently, stem cell therapy has become an optional therapy for critically ill patients with COVID-19 [8]. In our article, we conducted a systematic review and meta-analysis and included 17 clinical trials using stem cells to treat COVID-19 patients. The risk of bias analysis indicated that several articles have poor methodological quality and the results were therefore unsatisfactory. Some of these biases may be related to the experimental design, while others are probably due to the urgency of the situation. One article mentioned that the researchers were unable to obtain sufficient stem cells at the time as the treatment need was urgent; some patients who should have been randomized to the intervention group were distributed to the control group [31]. Overall, the results of stem cell therapy trials for COVID-19 showed that stem cells could reduce mortality without increasing length of hospital day, and occurrence of adverse events, but the number of high-quality RCTs is still limited.

In the present article, we regarded safety of treatment as the primary outcome, as safety is always the top concern for any new treatment. We have described the safety of stem cell treatment by AEs, and analyzed the data extracted from studies which reported the number of AEs and number of patients with AEs. Although the difference between experimental groups and control groups was not statistically significant, lower incidence of both AEs and SAEs in the experimental group was shown in this research. Besides, according to the articles, the SAEs occurring were not considered to be treatment-related. These results strongly suggest that the infusion of stem cell is safe.

In all included studies, MSCs were the predominant donor cells for COVID-19 patients. A previous meta-analysis has shown that MSCs therapy causes no significant AEs compared to the control [35], while in another meta-analysis, the occurrence of AEs in the experimental group was significantly lower compared to the control group [36]. It is worth noting that, in the synthesis process of these two studies, double-zero studies (Data in both groups is zero) were ignored, which will inevitably lead to the bias of the synthesized results [37]. Therefore, we conducted the synthesis by using a HNM, which is suitable for the meta-analysis of rare events, and takes double-zero studies into consideration.

In addition to statistical amelioration, the adverse events included in this study were consistent with the characteristics of stem cell therapy. Studies have verified that MSCs expressed a low level of MHC I molecules, and did not express MHC II molecules or costimulatory molecules B7-1, B7-2, or CD40 [38]. Besides, in vivo studies also have confirmed that allograft MSCs do not elicit typical immune responses [39]. These are consistent with the result that no severe allergic reactions were reported in the MSCs treatment group. In addition, in previous cohort study, there were also no AEs in the treatment group [40].

Though this meta-analysis supports the contention that stem cell therapy is safe, the potential risks associated with intravenous infusion itself should also be considered. According to a previous study, different MSCs products show different levels of high procoagulant tissue factor (TF) and may adversely trigger immediate blood-mediated inflammatory response (IBMIR) [41], which can lead to potentially fatal adverse events. Although not reported in studies we have reviewed, thrombosis and embolism has been reported in other diseases [42–45]. Especially for COVID-19 patients with high coagulopathy, the risk of thrombosis after cell infusion is a serious concern [46]. Besides, higher infusion volumes and higher cell doses of MSCs increases the risk of thrombosis, so it is worthwhile exploring how to reduce the risk of thrombosis and achieve therapeutic goals with limited cell counts in clinical practice [46]. Therefore, due to the differences in function and complexity among different stem cells and the variations in stem cell doses, the safety of stem cell therapy still needs to be evaluated by more and larger clinical studies in the future.

In clinical practice, MSCs were the most frequently used stem cell in pulmonary disease, as well as in COVID-19 patients [7, 47]. MSCs possesses differentiation and regenerative properties. They can repair lung injury by secreting HGF, VEGF, and KGF to promote the regeneration of type II alveolar epithelial cells [48]. Moreover, MSCs can be attracted to inflammatory sites by different chemokines and exert functions of regulating various immune cells (such as NK cells, dendritic cells, B cells, T cells, neutrophils, and macrophages) through direct contact and paracrine effects [49]. It is necessary to understand that the deterioration in condition of COVID-19 patients is mainly related to cytokine upregulation and excessive inflammatory response [7]. In critically ailing patients, cytokine storm leads to severe illness and end organ dysfunction, which has a high mortality rate [8].

For the first time, in the present review we have made a meta-analysis of laboratory parameters including WBC, neutrophils, lymphocytes, PLT, CRP, IL-6, TNF-α, D-dimer, ferritin, and fibrinogen according to the time at which they were measured. Although they can only serve as intermediate outcomes and cannot replace the outcome measures, their changes can reflect the course of the disease to some extent. Most parameters didn’t show a significant difference after treatment between stem cell therapy and control group, however, the combined data still suggest stem cell treatment tends to reduce inflammation and benefited the patients, as all parameters indeed showed an amelioration trend. A large number of experimental animal studies and early clinical studies have also confirmed that MSCs play an efficient role in the treatment of COVID-19 or ARDS by inducing an anti-inflammatory response [32].

Pulmonary function is also an important indicator for evaluating stem cell therapy for COVID-19. Although we could not do a meta-analysis of oxygen saturation (SaO2 or SpO2) and oxygenation index (PaO2/FIO2) based on the available data, several systematic reviews still suggest that stem cell therapy is able to improve pulmonary function [7, 36, 47, 50]. Changes in some radiographic images also confirm this [17–20, 25, 26, 31], which is potentially related to decreased fibrosis and inflammation.

Efficacy assessment of stem cell therapy on mortality showed no deaths directly related to stem cell infusion in any of the included studies. The mortality rate of COVID-19 varies due to the different sample size and follow-up time. Our analysis indicated that stem cell therapy could significantly decrease mortality rate of COVID-19. Several systematic reviews have also confirmed that stem cell therapy reduces mortality of COVID-19 patients, which is highly likely to be a result of reduced systematic inflammation [8, 36, 47, 50, 51].

We also performed a meta-analysis of length of hospital stay. Although there is no significant decrease in the stem cell group compared to control group, the hospitalization time of COVID-19 patients was shortened numerically. Three articles [15, 26, 31] reported that MSCs could reduce the average recovery or improvement time significantly, while Wang et al. [36] found that only the average time of recovery was shortened significantly in the MSCs group, and the length of hospital stay showed no difference, which is mainly due to the small sample sizes and non-uniform admission criteria. Additionally, several studies pointed out that stem cell treatment achieved better clinical manifestations [16, 19, 31] or achieved a higher cumulative symptom remission rate [20], which suggests that stem cell therapy may improve the clinical condition of patients with COVID-19.

There were some limitations in our study. First, the relatively small number of eligible studies after multiple screenings and limited sample sizes may reduce the power of the conclusions. By March, 2022, there are still 168 registered clinical trials unpublished, ongoing, or terminated (see Additional file 10.). Second, patients’ demographic and clinical characteristics, stem cell type, way of administration, dose and frequency of treatment, and definitions of AE vary in different studies. Third, the number of high-quality studies was extremely limited, and we also did not conduct subgroup analyses for different qualities of literature. All these differences may affect the reliability of the result. More studies are needed to confirm the conclusion.

## Conclusion

Stem cell therapy is a safe and efficient way to manage COVID-19 patients. It may substantially reduce mortality of COVID-19 patients without increasing the occurrence of adverse events and length of hospital stay. Meanwhile, the meta-analysis of laboratory parameters showed that inflammatory factors tended to decrease after stem cell infusion, providing possible insights into the mechanism of stem cell therapy for COVID-19. More clinical trials are needed to confirm the conclusion, and we also need a standardized clinical protocol to guide stem cells treatment for COVID-19.

## Supplementary Information

**Additional file 1.** Search Strategy. Search strategy used in the eight databases.

**Additional file 2.** List of excluded studies. 176 studies were excluded after reviewing the full texts. Detailed information of these articles are shown.

**Additional file 3.** Quality Assessment of 10 RCTs. 10 RCTs included in the systematic review were assessed for literature quality using the Risk-of-bias Tool 1.0. a: Risk of bias graph: authors' judgements’ about each risk of bias item presented as percentages. b: Risk of bias summary: authors' judgements about each risk of bias item.

**Additional file 4.** Quality Assessment of 7 NRCTs. 7 NRCTs included in the systematic review were assessed for literature quality using the MINORS instrument.

**Additional file 5.** Characteristics of Involved Studies. Specific information of 17 articles included: country, study design, number of patients, treatment of intervention group and control group.

**Additional file 6.** All Adverse Events in Involved Studies. All adverse events (AEs), AEs related to treatment and numbers of patients with AEs reported in involved studies between experimental and control groups.

**Additional file 7.** Serious Adverse Events. All serious adverse events reported in involved studies between experimental and control groups.

**Additional file 8.** Results of Meta-analysis of Laboratory parameters. Results of Meta-analysis of WBC, neutrophiles, lymphocytes, platelets, CRP, IL-6, TNF-α, D-dimer, fibrinogen and ferritin in day 0–4 or day 5–8. a. Forest plot of WBC (day5–8): Std. Mean Difference (95% CI) and pooled estimates. b. Forest plot of neutrophils (day5–8): Std. Mean Difference (95% CI) and pooled estimates. c. Forest plot of lymphocytes (day5–8): Std. Mean Difference (95% CI) and pooled estimates. d. Forest plot of PLT (day0–4): Std. Mean Difference (95% CI) and pooled estimates. e. Forest plot of PLT (day5-8): Std. Mean Difference (95% CI) and pooled estimates. f. Forest plot of CRP (day0–4): Std. Mean Difference (95% CI) and pooled estimates. g. Forest plot of CRP (day5–8): Std. Mean Difference (95% CI) and pooled estimates. h. Forest plot of IL-6 (day5–8): Std. Mean Difference (95% CI) and pooled estimates. I. Forest plot of TNF-α (day5–8): Std. Mean Difference (95% CI) and pooled estimates. j. Forest plot of D-dimer (day0–4): Std. Mean Difference (95% CI) and pooled estimates. k. Forest plot of D-dimer (day5–8): Std. Mean Difference (95% CI) and pooled estimates. l. Forest plot of fibrinogen (day0–4): Std. Mean Difference (95% CI) and pooled estimates. m. Forest plot of fibrinogen (day5–8): Std. Mean Difference (95% CI) and pooled estimates. n. Forest plot of ferritin (day0–4): Std. Mean Difference (95% CI) and pooled estimates. o. Forest plot of ferritin (day5–8): Std. Mean Difference (95% CI) and pooled estimates.

**Additional file 9.** Results of subgroup analysis of outcomes. Results of Meta-analysis of AEs, mortality, hospital stay, neutrophils, lymphocytes, platelets, CRP and D-dimer according to the type of studies. a. Subgroup analysis of AEs b. Subgroup analysis of mortality c. Subgroup analysis of length of hospital stay d. Subgroup analysis of neutrophils (day5–8) e. Subgroup analysis of lymphocytes (day5–8) f. Subgroup analysis of PLT (day5–8) g. Subgroup analysis of CRP (day5–8) h. Subgroup analysis of D-dimer (day5–8).

**Additional file 10.** Ongoing Clinical Trials of Stem Cells Therapy for COVID-19. We searched clinical trials from the ClinicalTrials.gov database and the World Health Organization's International Clinical Trials Registry Platform (WHO ICTRP) and combined the data from the Global Coronavirus COVID-19 Clinical Trial Tracker (https://covid19-trials.com/). Totally 177 studies were shown that related to stem cell and derivatives therapy for COVID-19 patients. The database search for this systematic review was conducted in March, 2022. The country with the most registered trials is the United States. Removing 9 registration numbers that we included, the recruitment status showed that there were 52 not yet recruiting, 7 authorized, 72 recruiting, 9 active, not recruiting and 16 completed but unpublished among the 168 registered clinical trials. The remaining 12 trials were suspended, terminated, withdrawn, available or of unknown status. Moreover, not all clinical trials that show completion have reported results. Based on these results, stem cell treatments for COVID-19 did not have sufficient completed Clinical trial data and were still in the experimental stage. Current published clinical trials had been collected in the systematic review and our evaluation implied that stem cell-based therapy might decrease mortality and improve clinical manifestation of patients diagnosed COVID-19.

## Data Availability

The datasets generated during and/or analyzed during the current study are available from the corresponding author on reasonable request.

## Abbreviations

WHO: World Health Organization
DONs: Disease Outbreak News
COVID-19: Coronavirus Disease
SARS-CoV-2: Severe Acute Respiratory Syndrome Coronavirus 2
SARS: Severe Acute Respiratory Syndrome
MERS: Middle East Respiratory Syndrome
ARDS: Acute Respiratory Distress Syndrome
CRP: C-reactive protein
MSC: Mesenchymal Stem Cells
CNKI: China National Knowledge Internet
AE: Adverse Event
RCT: Randomized Controlled Trial
NRCT: Non-Randomized Controlled Trial
PRISMA: Preferred Reporting Items for Systematic Reviews and Meta-Analyses
SAE: Severe Adverse Event
WBC: White blood cells
PLT: Platelets
TNF-α: Tumor necrosis factor α
MINORS: Methodological index for non-randomized studies
SMD: Standardized mean difference
RR: Relative risk
CI: Confidence intervals
FEM: Fixed effect model
REM: Random effect model
HNM: Hypergeometric normal model
UC-MSCs: Umbilical cord mesenchymal stem cells
BM-MSCs: Human bone marrow mesenchymal stem cells
MenSCs: Menstrual blood-derived mesenchymal stem cells
PB‑NHESC‑C: Peripheral blood non‑hematopoietic enriched stem cell cocktail
CTCAE: Common Terminology Criteria for Adverse Events
