# Supplementary material for: Safety and efficiency of stem cell therapy for COVID-19: a systematic review and meta-analysis
Source: Glob Health Res Policy. 2022 Jun 23;7:19. doi: 10.1186/s41256-022-00251-5 (PMC9217728; doi:10.1186/s41256-022-00251-5)
Supplement: Supplementary file 1 — Additional file 1. Search Strategy. Search strategy used in the eight databases. [file 41256_2022_251_MOESM1_ESM.docx]

**Additional file 1. Search Strategy**

| **Search Strategy in Pubmed** | |
| --- | --- |
| **Search** | **Query** |
| #1 | “SARS-CoV-2” [MeSH] OR “SARS-COV-2” [Title] OR “Severe Acute Respiratory Syndrome Coronavirus 2”[Title] OR “Coronavirus Disease 2019 Virus” [Title] OR “2019 Novel Coronavirus” [Title] OR “SARS-CoV-2 Virus” [Title] OR “2019-nCoV” [Title] OR “COVID-19 Virus” [Title] OR “SARS Coronavirus 2” [Title] |
| #2 | “COVID-19” [MeSH] OR “COVID-19” [Title] OR “COVID-19 Virus Disease”[Title] OR “COVID19” [Title] OR “COVID-19 Virus Infection” [Title] OR “2019-nCoV Infection” [Title] OR “Coronavirus Disease 2019” [Title] OR “SARS Coronavirus 2 Infection” [Title] OR “SARS CoV 2 Infection” [Title] OR “COVID-19 Pandemic” [Title] |
| #3 | #1 OR #2 |
| #4 | "Cell- and Tissue-Based Therapy"[Mesh] OR (((”Cell AND Tissue Based Therapy”[Title/Abstract]) OR (“Tissue Therapy”[Title/Abstract])) OR (”Therapy, Tissue”[Title/Abstract])) OR (”Cell Therapy”[Title/Abstract]) |
| #5 | (((((((“Stem Cell”[Title/Abstract]) OR (“Progenitor Cells”[Title/Abstract])) OR (“Progenitor Cell”[Title/Abstract])) OR (“Mother Cells”[Title/Abstract])) OR (“Mother Cell”[Title/Abstract])) OR (“Colony Forming Unit”[Title/Abstract])) OR (“Colony Forming Units”[Title/Abstract])) OR (“Colony-Forming Units”[Title/Abstract]) OR "Stem Cells"[Mesh] OR "Stem Cells"[Title/Abstract]  OR "Stem Cell Transplantation"[Mesh] OR "Stem Cell Transplantation"[Title/Abstract] OR "Stromal Cells"[Mesh] OR "Stromal Cells"[Title/Abstract] OR “mesenchymal cell”[Title/Abstract] OR “mesenchymal cells”[Title/Abstract] OR “mononuclear cell”[Title/Abstract] OR “mononuclear cells”[Title/Abstract] OR “cord blood cell”[Title/Abstract] OR “cord blood cells”[Title/Abstract] OR “regenerative cell”[Title/Abstract] OR “regenerative cells”[Title/Abstract] OR "stromal cell"[Title/Abstract] OR "stromal cells"[Title/Abstract]  OR “Mesenchymal Stem Cells”[Mesh] OR “Mesenchymal Stem Cell”[Title/Abstract] OR “Bone Marrow Mesenchymal Stem Cells”[Title/Abstract] OR “Bone Marrow Mesenchymal Stem Cell”[Title/Abstract] OR “Bone Marrow Stromal Cells”[Title/Abstract] OR “Bone Marrow Stromal Cell”[Title/Abstract] OR “Multipotent Bone Marrow Stromal Cell”[Title/Abstract] OR “Multipotent Bone Marrow Stromal Cells”[Title/Abstract] OR “Adipose-Derived Mesenchymal Stem Cells”[Title/Abstract] OR “Adipose Derived Mesenchymal Stem Cells”[Title/Abstract] OR “Adipose-Derived Mesenchymal Stromal Cells”[Title/Abstract] OR “Adipose Derived Mesenchymal Stromal Cells”[Title/Abstract] OR “Adipose-Derived Mesenchymal Stem Cell”[Title/Abstract] OR “Adipose Derived Mesenchymal Stem Cell”[Title/Abstract] OR “Adipose Tissue-Derived Mesenchymal Stem Cell”[Title/Abstract] OR “Adipose Tissue Derived Mesenchymal Stem Cell”[Title/Abstract] OR “Adipose Tissue-Derived Mesenchymal Stem Cells”[Title/Abstract] OR “Adipose Tissue Derived Mesenchymal Stem Cells”[Title/Abstract] OR “Adipose Tissue-Derived Mesenchymal Stromal Cells”[Title/Abstract] OR “Adipose Tissue Derived Mesenchymal Stromal Cells”[Title/Abstract] OR “Adipose Tissue-Derived Mesenchymal Stromal Cell”[Title/Abstract] OR “Adipose Tissue Derived Mesenchymal Stromal Cell”[Title/Abstract] OR “Mesenchymal Stromal Cells”[Title/Abstract] OR “Mesenchymal Stromal Cell”[Title/Abstract] OR “Multipotent Mesenchymal Stromal Cells”[Title/Abstract] OR “Multipotent Mesenchymal Stromal Cell”[Title/Abstract] OR “Mesenchymal Progenitor Cell”[Title/Abstract] OR “Mesenchymal Progenitor Cells”[Title/Abstract] OR “Wharton Jelly Cells”[Title/Abstract] OR “Wharton's Jelly Cells”[Title/Abstract] OR “Wharton's Jelly Cell”[Title/Abstract] OR “Whartons Jelly Cells”[Title/Abstract] OR “Bone Marrow Stromal Stem Cells”[Title/Abstract]  OR “human embryonic stem cells”[Title/Abstract] OR “cardiosphere-derived cells”[Title/Abstract] OR “peripheral blood mononuclear cells”[Title/Abstract] OR “bone marrow-derived multipotent adult progenitor cells”[Title/Abstract] OR “immunity and matrix-regulatory cells”[Title/Abstract] OR “umbilical cord-derived mesenchymal stem cells”[Title/Abstract] OR “bone marrow-derived mesenchymal stem cells”[Title/Abstract] OR “adipose-derived mesenchymal stem cells”[Title/Abstract]  OR “Adult Stem Cells”[Title/Abstract] OR “Adult Germline Stem Cells”[Title/Abstract] OR “Induced Pluripotent Stem Cells”[Title/Abstract] OR “Fetal Stem Cells”[Title/Abstract] OR “Hematopoietic Stem Cells”[Title/Abstract] OR “Hemangioblasts”[Title/Abstract] OR “Lymphoid Progenitor Cells"[Title/Abstract] OR “Myeloid Progenitor Cells"[Title/Abstract] OR “Peripheral Blood Stem Cells”[Title/Abstract] OR “Myoblasts”[Title/Abstract] OR “Myoblasts, Cardiac”[Title/Abstract] OR “Myoblasts, Skeletal"[Title/Abstract] OR “Myoblasts, Smooth Muscle”[Title/Abstract] OR “Neoplastic Stem Cells”[Title/Abstract] OR “Embryonal Carcinoma Stem Cells”[Title/Abstract] OR “Neural Stem Cells”[Title/Abstract] OR “Oligodendrocyte Precursor Cells”[Title/Abstract] OR “Oogonial Stem Cells”[Title/Abstract] OR “Pluripotent Stem Cells”[Title/Abstract] OR “Embryonic Stem Cells"[Title/Abstract] OR “Induced Pluripotent Stem Cells”[Title/Abstract] OR “Side-Population Cells”[Title/Abstract] OR “Totipotent Stem Cells”[Title/Abstract] OR “Adult Stem Cells”[Mesh] OR “Adult Germline Stem Cells”[Mesh] OR “Induced Pluripotent Stem Cells”[Mesh] OR “Fetal Stem Cells”[Mesh] OR “Hematopoietic Stem Cells”[Mesh] OR “Hemangioblasts”[Mesh] OR “Lymphoid Progenitor Cells"[Mesh] OR “Myeloid Progenitor Cells"[Mesh] OR “Peripheral Blood Stem Cells”[Mesh] OR “Myoblasts”[Mesh] OR “Myoblasts, Cardiac”[Mesh] OR “Myoblasts, Skeletal"[Mesh] OR “Myoblasts, Smooth Muscle”[Mesh] OR “Neoplastic Stem Cells”[Mesh] OR “Embryonal Carcinoma Stem Cells”[Mesh] OR “Neural Stem Cells”[Mesh] OR “Oligodendrocyte Precursor Cells”[Mesh] OR “Oogonial Stem Cells”[Mesh] OR “Pluripotent Stem Cells”[Mesh] OR “Embryonic Stem Cells"[Mesh] OR “Induced Pluripotent Stem Cells”[Mesh] OR “Side-Population Cells”[Mesh] OR “Totipotent Stem Cells”[Mesh] |
| #6 | #4 AND #5 |
| #7 | #3 AND #6 |

| **Search Strategy in Embase** | |
| --- | --- |
| **Search** | **Query** |
| #1 | 'sars cov 2'/exp OR 'sars cov 2':ab,ti OR 'severe acute respiratory syndrome coronavirus 2':ab,ti OR 'coronavirus disease 2019 virus':ab,ti OR '2019 novel coronavirus':ab,ti OR 'sars-cov-2 virus':ab,ti OR '2019 ncov':ab,ti OR 'covid-19 virus':ab,ti OR 'sars coronavirus 2':ab,ti |
| #2 | 'covid 19'/exp OR 'covid 19':ab,ti OR 'covid-19 virus disease':ab,ti OR 'covid-19 virus infection':ab,ti OR covid19:ab,ti OR '2019-ncov infection':ab,ti OR 'coronavirus disease 2019':ab,ti OR 'sars coronavirus 2 infection':ab,ti OR 'sars cov 2 infection':ab,ti OR 'covid-19 pandemic':ab,ti |
| #3 | 'cell'/exp AND 'tissue-based therapy' OR (cell:ab,ti AND 'tissue-based therapy':ab,ti) OR 'tissue therapy':ab,ti OR 'therapy, tissue':ab,ti OR 'cell therapy':ab,ti |
| #4 | 'stem cell':ab,ti OR 'progenitor cells':ab,ti OR 'progenitor cell':ab,ti OR 'mother cells':ab,ti OR 'mother cell':ab,ti OR 'colony forming unit':ab,ti OR 'colony forming units':ab,ti OR 'colony-forming units':ab,ti OR 'stem cells'/exp OR 'stem cells':ab,ti |
| #5 | 'stem cell transplantation'/exp OR 'stem cell transplantation':ab,ti OR 'stromal cells'/exp OR 'mesenchymal cell':ab,ti OR 'mesenchymal cells':ab,ti OR 'mononuclear cell':ab,ti OR 'mononuclear cells':ab,ti OR 'cord blood cell':ab,ti OR 'cord blood cells':ab,ti OR 'regenerative cell':ab,ti OR 'regenerative cells':ab,ti OR 'stromal cell':ab,ti OR 'stromal cells':ab,ti |
| #6 | 'mesenchymal stem cells'/exp OR 'mesenchymal stem cell':ab,ti OR 'bone marrow mesenchymal stem cells':ab,ti OR 'bone marrow mesenchymal stem cell':ab,ti OR 'bone marrow stromal cells':ab,ti OR 'bone marrow stromal cell':ab,ti OR 'multipotent bone marrow stromal cell':ab,ti OR 'multipotent bone marrow stromal cells':ab,ti OR 'adipose-derived mesenchymal stem cells':ab,ti OR 'adipose derived mesenchymal stem cells':ab,ti OR 'adipose-derived mesenchymal stromal cells':ab,ti OR 'adipose derived mesenchymal stromal cells':ab,ti OR 'adipose-derived mesenchymal stem cell':ab,ti OR 'adipose derived mesenchymal stem cell':ab,ti OR 'adipose tissue-derived mesenchymal stem cell':ab,ti OR 'adipose tissue derived mesenchymal stem cell':ab,ti OR 'adipose tissue-derived mesenchymal stem cells':ab,ti OR 'adipose tissue derived mesenchymal stem cells':ab,ti OR 'adipose tissue-derived mesenchymal stromal cells':ab,ti OR 'adipose tissue derived mesenchymal stromal cells':ab,ti OR 'adipose tissue-derived mesenchymal stromal cell':ab,ti OR 'adipose tissue derived mesenchymal stromal cell':ab,ti OR 'mesenchymal stromal cells':ab,ti OR 'mesenchymal stromal cell':ab,ti OR 'multipotent mesenchymal stromal cells':ab,ti OR 'multipotent mesenchymal stromal cell':ab,ti OR 'mesenchymal progenitor cell':ab,ti OR 'mesenchymal progenitor cells':ab,ti OR 'wharton jelly cells':ab,ti OR 'whartons jelly cell':ab,ti OR 'whartons jelly cells':ab,ti OR 'bone marrow stromal stem cells':ab,ti |
| #7 | 'human embryonic stem cells':ab,ti OR 'cardiosphere-derived cells':ab,ti OR 'peripheral blood mononuclear cells':ab,ti OR 'bone marrow-derived multipotent adult progenitor cells':ab,ti OR 'immunity and matrix-regulatory cells':ab,ti OR 'angiotensin-converting enzyme 2 mesenchymal stem cells':ab,ti OR 'umbilical cord-derived mesenchymal stem cells':ab,ti OR 'bone marrow-derived mesenchymal stem cells':ab,ti OR 'adipose-derived mesenchymal stem cells':ab,ti OR 'perinatal tissues mesenchymal stem cells':ab,ti |
| #8 | 'adult stem cells':ab,ti OR 'adult germline stem cells':ab,ti OR 'fetal stem cells':ab,ti OR 'hematopoietic stem cells':ab,ti OR 'hemangioblasts':ab,ti OR 'lymphoid progenitor cells':ab,ti OR 'myeloid progenitor cells':ab,ti OR 'peripheral blood stem cells':ab,ti OR 'myoblasts':ab,ti OR 'myoblasts, cardiac':ab,ti OR 'myoblasts, skeletal':ab,ti OR 'myoblasts, smooth muscle':ab,ti OR 'neoplastic stem cells':ab,ti OR 'embryonal carcinoma stem cells':ab,ti OR 'neural stem cells':ab,ti OR 'oligodendrocyte precursor cells':ab,ti OR 'oogonial stem cells':ab,ti OR 'pluripotent stem cells':ab,ti OR 'embryonic stem cells':ab,ti OR 'induced pluripotent stem cells':ab,ti OR 'side-population cells':ab,ti OR 'totipotent stem cells':ab,ti OR 'adult stem cells'/exp OR 'adult germline stem cells'/exp OR 'fetal stem cells'/exp OR 'hematopoietic stem cells'/exp OR 'hemangioblasts'/exp OR 'lymphoid progenitor cells'/exp OR 'myeloid progenitor cells'/exp OR 'peripheral blood stem cells'/exp OR 'myoblasts'/exp OR 'myoblasts, cardiac'/exp OR 'myoblasts, skeletal'/exp OR 'myoblasts, smooth muscle'/exp OR 'neoplastic stem cells'/exp OR 'embryonal carcinoma stem cells'/exp OR 'neural stem cells'/exp OR 'oligodendrocyte precursor cells'/exp OR 'oogonial stem cells'/exp OR 'pluripotent stem cells'/exp OR 'embryonic stem cells'/exp OR 'induced pluripotent stem cells'/exp OR 'side-population cells'/exp OR 'totipotent stem cells'/exp |
| #9 | #1 OR #2 |
| #10 | #3 OR #4 OR #5 OR #6 OR #7 OR #8 |
| #11 | #9 AND #10 |

| **Search Strategy in Web of Science** | |
| --- | --- |
| **Search** | **Query** |
| #1 | TI=(“SARS-CoV-2” OR “Severe Acute Respiratory Syndrome Coronavirus 2” OR “Coronavirus Disease 2019 Virus” OR “2019 Novel Coronavirus” OR “SARS-CoV-2 Virus” OR “2019-nCoV” OR “COVID-19 Virus” OR “SARS Coronavirus 2”) |
| #2 | AB=(“SARS-CoV-2” OR “Severe Acute Respiratory Syndrome Coronavirus 2” OR “Coronavirus Disease 2019 Virus” OR “2019 Novel Coronavirus” OR “SARS-CoV-2 Virus” OR “2019-nCoV” OR “COVID-19 Virus” OR “SARS Coronavirus 2”) |
| #3 | #1 OR #2 |
| #4 | TI=(“COVID-19” OR “COVID-19 Virus Disease” OR “COVID19” OR “COVID-19 Virus Infection” OR “2019-nCoV Infection” OR “Coronavirus Disease 2019” OR “SARS Coronavirus 2 Infection” OR “SARS CoV 2 Infection” OR “COVID-19 Pandemic”) |
| #5 | AB=(“COVID-19” OR “COVID-19 Virus Disease” OR “COVID19” OR “COVID-19 Virus Infection” OR “2019-nCoV Infection” OR “Coronavirus Disease 2019” OR “SARS Coronavirus 2 Infection” OR “SARS CoV 2 Infection” OR “COVID-19 Pandemic”) |
| #6 | #4 OR #5 |
| #7 | #3 OR #6 |
| #8 | TI=(“Cell- and Tissue-Based Therapy” OR “Cell AND Tissue Based Therapy” OR “Tissue Therapy” OR “Therapy, Tissue” OR “Cell Therapy”) |
| #9 | AB=(“Cell- and Tissue-Based Therapy” OR “Cell AND Tissue Based Therapy” OR “Tissue Therapy” OR “Therapy, Tissue” OR “Cell Therapy”) |
| #10 | #8 OR #9 |
| #11 | TI=(“Stem Cell” OR “Progenitor Cells” OR “Progenitor Cell” OR “Mother Cells” OR “Mother Cell” OR “Colony Forming Unit” OR “Colony Forming Units” OR “Colony-Forming Units” OR "Stem Cells" OR "Stem Cells" OR "Stem Cell Transplantation" OR "Stem Cell Transplantation" OR "Stromal Cells" OR "Stromal Cells" OR “mesenchymal cell” OR “mesenchymal cells” OR “mononuclear cell” OR “mononuclear cells” OR “cord blood cell” OR “cord blood cells” OR “regenerative cell” OR “regenerative cells” OR "stromal cell" OR "stromal cells" OR “Mesenchymal Stem Cell” OR “Bone Marrow Mesenchymal Stem Cells” OR “Bone Marrow Mesenchymal Stem Cell” OR “Bone Marrow Stromal Cells” OR “Bone Marrow Stromal Cell” OR “Multipotent Bone Marrow Stromal Cell” OR “Multipotent Bone Marrow Stromal Cells” OR “Adipose-Derived Mesenchymal Stem Cells” OR “Adipose Derived Mesenchymal Stem Cells” OR “Adipose-Derived Mesenchymal Stromal Cells” OR “Adipose Derived Mesenchymal Stromal Cells” OR “Adipose-Derived Mesenchymal Stem Cell” OR “Adipose Derived Mesenchymal Stem Cell” OR “Adipose Tissue-Derived Mesenchymal Stem Cell” OR “Adipose Tissue Derived Mesenchymal Stem Cell” OR “Adipose Tissue-Derived Mesenchymal Stem Cells” OR “Adipose Tissue Derived Mesenchymal Stem Cells” OR “Adipose Tissue-Derived Mesenchymal Stromal Cells” OR “Adipose Tissue Derived Mesenchymal Stromal Cells” OR “Adipose Tissue-Derived Mesenchymal Stromal Cell” OR “Adipose Tissue Derived Mesenchymal Stromal Cell” OR “Mesenchymal Stromal Cells” OR “Mesenchymal Stromal Cell” OR “Multipotent Mesenchymal Stromal Cells” OR “Multipotent Mesenchymal Stromal Cell” OR “Mesenchymal Progenitor Cell” OR “Mesenchymal Progenitor Cells” OR “Wharton Jelly Cells” OR “Wharton's Jelly Cells” OR “Wharton's Jelly Cell” OR “Whartons Jelly Cells” OR “Bone Marrow Stromal Stem Cells” OR “human embryonic stem cells” OR “cardiosphere-derived cells” OR “peripheral blood mononuclear cells” OR “bone marrow-derived multipotent adult progenitor cells” OR “immunity and matrix-regulatory cells” OR “angiotensin-converting enzyme 2 mesenchymal stem cells” OR “umbilical cord-derived mesenchymal stem cells” OR “bone marrow-derived mesenchymal stem cells” OR “adipose-derived mesenchymal stem cells” OR “perinatal tissues mesenchymal stem cells”OR “Adult Stem Cells” OR “Adult Germline Stem Cells” OR “Induced Pluripotent Stem Cells” OR “Fetal Stem Cells” OR “Hematopoietic Stem Cells” OR “Hemangioblasts” OR “Lymphoid Progenitor Cells" OR “Myeloid Progenitor Cells" OR “Peripheral Blood Stem Cells” OR “Myoblasts” OR “Myoblasts, Cardiac” OR “Myoblasts, Skeletal" OR “Myoblasts, Smooth Muscle” OR “Neoplastic Stem Cells” OR “Embryonal Carcinoma Stem Cells” OR “Neural Stem Cells” OR “Oligodendrocyte Precursor Cells” OR “Oogonial Stem Cells” OR “Pluripotent Stem Cells” OR “Embryonic Stem Cells" OR “Induced Pluripotent Stem Cells” OR “Side-Population Cells” OR “Totipotent Stem Cells” OR “Adult Stem Cells” OR “Adult Germline Stem Cells” OR “Induced Pluripotent Stem Cells” OR “Fetal Stem Cells” OR “Hematopoietic Stem Cells” OR “Hemangioblasts” OR “Lymphoid Progenitor Cells" OR “Myeloid Progenitor Cells" OR “Peripheral Blood Stem Cells” OR “Myoblasts” OR “Myoblasts, Cardiac” OR “Myoblasts, Skeletal" OR “Myoblasts, Smooth Muscle” OR “Neoplastic Stem Cells” OR “Embryonal Carcinoma Stem Cells” OR “Neural Stem Cells” OR “Oligodendrocyte Precursor Cells” OR “Oogonial Stem Cells” OR “Pluripotent Stem Cells” OR “Embryonic Stem Cells" OR “Induced Pluripotent Stem Cells” OR “Side-Population Cells” OR “Totipotent Stem Cells”) |
| #12 | AB=(“Stem Cell” OR “Progenitor Cells” OR “Progenitor Cell” OR “Mother Cells” OR “Mother Cell” OR “Colony Forming Unit” OR “Colony Forming Units” OR “Colony-Forming Units” OR "Stem Cells" OR "Stem Cells" OR "Stem Cell Transplantation" OR "Stem Cell Transplantation" OR "Stromal Cells" OR "Stromal Cells" OR “mesenchymal cell” OR “mesenchymal cells” OR “mononuclear cell” OR “mononuclear cells” OR “cord blood cell” OR “cord blood cells” OR “regenerative cell” OR “regenerative cells” OR "stromal cell" OR "stromal cells" OR “Mesenchymal Stem Cell” OR “Bone Marrow Mesenchymal Stem Cells” OR “Bone Marrow Mesenchymal Stem Cell” OR “Bone Marrow Stromal Cells” OR “Bone Marrow Stromal Cell” OR “Multipotent Bone Marrow Stromal Cell” OR “Multipotent Bone Marrow Stromal Cells” OR “Adipose-Derived Mesenchymal Stem Cells” OR “Adipose Derived Mesenchymal Stem Cells” OR “Adipose-Derived Mesenchymal Stromal Cells” OR “Adipose Derived Mesenchymal Stromal Cells” OR “Adipose-Derived Mesenchymal Stem Cell” OR “Adipose Derived Mesenchymal Stem Cell” OR “Adipose Tissue-Derived Mesenchymal Stem Cell” OR “Adipose Tissue Derived Mesenchymal Stem Cell” OR “Adipose Tissue-Derived Mesenchymal Stem Cells” OR “Adipose Tissue Derived Mesenchymal Stem Cells” OR “Adipose Tissue-Derived Mesenchymal Stromal Cells” OR “Adipose Tissue Derived Mesenchymal Stromal Cells” OR “Adipose Tissue-Derived Mesenchymal Stromal Cell” OR “Adipose Tissue Derived Mesenchymal Stromal Cell” OR “Mesenchymal Stromal Cells” OR “Mesenchymal Stromal Cell” OR “Multipotent Mesenchymal Stromal Cells” OR “Multipotent Mesenchymal Stromal Cell” OR “Mesenchymal Progenitor Cell” OR “Mesenchymal Progenitor Cells” OR “Wharton Jelly Cells” OR “Wharton's Jelly Cells” OR “Wharton's Jelly Cell” OR “Whartons Jelly Cells” OR “Bone Marrow Stromal Stem Cells” OR “human embryonic stem cells” OR “cardiosphere-derived cells” OR “peripheral blood mononuclear cells” OR “bone marrow-derived multipotent adult progenitor cells” OR “immunity and matrix-regulatory cells” OR “angiotensin-converting enzyme 2 mesenchymal stem cells” OR “umbilical cord-derived mesenchymal stem cells” OR “bone marrow-derived mesenchymal stem cells” OR “adipose-derived mesenchymal stem cells” OR “perinatal tissues mesenchymal stem cells”OR “Adult Stem Cells” OR “Adult Germline Stem Cells” OR “Induced Pluripotent Stem Cells” OR “Fetal Stem Cells” OR “Hematopoietic Stem Cells” OR “Hemangioblasts” OR “Lymphoid Progenitor Cells" OR “Myeloid Progenitor Cells" OR “Peripheral Blood Stem Cells” OR “Myoblasts” OR “Myoblasts, Cardiac” OR “Myoblasts, Skeletal" OR “Myoblasts, Smooth Muscle” OR “Neoplastic Stem Cells” OR “Embryonal Carcinoma Stem Cells” OR “Neural Stem Cells” OR “Oligodendrocyte Precursor Cells” OR “Oogonial Stem Cells” OR “Pluripotent Stem Cells” OR “Embryonic Stem Cells" OR “Induced Pluripotent Stem Cells” OR “Side-Population Cells” OR “Totipotent Stem Cells” OR “Adult Stem Cells” OR “Adult Germline Stem Cells” OR “Induced Pluripotent Stem Cells” OR “Fetal Stem Cells” OR “Hematopoietic Stem Cells” OR “Hemangioblasts” OR “Lymphoid Progenitor Cells" OR “Myeloid Progenitor Cells" OR “Peripheral Blood Stem Cells” OR “Myoblasts” OR “Myoblasts, Cardiac” OR “Myoblasts, Skeletal" OR “Myoblasts, Smooth Muscle” OR “Neoplastic Stem Cells” OR “Embryonal Carcinoma Stem Cells” OR “Neural Stem Cells” OR “Oligodendrocyte Precursor Cells” OR “Oogonial Stem Cells” OR “Pluripotent Stem Cells” OR “Embryonic Stem Cells" OR “Induced Pluripotent Stem Cells” OR “Side-Population Cells” OR “Totipotent Stem Cells”) |
| #13 | #11 OR #12 |
| #14 | #10 OR #13 |
| #15 | #14 AND #7 |

| **Search Strategy in Chochrane** | |
| --- | --- |
| **Search** | **Query** |
| #1 | (SARS-COV-2):ti,ab,kw OR (”Severe Acute Respiratory Syndrome Coronavirus 2“):ti,ab,kw OR (”Coronavirus Disease 2019 Virus“):ti,ab,kw OR (“2019 Novel Coronavirus”):ti,ab,kw OR ("SARS-CoV-2 Virus"):ti,ab,kw |
| #2 | ("COVID-19 Virus Disease"):ti,ab,kw OR ("2019-nCoV"):ti,ab,kw OR ("COVID-19 Virus"):ti,ab,kw OR ("SARS Coronavirus 2"):ti,ab,kw OR ("COVID-19"):ti,ab,kw |
| #3 | MeSH descriptor: [SARS-CoV-2] this term only |
| #4 | MeSH descriptor: [COVID-19] this term only |
| #5 | ("COVID-19 Virus Infection"):ti,ab,kw OR ("2019-nCoV Infection"):ti,ab,kw OR ("Coronavirus Disease 2019"):ti,ab,kw OR ("SARS Coronavirus 2 Infection"):ti,ab,kw OR ("SARS CoV 2 Infection"):ti,ab,kw |
| #6 | (“COVID-19 Pandemic”):ti,ab,kw OR (COVID19):ti,ab,kw |
| #7 | (“Cell AND Tissue Based Therapy”):ti,ab,kw OR (“Tissue Therapy”):ti,ab,kw OR (“Therapy, Tissue”):ti,ab,kw OR (“Cell Therapy”):ti,ab,kw |
| #8 | MeSH descriptor: [Cell- and Tissue-Based Therapy] this term only |
| #9 | #1 OR #2 OR #3 OR #4 OR #5 OR #6 |
| #10 | MeSH descriptor: [Stem Cells] this term only |
| #11 | (“Progenitor Cells”):ti,ab,kw OR (“Progenitor Cell”):ti,ab,kw OR (“Mother Cells”):ti,ab,kw OR (“Mother Cell”):ti,ab,kw OR ("Stem Cells"):ti,ab,kw |
| #12 | ("Colony Forming Unit"):ti,ab,kw OR ("Colony Forming Units"):ti,ab,kw OR ("Colony-Forming Units"):ti,ab,kw OR ("mesenchymal cell"):ti,ab,kw AND ("mesenchymal cells"):ti,ab,kw |
| #13 | MeSH descriptor: [Stem Cells] this term only |
| #14 | MeSH descriptor: [Stem Cell Transplantation] this term only |
| #15 | MeSH descriptor: [Stromal Cells] this term only |
| #16 | ("mononuclear cell"):ti,ab,kw OR ("mononuclear cells"):ti,ab,kw OR ("cord blood cell"):ti,ab,kw OR ("cord blood cells"):ti,ab,kw OR ("regenerative cell"):ti,ab,kw |
| #17 | ("regenerative cells"):ti,ab,kw OR ("stromal cell"):ti,ab,kw OR ("stromal cells"):ti,ab,kw OR ("Stromal Cells"):ti,ab,kw |
| #18 | #7 OR #8 OR #10 OR #11 OR #12 OR #14 OR #15 OR #16 OR #17 |
| #19 | (“Mesenchymal Stem Cell”):ti,ab,kw OR (“Bone Marrow Mesenchymal Stem Cells”):ti,ab,kw OR (“Bone Marrow Mesenchymal Stem Cell”):ti,ab,kw OR (“Bone Marrow Stromal Cells”):ti,ab,kw OR (“Bone Marrow Stromal Cell”):ti,ab,kw |
| #20 | (“Multipotent Bone Marrow Stromal Cell”):ti,ab,kw OR (“Multipotent Bone Marrow Stromal Cells”):ti,ab,kw OR (“Adipose-Derived Mesenchymal Stem Cells”):ti,ab,kw OR (“Adipose Derived Mesenchymal Stem Cells”):ti,ab,kw OR (“Adipose-Derived Mesenchymal Stromal Cells”):ti,ab,kw |
| #21 | (“Adipose Derived Mesenchymal Stromal Cells”):ti,ab,kw OR (“Adipose-Derived Mesenchymal Stem Cell”):ti,ab,kw OR (“Adipose-Derived Mesenchymal Stem Cell”):ti,ab,kw OR (“Adipose Tissue-Derived Mesenchymal Stem Cell”):ti,ab,kw OR (“Adipose Tissue Derived Mesenchymal Stem Cell”):ti,ab,kw |
| #22 | (“Adipose Tissue-Derived Mesenchymal Stem Cells”):ti,ab,kw OR (“Adipose Tissue Derived Mesenchymal Stem Cells”):ti,ab,kw OR (“Adipose Tissue-Derived Mesenchymal Stromal Cells”):ti,ab,kw OR (“Adipose Tissue Derived Mesenchymal Stromal Cells”):ti,ab,kw OR (“Adipose Tissue-Derived Mesenchymal Stromal Cell”):ti,ab,kw |
| #23 | (“Adipose Tissue Derived Mesenchymal Stromal Cell”):ti,ab,kw OR (“Mesenchymal Stromal Cells”):ti,ab,kw OR (“Mesenchymal Stromal Cell”):ti,ab,kw OR (“Multipotent Mesenchymal Stromal Cells”):ti,ab,kw OR (“Multipotent Mesenchymal Stromal Cell”):ti,ab,kw |
| #24 | (“Mesenchymal Progenitor Cell”):ti,ab,kw OR (“Mesenchymal Progenitor Cells”):ti,ab,kw OR (“Wharton Jelly Cells”):ti,ab,kw OR (“Wharton's Jelly Cells”):ti,ab,kw OR (“Wharton's Jelly Cell”):ti,ab,kw |
| #25 | (“Whartons Jelly Cells”):ti,ab,kw OR (“Bone Marrow Stromal Stem Cells”):ti,ab,kw OR (“human embryonic stem cells”):ti,ab,kw OR (“cardiosphere-derived cells”):ti,ab,kw OR (“peripheral blood mononuclear cells”):ti,ab,kw |
| #26 | (“bone marrow-derived multipotent adult progenitor cells”):ti,ab,kw OR (“immunity and matrix-regulatory cells”):ti,ab,kw OR (“angiotensin-converting enzyme 2 mesenchymal stem cells”):ti,ab,kw OR (“umbilical cord-derived mesenchymal stem cells”):ti,ab,kw OR (“bone marrow-derived mesenchymal stem cells”):ti,ab,kw |
| #27 | (“adipose-derived mesenchymal stem cells”):ti,ab,kw OR (“perinatal tissues mesenchymal stem cells”):ti,ab,kw OR (“Adult Stem Cells”):ti,ab,kw OR (“Adult Germline Stem Cells”):ti,ab,kw OR (“Induced Pluripotent Stem Cells”):ti,ab,kw |
| #28 | (“Fetal Stem Cells”):ti,ab,kw OR (“Hematopoietic Stem Cells”):ti,ab,kw OR (“Hemangioblasts”):ti,ab,kw OR (“Lymphoid Progenitor Cells"):ti,ab,kw OR (“Myeloid Progenitor Cells"):ti,ab,kw |
| #29 | (“Peripheral Blood Stem Cells”):ti,ab,kw OR (“Myoblasts”):ti,ab,kw OR (“Myoblasts, Cardiac”):ti,ab,kw OR (“Myoblasts, Smooth Muscle”):ti,ab,kw OR ("Myoblasts, Skeletal"):ti,ab,kw |
| #30 | (“Neoplastic Stem Cells”):ti,ab,kw OR (“Embryonal Carcinoma Stem Cells”):ti,ab,kw OR (“Neural Stem Cells”):ti,ab,kw OR (“Oligodendrocyte Precursor Cells”):ti,ab,kw OR (“Oogonial Stem Cells”):ti,ab,kw |
| #31 | ("Pluripotent Stem Cells"):ti,ab,kw OR ("Embryonic Stem Cells"):ti,ab,kw OR (“Induced Pluripotent Stem Cells”):ti,ab,kw OR (“Side-Population Cells”):ti,ab,kw OR ("Totipotent Stem Cells"):ti,ab,kw |
| #32 | #19 OR #20 OR #21 OR #22 OR #23 OR #24 OR #25 OR #26 OR #27 OR #28 OR #29 OR #30 OR #31 |
| #33 | MeSH descriptor: [Adult Stem Cells] this term only |
| #34 | MeSH descriptor: [Hematopoietic Stem Cells] this term only |
| #35 | MeSH descriptor: [Induced Pluripotent Stem Cells] this term only |
| #36 | MeSH descriptor: [Hemangioblasts] this term only |
| #37 | MeSH descriptor: [Myeloid Progenitor Cells] this term only |
| #38 | MeSH descriptor: [Peripheral Blood Stem Cells] this term only |
| #39 | MeSH descriptor: [Myoblasts] this term only |
| #40 | MeSH descriptor: [Myoblasts, Cardiac] this term only |
| #41 | MeSH descriptor: [Myoblasts, Skeletal] this term only |
| #42 | MeSH descriptor: [Neoplastic Stem Cells] this term only |
| #43 | MeSH descriptor: [Neural Stem Cells] this term only |
| #44 | MeSH descriptor: [Oligodendrocyte Precursor Cells] this term only |
| #45 | MeSH descriptor: [Pluripotent Stem Cells] this term only |
| #46 | MeSH descriptor: [Embryonic Stem Cells] this term only |
| #47 | MeSH descriptor: [Induced Pluripotent Stem Cells] this term only |
| #48 | #33 OR #34 OR #35 OR #36 OR #37 OR #38 OR #39 OR #40 OR #41 OR #42 OR #43 OR #44 OR #45 OR #46 OR #47 |
| #49 | #18 OR #32 OR #48 |
| #50 | #9 AND #49 |

| **Search Strategy in WanFang** | |
| --- | --- |
| **Search** | **Query** |
|  | （题名或关键词:(新冠肺炎) or 题名或关键词:(新型冠状病毒) or 题名或关键词:(新型冠状病毒肺炎)） and （题名或关键词:(细胞治疗) or 题名或关键词:(干细胞) or 题名或关键词:(间充质基质细胞) or 题名或关键词:(造血干细胞移植) or 题名或关键词:(造血干细胞) or 题名或关键词:(干细胞移植) or 题名或关键词:(肿瘤干细胞) or 题名或关键词:(原癌基因蛋白质类c-kit) or 题名或关键词:(胚胎干细胞) or 题名或关键词:(间质干细胞移植) or 题名或关键词:(多能干细胞) or 题名或关键词:(干细胞因子) or 题名或关键词:(外周血干细胞移植) or 题名或关键词:(成体干细胞) or 题名或关键词:(多潜能干细胞) or 题名或关键词:(脐血干细胞移植) or 题名或关键词:(红系前体细胞) or 题名或关键词:(骨髓祖代细胞) or 题名或关键词:(造血干细胞动员) or 题名或关键词:(集落形成单位测定) or 题名或关键词:(骨髓净化) or 题名或关键词:(肿瘤干细胞测定) or 题名或关键词:(全能干细胞) or 题名或关键词:(淋巴系祖细胞) or 题名或关键词:(胎儿干细胞) or 题名或关键词:(神经干细胞) or 题名或关键词:(干细胞研究) or 题名或关键词:(干细胞壁龛) or 题名或关键词:(卵原干细胞) or 题名或关键词:(外周血干细胞) or 题名或关键词:(细胞自我更新) or 题名或关键词:(胚胎性癌干细胞) or 题名或关键词:(外周血干细胞) or 题名或关键词:(人胚胎干细胞) or 题名或关键词:(诱导多能干细胞) or 题名或关键词:(胚胎性癌干细胞) or 题名或关键词:(卵原干细胞) or 题名或关键词:(成体生殖干细胞) or 题名或关键词:(小鼠胚胎干细胞) or 题名或关键词:(人胚胎干细胞) or 题名或关键词:(细胞不对称分裂) or 题名或关键词:(成血-血管干细胞)） |

| **Search Strategy in VIP** | |
| --- | --- |
| **Search** | **Query** |
|  | ((((((((((((((((((((((((((((((((((((((((((题名或关键词=细胞治疗 OR 题名或关键词=干细胞) OR 题名或关键词=间充质基质细胞) OR 题名或关键词=造血干细胞移植) OR 题名或关键词=造血干细胞) OR 题名或关键词=干细胞移植) OR 题名或关键词=肿瘤干细胞) OR (题名或关键词=“原癌基因蛋白质类c AND ( NOT 题名或关键词=kit”))) OR 题名或关键词=胚胎干细胞) OR 题名或关键词=间质干细胞移植) OR 题名或关键词=多能干细胞) OR 题名或关键词=干细胞因子) OR 题名或关键词=外周血干细胞移植) OR 题名或关键词=成体干细胞) OR 题名或关键词=多潜能干细胞) OR 题名或关键词=脐血干细胞移植) OR 题名或关键词=红系前体细胞) OR 题名或关键词=骨髓祖代细胞) OR 题名或关键词=造血干细胞动员) OR 题名或关键词=集落形成单位测定) OR 题名或关键词=骨髓净化) OR 题名或关键词=肿瘤干细胞测定) OR 题名或关键词=全能干细胞) OR 题名或关键词=淋巴系祖细胞) OR 题名或关键词=胎儿干细胞) OR 题名或关键词=神经干细胞) OR 题名或关键词=干细胞研究) OR 题名或关键词=干细胞壁龛) OR 题名或关键词=卵原干细胞) OR 题名或关键词=外周血干细胞) OR 题名或关键词=细胞自我更新) OR 题名或关键词=胚胎性癌干细胞) OR 题名或关键词=外周血干细胞) OR 题名或关键词=人胚胎干细胞) OR 题名或关键词=诱导多能干细胞) OR 题名或关键词=胚胎性癌干细胞) OR 题名或关键词=卵原干细胞) OR 题名或关键词=成体生殖干细胞) OR 题名或关键词=小鼠胚胎干细胞) OR 题名或关键词=人胚胎干细胞) OR 题名或关键词=细胞不对称分裂) OR (题名或关键词=“成血 AND ( NOT 题名或关键词=血管干细胞”))) AND ((((题名或关键词="COVID-19" OR 题名或关键词=新冠肺炎) OR 题名或关键词=新型冠状病毒肺炎) OR 题名或关键词=新型冠状病毒) OR 题名或关键词="SARS-COV-2")) |

| **Search Strategy in CNKI** | |
| --- | --- |
| **Search** | **Query** |
| #1 | TKA=(‘细胞治疗‘+‘干细胞’+‘间充质基质细胞’+‘造血干细胞移植’+‘造血干细胞’+‘干细胞移植’+‘肿瘤干细胞’+‘肿瘤干细胞’+‘原癌基因蛋白质类c-kit’+‘胚胎干细胞’+‘间质干细胞移植’+‘多能干细胞’+‘干细胞因子’+‘外周血干细胞移植’+‘成体干细胞’+‘多潜能干细胞’+‘脐血干细胞移植’+‘红系前体细胞’+‘骨髓祖代细胞’+‘造血干细胞动员’+‘集落形成单位测定’+‘骨髓净化’+‘肿瘤干细胞测定’+‘全能干细胞’+‘淋巴系祖细胞’+‘胎儿干细胞’+‘神经干细胞’+‘干细胞研究’+‘干细胞壁龛’+'外周血干细胞’+‘诱导多能干细胞’+‘胚胎性癌干细胞’+‘卵原干细胞’+‘成体生殖干细胞’+‘细胞自我更新’+‘人胚胎干细胞’+‘细胞不对称分裂’+‘成血-血管干细胞’+‘人胚胎干细胞’+‘小鼠胚胎干细胞‘) |
| #2 | TKA=(‘新型冠状病毒肺炎‘+’新冠肺炎‘+’新型冠状病毒‘) |

| **Search Strategy in SinoMed** | |
| --- | --- |
| **Search** | **Query** |
| #1 | "新冠肺炎"[不加权:扩展] OR "新型冠状病毒"[不加权:扩展] OR "新型冠状病毒肺炎"[不加权:扩展] |
| #2 | "细胞治疗"[不加权:扩展] |
| #3 | "干细胞"[不加权:扩展] OR "间充质基质细胞"[不加权:扩展] OR "间充质基质细胞"[不加权:扩展] OR "造血干细胞移植"[不加权:扩展] OR "造血干细胞移植"[不加权:扩展] OR "造血干细胞"[不加权:扩展] OR "造血干细胞"[不加权:扩展] OR "干细胞移植"[不加权:扩展] OR "干细胞移植"[不加权:扩展] OR "肿瘤干细胞"[不加权:扩展] OR "肿瘤干细胞"[不加权:扩展] OR "肿瘤干细胞"[不加权:扩展] OR "肿瘤干细胞"[不加权:扩展] OR "原癌基因蛋白质类c-kit"[不加权:扩展] OR "原癌基因蛋白质类c-kit"[不加权:扩展] OR "胚胎干细胞"[不加权:扩展] OR "胚胎干细胞"[不加权:扩展] OR "间质干细胞移植"[不加权:扩展] OR "间质干细胞移植"[不加权:扩展] OR "多能干细胞"[不加权:扩展] OR "多能干细胞"[不加权:扩展] OR "干细胞因子"[不加权:扩展] OR "外周血干细胞移植"[不加权:扩展] OR "外周血干细胞移植"[不加权:扩展] OR "外周血干细胞移植"[不加权:扩展] OR "成体干细胞"[不加权:扩展] OR "成体干细胞"[不加权:扩展] OR "多潜能干细胞"[不加权:扩展] OR "多潜能干细胞"[不加权:扩展] OR "脐血干细胞移植"[不加权:扩展] OR "脐血干细胞移植"[不加权:扩展] OR "脐血干细胞移植"[不加权:扩展] OR "脐血干细胞移植"[不加权:扩展] OR "脐血干细胞移植"[不加权:扩展] OR "红系前体细胞"[不加权:扩展] OR "红系前体细胞"[不加权:扩展] OR "骨髓祖代细胞"[不加权:扩展] OR "造血干细胞动员"[不加权:扩展] OR "造血干细胞动员"[不加权:扩展] OR "集落形成单位测定"[不加权:扩展] OR "骨髓净化"[不加权:扩展] OR "肿瘤干细胞测定"[不加权:扩展] OR "肿瘤干细胞测定"[不加权:扩展] OR "肿瘤干细胞测定"[不加权:扩展] OR "全能干细胞"[不加权:扩展] OR "全能干细胞"[不加权:扩展] OR "淋巴系祖细胞"[不加权:扩展] OR "胎儿干细胞"[不加权:扩展] OR "神经干细胞"[不加权:扩展] OR "干细胞研究"[不加权:扩展] OR "干细胞壁龛"[不加权:扩展] OR "卵原干细胞"[不加权:扩展] OR "外周血干细胞"[不加权:扩展] OR "细胞自我更新"[不加权:扩展] OR "胚胎性癌干细胞"[不加权:扩展] OR "外周血干细胞"[不加权:扩展] OR "人胚胎干细胞"[不加权:扩展] OR "诱导多能干细胞"[不加权:扩展] OR "胚胎性癌干细胞"[不加权:扩展] OR "胚胎性癌干细胞"[不加权:扩展] OR "卵原干细胞"[不加权:扩展] OR "卵原干细胞"[不加权:扩展] OR "成体生殖干细胞"[不加权:扩展] OR "细胞自我更新"[不加权:扩展] OR "小鼠胚胎干细胞"[不加权:扩展] OR "小鼠胚胎干细胞"[不加权:扩展] OR "小鼠胚胎干细胞"[不加权:扩展] OR "人胚胎干细胞"[不加权:扩展] OR "人胚胎干细胞"[不加权:扩展] OR "细胞不对称分裂"[不加权:扩展] OR "成血-血管干细胞"[不加权:扩展] OR "人胚胎干细胞"[不加权:扩展] OR "小鼠胚胎干细胞"[不加权:扩展] |
| #4 | #2 OR #3 |
| #5 | #1 AND #4 |
