# Supplementary material for: Safety and efficiency of stem cell therapy for COVID-19: a systematic review and meta-analysis
Source: Glob Health Res Policy. 2022 Jun 23;7:19. doi: 10.1186/s41256-022-00251-5 (PMC9217728; doi:10.1186/s41256-022-00251-5)
Supplement: Supplementary file 2 — Additional file 2. List of excluded studies. 176 studies were excluded after reviewing the full texts. Detailed information of these articles are shown. [file 41256_2022_251_MOESM2_ESM.docx]

**Additional file 2. List of excluded studies**

| **Author (year)/ Trail ID** | **Reason for exclusion** |
| --- | --- |
| Ali G (2021)^1^ | Case report |
| Atanackovic D (2021)^2^ | Case report |
| Ciccocioppo R (2021)^3^ | Case report |
| Dalla Via V (2021)^4^ | Case report |
| Eckard, AR (2021)^5^ | Case report |
| Foss FM (2020)^6^ | Case report |
| Gupta AK (2021)^7^ | Case report |
| Gupta AK (2021)^8^ | Case report |
| Iglesias M (2021).^9^ | Case report |
| Jarmoliński T (2021)^10^ | Case report |
| Khan S (2021)^11^ | Case report |
| Krajewski J (2021)^12^ | Case report |
| Liang B (2020)^13^ | Case report |
| Lu J (2021)^14^ | Case report |
| Nazon C (2020)^15^ | Case report |
| Peng H (2020)^16^ | Case report |
| Primorac D (2021)^17^ | Case report |
| Rynda A (2021)^18^ | Case report |
| Sahin AS (2021)^19^ | Case report |
| Sari S (2021)^20^ | Case report |
| Senegaglia AC (2021)^21^ | Case report |
| Silva KND (2021)^22^ | Case report |
| Zang LY (2020)^23^ | Case report |
| Soler Rich R (2020)^24^ | Case report |
| Tang L (2020)^25^ | Case report |
| Tao J (2020)^26^ | Case report |
| Teixiera J (2021)^27^ | Case report |
| Yilmaz R (2020)^28^ | Case report |
| Zengin R (2020)^29^ | Case report |
| Zhang Q (2021)^30^ | Case report |
| Zhang Y (2020)^31^ | Case report |
| Zhu Y (2020)^32^ | Case report |
| Zhu Y (2020)^33^ | Case report |
| Gu LM (2020)^34^ | Case report |
| Li P (2020)^35^ | Case series |
| Hashemian SM (2021)^36^ | Case series |
| Averbuch D (2021)^37^ | Case series |
| Barkama R (2020)^38^ | Case series |
| Brown SA (2021)^39^ | Case series |
| Chen X (2020)^40^ | Case series |
| Feng Y (2020)^41^ | Case series |
| Guo Z (2020)^42^ | Case series |
| Kesari S (2021)^43^ | Case series |
| N OE (2021)^44^ | Case series |
| Sadeghi B (2021)^45^ | Case series |
| Sanchez-Guijo F (2020)^46^ | Case series |
| Singh S (2020)^47^ | Case series |
| Yiğenoğlu TN (2021)^48^ | Case series |
| Zhou J (2020)^49^ | Case series |
| Feng G (2021)^50^ | Cohort study |
| IRCT20200413047063N2 ^51^ | Study protocol |
| ACTRN12620000478910 ^52^ | Study protocol |
| ACTRN12620000612910 ^53^ | Study protocol |
| ChiCTR2000029569 ^54^ | Study protocol |
| ChiCTR2000029572 ^55^ | Study protocol |
| ChiCTR2000031319 ^56^ | Study protocol |
| ChiCTR2000029580 ^57^ | Study protocol |
| CTRI/2020/10/028250 ^58^ | Study protocol |
| CTRI/2021/09/036645 ^59^ | Study protocol |
| EUCTR2019-002688-89-ES ^60^ | Study protocol |
| EUCTR2020-001266-11-ES ^61^ | Study protocol |
| EUCTR2020-001450-22-ES ^62^ | Study protocol |
| EUCTR2020-002193-27-ES ^63^ | Study protocol |
| EUCTR2020-001505-22-ES ^64^ | Study protocol |
| EUCTR2020-001364-29-ES ^65^ | Study protocol |
| EUCTR2020-002772-12-FR ^66^ | Study protocol |
| EUCTR2020-001577-70-IT ^67^ | Study protocol |
| Gorman E (2020)^68^ | Study protocol |
| IRCT20140528017891N8 ^69^ | Study protocol |
| IRCT20140911019125N8 ^70^ | Study protocol |
| IRCT20160809029275N1 ^71^ | Study protocol |
| IRCT20180619040147N7 ^72^ | Study protocol |
| IRCT20190717044241N3 ^73^ | Study protocol |
| IRCT20200217046526N2 ^74^ | Study protocol |
| IRCT20200325046860N2 ^75^ | Study protocol |
| IRCT20200413047063N1 ^76^ | Study protocol |
| IRCT20200421047150N1 ^77^ | Study protocol |
| IRCT20200426047206N2 ^78^ | Study protocol |
| IRCT20200621047859N4 ^79^ | Study protocol |
| IRCT20211012052743N1 ^80^ | Study protocol |
| ISRCTN33578935 ^81^ | Study protocol |
| NCT04525378 ^82^ | Study protocol |
| NCT04581954 ^83^ | Study protocol |
| NCT04324996 ^84^ | Study protocol |
| NCT04482699 ^85^ | Study protocol |
| NCT04457609 ^86^ | Study protocol |
| NCT04341610 ^87^ | Study protocol |
| NCT04428801 ^88^ | Study protocol |
| NCT04348461 ^89^ | Study protocol |
| NCT04346368 ^90^ | Study protocol |
| NCT04333368 ^91^ | Study protocol |
| NCT04339660 ^92^ | Study protocol |
| NCT04615429 ^93^ | Study protocol |
| NCT04366323 ^94^ | Study protocol |
| NCT04565665 ^95^ | Study protocol |
| NCT04390139 ^96^ | Study protocol |
| NCT04625738 ^97^ | Study protocol |
| NCT04437823 ^98^ | Study protocol |
| NCT04657458 ^99^ | Study protocol |
| NCT04399889 ^100^ | Study protocol |
| NCT04537351 ^101^ | Study protocol |
| NCT04444271 ^102^ | Study protocol |
| NCT04573270 ^103^ | Study protocol |
| NCT04611256 ^104^ | Study protocol |
| NCT04646603 ^105^ | Study protocol |
| NCT04466098 ^106^ | Study protocol |
| NCT04614025 ^107^ | Study protocol |
| NCT04629105 ^108^ | Study protocol |
| NCT04371601 ^109^ | Study protocol |
| NCT04390152 ^110^ | Study protocol |
| NCT04429763 ^111^ | Study protocol |
| NCT04336254 ^112^ | Study protocol |
| NCT04602442 ^113^ | Study protocol |
| NCT04299152 ^114^ | Study protocol |
| NCT04445220 ^115^ | Study protocol |
| NCT04361942 ^116^ | Study protocol |
| NCT04288102 ^117^ | Study protocol |
| NCT04494386 ^118^ | Study protocol |
| NCT04490486 ^119^ | Study protocol |
| NCT04865107 ^120^ | Study protocol |
| NCT04797975 ^121^ | Study protocol |
| NCT05132972 ^122^ | Study protocol |
| NCT05125562 ^123^ | Study protocol |
| NCT05017298 ^124^ | Study protocol |
| NCT05116761 ^125^ | Study protocol |
| NCT05019287 ^126^ | Study protocol |
| NCT05122234 ^127^ | Study protocol |
| NCT04713878 ^128^ | Study protocol |
| NCT05126563 ^129^ | Study protocol |
| NCT04909879 ^130^ | Study protocol |
| NCT04905836 ^131^ | Study protocol |
| NCT04992247 ^132^ | Study protocol |
| NCT04728698 ^133^ | Study protocol |
| NCT04869397 ^134^ | Study protocol |
| NCT04753476 ^135^ | Study protocol |
| Payares-Herrera C (2021)^136^ | Study protocol |
| RPCEC00000322 ^137^ | Study protocol |
| Rynda A (2021)^138^ | Study protocol |
| RBR-65trt53 ^139^ | Study protocol |
| Agrawal N (2020)^140^ | Without stem cell therapy |
| Agrawal N (2021)^141^ | Without stem cell therapy |
| Ali G (2021)^142^ | Without stem cell therapy |
| Antonio PM (2021)^143^ | Without stem cell therapy |
| Basar R (2021)^144^ | Without stem cell therapy |
| Nishiga M (2021)^145^ | Without stem cell therapy |
| Ortiz de Landazuri I (2020)^146^ | Without stem cell therapy |
| Pera M (2020)^147^ | Without stem cell therapy |
| Pérez-Martínez A (2021)^148^ | Without stem cell therapy |
| Pérez-Martínez A (2021)^149^ | Without stem cell therapy |
| Pérez-Martínez A (2021)^150^ | Without stem cell therapy |
| Piñana JL (2022)^151^ | Without stem cell therapy |
| Pineda-Terreros B (2021)^152^ | Without stem cell therapy |
| Qatawneh M (2021)^153^ | Without stem cell therapy |
| Rajendra A (2021)^154^ | Without stem cell therapy |
| Ram R (2021)^155^ | Without stem cell therapy |
| Ratajczak MZ (2021)^156^ | Without stem cell therapy |
| Leticia RC (2020)^157^ | Without stem cell therapy |
| Salehi MS (2021)^158^ | Without stem cell therapy |
| Sbragia E (2021)^159^ | Without stem cell therapy |
| Senegaglia AC (2021)^160^ | Without stem cell therapy |
| Shah GL (2020)^161^ | Without stem cell therapy |
| Sharma A (2021)^162^ | Without stem cell therapy |
| Shetty AK (2021)^163^ | Without stem cell therapy |
| Sivapalan R (2021)^164^ | Without stem cell therapy |
| Stanley K (2021)^165^ | Without stem cell therapy |
| Tufan AC (2021)^166^ | Without stem cell therapy |
| Vaiasicca S (2021)^167^ | Without stem cell therapy |
| Vaiasicca S (2020)^168^ | Without stem cell therapy |
| Vishnevetsky A (2021)^169^ | Without stem cell therapy |
| Zuo W (2021)^170^ | Without stem cell therapy |
| Fan XL (2020)^171^ | Without stem cell therapy |
| Hou XJ (2021)^172^ | Without stem cell therapy |
| Saleh M (2021)^173^ | Without blank control |
| Wu J (2020)^174^ | Without blank control |
| Andrade HD (2021)^175^ | Without blank control |
| Pérez-Martínez A (2021)^176^ | Without blank control |

**References of excluded studies**

1. Ali G, Altareb M, Chaudhri N. CML-005: Outcome of Haploidentical Hematopoietic Stem Cell Transplantation with a Donor and Recipient with SARS-CoV-2 Infection. *Clinical Lymphoma, Myeloma and Leukemia* 2021; **21**: S325-S6.

2. Atanackovic D, Luetkens T, Avila SV, et al. Anti-sars-cov-2 immune responses in patients receiving an allogeneic stem cell or organ transplant. *Vaccines* 2021; **9**(7).

3. Ciccocioppo R, Gibellini D, Astori G, et al. The immune modulatory effects of umbilical cord-derived mesenchymal stromal cells in severe COVID-19 pneumonia. *Stem Cell Research and Therapy* 2021; **12**(1).

4. Dalla Via V, von Rotz M, Bättig V, et al. Possible Reactivation of SARS-CoV-2 in a Patient with Acute Myeloid Leukemia Undergoing Allogeneic Hematopoietic Stem Cell Transplantation: a Case Report. *SN Comprehensive Clinical Medicine* 2021; **3**(10): 2011-5.

5. Eckard AR, Borow KM, Mack EH, Burke E, Atz AM. Remestemcel-l therapy for covid-19-associated multisystem inflammatory syndrome in children. *Pediatrics* 2021; **147**(5).

6. Foss FM, Rubinowitz A, Landry ML, et al. Attenuated Novel SARS Coronavirus 2 Infection in an Allogeneic Hematopoietic Stem Cell Transplant Patient on Ruxolitinib. *Clinical Lymphoma, Myeloma and Leukemia* 2020; **20**(11): 720-3.

7. Gupta AK, Ramachandran M, Gupta A, et al. Allogeneic hematopoietic stem cell transplant after COVID-19 infection and its effect on the antibody titers to SARS-CoV-2. *Pediatric Transplantation* 2021.

8. Gupta AK, Ramachandran M, Meena JP, et al. Robust and sustained antibody response to SARS-CoV-2 in a child pre and post autologous hematopoietic stem cell transplant. *Pediatric Blood and Cancer* 2021; **68**(5).

9. Iglesias M, Butrón P, Torre-Villalvazo I, et al. Mesenchymal stem cells for the compassionate treatment of severe acute respiratory distress syndrome due to COVID 19. *Aging and Disease* 2021; **12**(2): 360-70.

10. Jarmoliński T, Matkowska-Kocjan A, Rosa M, et al. SARS-CoV-2 viral clearance during bone marrow aplasia after allogeneic hematopoietic stem cell transplantation—A case report. *Pediatric Transplantation* 2021; **25**(5).

11. Khan S, English S, Hodgins S, et al. MANUFACTURING FRESHLY CULTURED UMBILICAL CORD-DERIVED MESENCHYMAL STROMAL CELLS (UC-MSCS) FOR A PHASE 1, MULTIPLE-DOSE CLINICAL TRIAL FOR COVID-19-INDUCED ACUTE RESPIRATORY DISTRESS SYNDROME (ARDS). *Cytotherapy* 2021; **23**(5): S55.

12. Krajewski J, Chen J, Motiani J, et al. Hematopoietic stem cell transplant in two pediatric patients testing positive for SARS-CoV-2: A case report. *Pediatric Transplantation* 2021.

13. Liang B, Chen J, Li T, et al. Clinical remission of a critically ill COVID-19 patient treated by human umbilical cord mesenchymal stem cells: A case report. *Medicine* 2020; **99**(31): e21429.

14. Lu J, Xie ZY, Zhu DH, Li LJ. Human menstrual blood-derived stem cells as immunoregulatory therapy in COVID-19: A case report and review of the literature. *World Journal of Clinical Cases* 2021; **9**(7): 1705-13.

15. Nazon C, Velay A, Radosavljevic M, Fafi-Kremer S, Paillard C. Coronavirus disease 2019 3 months after hematopoietic stem cell transplant: A pediatric case report. *Pediatric Blood and Cancer* 2020; **67**(9).

16. Peng H, Gong T, Huang X, et al. A synergistic role of convalescent plasma and mesenchymal stem cells in the treatment of severely ill COVID-19 patients: A clinical case report. *Stem Cell Research and Therapy* 2020; **11**(1).

17. Primorac D, Stipić SS, Strbad M, et al. Compassionate mesenchymal stem cell treatment in a severe COVID-19 patient: A case report. *Croatian Medical Journal* 2021; **62**(3): 201-9.

18. Rynda A, Hancharou A, Antonevich N, et al. IMMUNOLOGIC EFFICACY OF POOLED MESENCHYMAL STEM CELLS IN PATIENTS WITH SEVERE COVID-19-ASSOCIATED PNEUMONIA. *Annals of Allergy Asthma & Immunology* 2021; **127**(5): S39-S.

19. Sahin AS, Kaya E, Turgut G, Dolay K, Kocatas A. Mesenchymal Stem Cell Transplantation for COVID-19 Treatment in a Puerperium Period in Intensive Care Unit. *Turkish Journal of Anaesthesiology and Reanimation* 2021; **49**(5): 428-9.

20. Sari S, Mengi T, Tahta Y. Our Experience with Mesenchymal Stem Cells in Patients with COVID-19. *Mediterranean Journal of Infection, Microbes and Antimicrobials* 2021; **10**.

21. Senegaglia AC, Rebelatto CLK, Franck CL, et al. Combined Use of Tocilizumab and Mesenchymal Stromal Cells in the Treatment of Severe Covid-19: Case Report. *Cell Transplantation* 2021; **30**.

22. Silva KND, Pinheiro PCG, Gobatto ALN, et al. Immunomodulatory and Anti-fibrotic Effects Following the Infusion of Umbilical Cord Mesenchymal Stromal Cells in a Critically Ill Patient With COVID-19 Presenting Lung Fibrosis: A Case Report. *Frontiers in Medicine* 2021; **8**.

23. Zhang Liyuan, Du Yongguo, Wu Kunliang, et al. Treatment of 2 cases of COVID-19 with umbilical cord mesenchymal stem cells. Chinese Journal of Cell and Stem Cell 2020; 10(4): 226-8.

24. Soler Rich R, Rius Tarruella J, Melgosa Camarero MT. Expanded Mesenchymal Stem Cells: a novel therapeutic approach of SARS-CoV-2 pneumonia (COVID-19). Concepts regarding a first case in Spain. *Medicina Clinica* 2020; **155**(7): 318-9.

25. Tang L, Jiang Y, Zhu M, et al. Clinical study using mesenchymal stem cells for the treatment of patients with severe COVID-19. *Frontiers of medicine* 2020; **14**(5): 664-73.

26. Tao J, Nie Y, Wu H, et al. Umbilical cord blood-derived mesenchymal stem cells in treating a critically ill COVID-19 patient. *Journal of Infection in Developing Countries* 2020; **14**(10): 1138-45.

27. Teixiera J, Atta MG, Noureddine L, et al. EXTRACORPOREAL MESENCHYMAL STROMAL CELL THERAPY (SBI-101) IN SEVERE COVID-19 COMPLICATED BY ACUTE KIDNEY INJURY. *Cytotherapy* 2021; **23**(5): S20.

28. Yilmaz R, Adas G, Cukurova Z, et al. Mesenchymal stem cells treatment in COVID-19 patient with multi-organ involvement. *Bratislavske lekarske listy* 2020; **121**(12): 847-52.

29. Zengin R, Beyaz O, Koc ES, et al. Mesenchymal stem cell treatment in a critically ill COVID-19 patient: a case report. *Stem Cell Investigation* 2020; **7**.

30. Zhang Q, Huang K, Lv J, et al. Case Report: Human Umbilical Cord Mesenchymal Stem Cells as a Therapeutic Intervention for a Critically Ill COVID-19 Patient. *Frontiers in Medicine* 2021; **8**.

31. Zhang Y, Ding J, Ren S, et al. Intravenous infusion of human umbilical cord Wharton's jelly-derived mesenchymal stem cells as a potential treatment for patients with COVID-19 pneumonia. *Stem Cell Research and Therapy* 2020; **11**(1).

32. Zhu Y, Zhu R, Liu K, et al. Human umbilical cord mesenchymal stem cells for adjuvant treatment of a critically ill COVID-19 patient: A case report. *Infection and Drug Resistance* 2020; **13**: 3295-300.

33. Zhu Y, Zhu RJ, Liu K, et al. Human Umbilical Cord Mesenchymal Stem Cells for Adjuvant Treatment of a Critically III COVID-19 Patient: A Case Report. *Infection and Drug Resistance* 2020; **13**: 3295-300.

34. Gu Liming, Li Tao, Zeng Yong, et al. Human Umbilical Cord Mesenchymal Stem Cells Treatment Combined with Anti-Virus and other Treatments for COVID-19. *Journal of Kunming Medical University* 2020; **41**(3): 96-100.

35. Li Peng, Yin Gang, Wang Rui, Guo Haiping. Clinical analysis of 5 patients with severe COVID-19 treated with certolizumab pegol and mesenchymal stem cells. *Journal of Shanxi Health Vocational College* 2020; **30**(5): 14-7.

36. Hashemian SM, Aliannejad R, Zarrabi M, et al. Mesenchymal stem cells derived from perinatal tissues for treatment of critically ill COVID-19-induced ARDS patients: a case series. *Stem cell research & therapy* 2021; **12**(1).

37. Averbuch D, De La Camara R, Corbacioglu S, et al. COVID-19 in Children Following Hematopoietic Cell Transplantation: A Multinational Study of the European Bone Marrow Transplantation Society (EBMT) and the Spanish Group of Hematopoietic Stem Cell Transplantation (GETH). *Blood* 2021; **138**: 2866.

38. Barkama R, Mayo A, Paz A, et al. Placenta-Derived Cell Therapy to Treat Patients With Respiratory Failure Due to Coronavirus Disease 2019. *Crit Care Explor* 2020; **2**(9): e0207.

39. Brown SA, Iancu-Rubin C, Aboelela A, et al. Mesenchymal stromal cell therapy for acute respiratory distress syndrome due to COVID-19. *Critical care medicine* 2021; **49**(1 SUPPL 1): 77‐.

40. Chen X, Shan Y, Wen Y, Sun J, Du H. Mesenchymal stem cell therapy in severe COVID-19: A retrospective study of short-term treatment efficacy and side effects. *Journal of Infection* 2020; **81**(4): 647-79.

41. Feng Y, Huang J, Wu J, et al. Safety and feasibility of umbilical cord mesenchymal stem cells in patients with COVID-19 pneumonia: A pilot study. *Cell Proliferation* 2020; **53**(12).

42. Guo Z, Chen Y, Luo X, He X, Zhang Y, Wang J. Administration of umbilical cord mesenchymal stem cells in patients with severe COVID-19 pneumonia. *Critical Care* 2020; **24**(1).

43. Kesari S, Kasper GC, Verkh L, et al. Mesenchymal stem cells in the treatment of severe COVID-19. *Translational Medicine Communications* 2021; **6**(1).

44. N OE, Pekkoc-Uyanik KC, Alpaydin N, Gulay GR, Simsek M. Clinical experience on umbilical cord mesenchymal stem cell treatment in 210 severe and critical COVID-19 cases in Turkey. *Stem cell reviews and reports* 2021.

45. Sadeghi B, Roshandel E, Pirsalehi A, et al. Conquering the cytokine storm in COVID-19-induced ARDS using placenta-derived decidua stromal cells. *Journal of Cellular and Molecular Medicine* 2021; **25**(22): 10554-64.

46. Sanchez-Guijo F, Garcia-Arranz M, Lopez-Parra M, et al. Adipose-derived mesenchymal stromal cells for the treatment of patients with severe SARS-CoV-2 pneumonia requiring mechanical ventilation. A proof of concept study. *Eclinicalmedicine* 2020; **25**.

47. Singh S, Chakravarty T, Chen P, et al. Allogeneic cardiosphere-derived cells (CAP-1002) in critically ill COVID-19 patients: compassionate-use case series. *Basic Research in Cardiology* 2020; **115**(4).

48. Yiğenoğlu TN, Başcı S, Şahin D, et al. Mesenchymal stem cell transfusion: Possible beneficial effects in COVID-19 patients. *Transfusion and Apheresis Science* 2021.

49. Zhou Juan, Hu Lingli, Tan Yingzheng, et al. Therapeutic Effect of Mesenchymal Stem Cells in the Treatment of Patients with Critical COVID-19. *Practical Journal of Cardiac Cerebral Pneumal and Vascular Disease* 2020; **28**(8): 14-8.

50. Feng G, Shi L, Huang T, et al. Human Umbilical Cord Mesenchymal Stromal Cell Treatment of Severe COVID-19 Patients: A 3-Month Follow-Up Study following Hospital Discharge. *Stem Cells and Development* 2021; **30**(15): 773-81.

51. Irct20200413047063N. Exosomes Derived from placental Mesenchymal Stem Cells as Treatment for Severe COVID-19: phase 1 & 2 Clinical Trials. [*https://trialsearchwhoint/Trial2aspx?TrialID=IRCT20200413047063N2*](https://trialsearchwhoint/Trial2aspx?TrialID=IRCT20200413047063N2) 2021.

52. Actrn. Cord Blood Therapy to prevent progression of COVID-19 related pneumonia. [*https://trialsearchwhoint/Trial2aspx?TrialID=ACTRN12620000478910*](https://trialsearchwhoint/Trial2aspx?TrialID=ACTRN12620000478910) 2020.

53. Actrn. The MEND (MEseNchymal coviD-19) Trial: a pilot study to investigate early efficacy of mesenchymal stem cells in adults with COVID-19. [*https://trialsearchwhoint/Trial2aspx?TrialID=ACTRN12620000612910*](https://trialsearchwhoint/Trial2aspx?TrialID=ACTRN12620000612910) 2020.

54. ChiCtr. Safety and efficacy of umbilical cord blood mononuclear cells conditioned medium in the treatment of severe and critically novel coronavirus pneumonia (COVID-19): a randomized controlled trial. [*https://trialsearchwhoint/Trial2aspx?TrialID=ChiCTR2000029569*](https://trialsearchwhoint/Trial2aspx?TrialID=ChiCTR2000029569) 2020; (研究者撤销).

55. ChiCtr. Safety and efficacy of umbilical cord blood mononuclear cells in the treatment of severe and critically novel coronavirus pneumonia(COVID-19): a randomized controlled clinical trial. [*https://trialsearchwhoint/Trial2aspx?TrialID=ChiCTR2000029572*](https://trialsearchwhoint/Trial2aspx?TrialID=ChiCTR2000029572) 2020; (研究者撤销).

56. ChiCtr. Safety and Efficacy Study of Allogeneic Human Dental Pulp Mesenchymal Stem Cells to Treat Severe novel coronavirus pneumonia (COVID-19) patients. [*https://trialsearchwhoint/Trial2aspx?TrialID=ChiCTR2000031319*](https://trialsearchwhoint/Trial2aspx?TrialID=ChiCTR2000031319) 2020.

57. ChiCtr. Severe novel coronavirus pneumonia (COVID-19) patients treated with ruxolitinib in combination with mesenchymal stem cells: a prospective, single blind, randomized controlled clinical trial. [*https://trialsearchwhoint/Trial2aspx?TrialID=ChiCTR2000029580*](https://trialsearchwhoint/Trial2aspx?TrialID=ChiCTR2000029580) 2020.

58. Ctri. A study to evaluate the effects of stem cells in patients with Acute Respiratory Distress syndrome caused by Pneumonia due to COVID-19. [*https://trialsearchwhoint/Trial2aspx?TrialID=CTRI/2020/10/028250*](https://trialsearchwhoint/Trial2aspx?TrialID=CTRI/2020/10/028250) 2020.

59. Ctri. Safety and efficacy evaluation of human Mesenchymal Stem Cells (MSCs) in patients with SARS-CoV-2 infection. [*https://trialsearchwhoint/Trial2aspx?TrialID=CTRI/2021/09/036645*](https://trialsearchwhoint/Trial2aspx?TrialID=CTRI/2021/09/036645) 2021.

60. Euctr ES. Clinical study to assess the safety and preliminary efficacy of HCR040, a drug based on mesenchymal stem cells, in patients with acute respiratory distress syndrome. (included patients COVID-19). [*https://trialsearchwhoint/Trial2aspx?TrialID=EUCTR2019-002688-89-ES*](https://trialsearchwhoint/Trial2aspx?TrialID=EUCTR2019-002688-89-ES) 2019.

61. Euctr ES. Clinical trial of administration of MSC to patients with respiratory distress type COVID-19. [*https://trialsearchwhoint/Trial2aspx?TrialID=EUCTR2020-001266-11-ES*](https://trialsearchwhoint/Trial2aspx?TrialID=EUCTR2020-001266-11-ES) 2020.

62. Euctr ES. Clinical Trial of allogeneic mesenchymal cells from umbilical cord tissue in patients with COVID-19. [*https://trialsearchwhoint/Trial2aspx?TrialID=EUCTR2020-001450-22-ES*](https://trialsearchwhoint/Trial2aspx?TrialID=EUCTR2020-001450-22-ES) 2020.

63. Euctr ES. Double-blind, randomized, controlled, clinical trial to assess the efficacy of allogenic mesenchymal stromal cells in patients with acute respiratory distress syndrome due to COVID-19. [*https://trialsearchwhoint/Trial2aspx?TrialID=EUCTR2020-002193-27-ES*](https://trialsearchwhoint/Trial2aspx?TrialID=EUCTR2020-002193-27-ES) 2020.

64. Euctr ES. Efficacy and safety evaluation of umbilical cord mesenchymal stem cells for the treatment of patients with respiratory failure due to coronavirus (COVID-19). [*https://trialsearchwhoint/Trial2aspx?TrialID=EUCTR2020-001505-22-ES*](https://trialsearchwhoint/Trial2aspx?TrialID=EUCTR2020-001505-22-ES) 2020.

65. Euctr ES. Study with stem cells from allogenic adipose tissue, in patients with coronavirus severe pneumonia. [*https://trialsearchwhoint/Trial2aspx?TrialID=EUCTR2020-001364-29-ES*](https://trialsearchwhoint/Trial2aspx?TrialID=EUCTR2020-001364-29-ES) 2020.

66. Euctr FR. Mesenchymal Stem Cell Therapy for SARS-CoV-2-related Acute Respiratory Distress Syndrome. [*https://trialsearchwhoint/Trial2aspx?TrialID=EUCTR2020-002772-12-FR*](https://trialsearchwhoint/Trial2aspx?TrialID=EUCTR2020-002772-12-FR) 2020.

67. Euctr IT. Clinical study on mesenchymal Cell Therapy for SARS-CoV-2 Pneumonia. [*https://trialsearchwhoint/Trial2aspx?TrialID=EUCTR2020-001577-70-IT*](https://trialsearchwhoint/Trial2aspx?TrialID=EUCTR2020-001577-70-IT) 2020.

68. Gorman E, Shankar-Hari M, Hopkins P, et al. Repair of Acute Respiratory Distress Syndrome by Stromal Cell Administration in COVID-19 (REALIST-COVID-19): a structured summary of a study protocol for a randomised, controlled trial. *Trials* 2020; **21**(1).

69. Irct20140528017891N. The effect of stem cell transplantation in the treatment of COVID-19. [*https://trialsearchwhoint/Trial2aspx?TrialID=IRCT20140528017891N8*](https://trialsearchwhoint/Trial2aspx?TrialID=IRCT20140528017891N8) 2020.

70. Irct20140911019125N. The effect of dental pulp mesenchymal stem cells in treatment of corona disease. [*https://trialsearchwhoint/Trial2aspx?TrialID=IRCT20140911019125N8*](https://trialsearchwhoint/Trial2aspx?TrialID=IRCT20140911019125N8) 2020.

71. Irct20160809029275N. stem cell therapy in Covid-19. [*https://trialsearchwhoint/Trial2aspx?TrialID=IRCT20160809029275N1*](https://trialsearchwhoint/Trial2aspx?TrialID=IRCT20160809029275N1) 2020.

72. Irct20180619040147N. Treatment of Covid-19 using menstrual blood stem cell secretome. [*https://trialsearchwhoint/Trial2aspx?TrialID=IRCT20180619040147N7*](https://trialsearchwhoint/Trial2aspx?TrialID=IRCT20180619040147N7) 2021.

73. Irct20190717044241N. Cell therapy in patients with COVID-19. [*https://trialsearchwhoint/Trial2aspx?TrialID=IRCT20190717044241N3*](https://trialsearchwhoint/Trial2aspx?TrialID=IRCT20190717044241N3) 2020.

74. Irct20200217046526N. Mesenchymal Stem Cell Therapy for Acute Respiratory Distress Syndrome in Coronavirus Infection. [*https://trialsearchwhoint/Trial2aspx?TrialID=IRCT20200217046526N2*](https://trialsearchwhoint/Trial2aspx?TrialID=IRCT20200217046526N2) 2020.

75. Irct20200325046860N. Mesenchymal Stem Cell therapy in COVID19. [*https://trialsearchwhoint/Trial2aspx?TrialID=IRCT20200325046860N2*](https://trialsearchwhoint/Trial2aspx?TrialID=IRCT20200325046860N2) 2020.

76. Irct20200413047063N. Placental Mesenchymal Stem Cell Therapy for Acute Respiratory Distress Syndrome in Coronavirus Infection: a Phase 1-2 Clinical Trial. [*https://trialsearchwhoint/Trial2aspx?TrialID=IRCT20200413047063N1*](https://trialsearchwhoint/Trial2aspx?TrialID=IRCT20200413047063N1) 2020.

77. Irct20200421047150N. Stem cell treatment for COVID-19. [*https://trialsearchwhoint/Trial2aspx?TrialID=IRCT20200421047150N1*](https://trialsearchwhoint/Trial2aspx?TrialID=IRCT20200421047150N1) 2020.

78. Irct20200426047206N. The efficacy and safety of mesenchymal stem cell transplantation in patients with COVID-19 pneumonia. [*https://trialsearchwhoint/Trial2aspx?TrialID=IRCT20200426047206N2*](https://trialsearchwhoint/Trial2aspx?TrialID=IRCT20200426047206N2) 2020.

79. Irct20200621047859N. The effects of mesenchymal stem cells in COVID-19. [*https://trialsearchwhoint/Trial2aspx?TrialID=IRCT20200621047859N4*](https://trialsearchwhoint/Trial2aspx?TrialID=IRCT20200621047859N4) 2021.

80. Irct20211012052743N. Stem cell therapy for COVID-19. [*https://trialsearchwhoint/Trial2aspx?TrialID=IRCT20211012052743N1*](https://trialsearchwhoint/Trial2aspx?TrialID=IRCT20211012052743N1) 2021.

81. Isrctn. To study the treatment of COVID-19 with severe viral pneumonia by using purified stem cell exosomes. [*https://trialsearchwhoint/Trial2aspx?TrialID=ISRCTN33578935*](https://trialsearchwhoint/Trial2aspx?TrialID=ISRCTN33578935) 2020.

82. Nct. MSC-based Therapy in COVID-19-associated Acute Respiratory Distress Syndrome. [*https://clinicaltrialsgov/show/NCT04525378*](https://clinicaltrialsgov/show/NCT04525378) 2020.

83. Nct. Inflammatory Signal Inhibitors for COVID-19 (MATIS). [*https://clinicaltrialsgov/show/NCT04581954*](https://clinicaltrialsgov/show/NCT04581954) 2020.

84. Nct. A Phase I/II Study of Universal Off-the-shelf NKG2D-ACE2 CAR-NK Cells for Therapy of COVID-19. [*https://clinicaltrialsgov/show/NCT04324996*](https://clinicaltrialsgov/show/NCT04324996) 2020.

85. Nct. RAPA-501-Allo Off-the-Shelf Therapy of COVID-19. [*https://clinicaltrialsgov/show/NCT04482699*](https://clinicaltrialsgov/show/NCT04482699) 2020.

86. Nct. Administration of Allogenic UC-MSCs as Adjuvant Therapy for Critically-Ill COVID-19 Patients. [*https://clinicaltrialsgov/show/NCT04457609*](https://clinicaltrialsgov/show/NCT04457609) 2020.

87. Nct. ASC Therapy for Patients With Severe Respiratory COVID-19. [*https://clinicaltrialsgov/show/NCT04341610*](https://clinicaltrialsgov/show/NCT04341610) 2020.

88. Nct. Autologous Adipose-derived Stem Cells (AdMSCs) for COVID-19. [*https://clinicaltrialsgov/show/NCT04428801*](https://clinicaltrialsgov/show/NCT04428801) 2020.

89. Nct. BAttLe Against COVID-19 Using MesenchYmal Stromal Cells. [*https://clinicaltrialsgov/show/NCT04348461*](https://clinicaltrialsgov/show/NCT04348461) 2020.

90. Nct. Bone Marrow-Derived Mesenchymal Stem Cell Treatment for Severe Patients With Coronavirus Disease 2019 (COVID-19). [*https://clinicaltrialsgov/show/NCT04346368*](https://clinicaltrialsgov/show/NCT04346368) 2020.

91. Nct. Cell Therapy Using Umbilical Cord-derived Mesenchymal Stromal Cells in SARS-CoV-2-related ARDS. [*https://clinicaltrialsgov/show/NCT04333368*](https://clinicaltrialsgov/show/NCT04333368) 2020.

92. Nct. Clinical Research of Human Mesenchymal Stem Cells in the Treatment of COVID-19 Pneumonia. [*https://clinicaltrialsgov/show/NCT04339660*](https://clinicaltrialsgov/show/NCT04339660) 2020.

93. Nct. Clinical Trial to Assess the Efficacy of MSC in Patients With ARDS Due to COVID-19. [*https://clinicaltrialsgov/show/NCT04615429*](https://clinicaltrialsgov/show/NCT04615429) 2020.

94. Nct. Clinical Trial to Assess the Safety and Efficacy of Intravenous Administration of Allogeneic Adult Mesenchymal Stem Cells of Expanded Adipose Tissue in Patients With Severe Pneumonia Due to COVID-19. [*https://clinicaltrialsgov/show/NCT04366323*](https://clinicaltrialsgov/show/NCT04366323) 2020.

95. Nct. Cord Blood-Derived Mesenchymal Stem Cells for the Treatment of COVID-19 Related Acute Respiratory Distress Syndrome. [*https://clinicaltrialsgov/show/NCT04565665*](https://clinicaltrialsgov/show/NCT04565665) 2020.

96. Nct. Efficacy and Safety Evaluation of Mesenchymal Stem Cells for the Treatment of Patients With Respiratory Distress Due to COVID-19. [*https://clinicaltrialsgov/show/NCT04390139*](https://clinicaltrialsgov/show/NCT04390139) 2020.

97. Nct. Efficacy of Infusions of MSC From Wharton Jelly in the SARS-Cov-2 (COVID-19) Related Acute Respiratory Distress Syndrome. [*https://clinicaltrialsgov/show/NCT04625738*](https://clinicaltrialsgov/show/NCT04625738) 2020.

98. Nct. Efficacy of Intravenous Infusions of Stem Cells in the Treatment of COVID-19 Patients. [*https://clinicaltrialsgov/show/NCT04437823*](https://clinicaltrialsgov/show/NCT04437823) 2020.

99. Nct. Expanded Access Protocol on Bone Marrow Mesenchymal Stem Cell Derived Extracellular Vesicle Infusion Treatment for Patients With COVID-19 Associated ARDS. [*https://clinicaltrialsgov/show/NCT04657458*](https://clinicaltrialsgov/show/NCT04657458) 2020.

100. Nct. hCT-MSCs for COVID19 ARDS. [*https://clinicaltrialsgov/show/NCT04399889*](https://clinicaltrialsgov/show/NCT04399889) 2020.

101. Nct. The MEseNchymal coviD-19 Trial: a Pilot Study to Investigate Early Efficacy of MSCs in Adults With COVID-19. [*https://clinicaltrialsgov/show/NCT04537351*](https://clinicaltrialsgov/show/NCT04537351) 2020.

102. Nct. Mesenchymal Stem Cell Infusion for COVID-19 Infection. [*https://clinicaltrialsgov/show/NCT04444271*](https://clinicaltrialsgov/show/NCT04444271) 2020.

103. Nct. Mesenchymal Stem Cells for the Treatment of COVID-19. [*https://clinicaltrialsgov/show/NCT04573270*](https://clinicaltrialsgov/show/NCT04573270) 2020.

104. Nct. Mesenchymal Stem Cells in Patients Diagnosed With COVID-19. [*https://clinicaltrialsgov/show/NCT04611256*](https://clinicaltrialsgov/show/NCT04611256) 2020.

105. Nct. MRG-001 as an Immunoregulatory and Regenerative Therapy for COVID-19 Patients. [*https://clinicaltrialsgov/show/NCT04646603*](https://clinicaltrialsgov/show/NCT04646603) 2020.

106. Nct. Multiple Dosing of Mesenchymal Stromal Cells in Patients With ARDS (COVID-19). [*https://clinicaltrialsgov/show/NCT04466098*](https://clinicaltrialsgov/show/NCT04466098) 2020.

107. Nct. Open-label Multicenter Study to Evaluate the Efficacy of PLX-PAD for the Treatment of COVID-19. [*https://clinicaltrialsgov/show/NCT04614025*](https://clinicaltrialsgov/show/NCT04614025) 2020.

108. Nct. Regenerative Medicine for COVID-19 and Flu-Elicited ARDS Using Longeveron Mesenchymal Stem Cells (LMSCs) (RECOVER). [*https://clinicaltrialsgov/show/NCT04629105*](https://clinicaltrialsgov/show/NCT04629105) 2020.

109. Nct. Safety and Effectiveness of Mesenchymal Stem Cells in the Treatment of Pneumonia of Coronavirus Disease 2019. [*https://clinicaltrialsgov/show/NCT04371601*](https://clinicaltrialsgov/show/NCT04371601) 2020.

110. Nct. Safety and Efficacy of Intravenous Wharton's Jelly Derived Mesenchymal Stem Cells in Acute Respiratory Distress Syndrome Due to COVID 19. [*https://clinicaltrialsgov/show/NCT04390152*](https://clinicaltrialsgov/show/NCT04390152) 2020.

111. Nct. Safety and Efficacy of Mesenchymal Stem Cells in the Management of Severe COVID-19 Pneumonia. [*https://clinicaltrialsgov/show/NCT04429763*](https://clinicaltrialsgov/show/NCT04429763) 2020.

112. Nct. Safety and Efficacy Study of Allogeneic Human Dental Pulp Mesenchymal Stem Cells to Treat Severe COVID-19 Patients. [*https://clinicaltrialsgov/show/NCT04336254*](https://clinicaltrialsgov/show/NCT04336254) 2020.

113. Nct. Safety and Efficiency of Method of Exosome Inhalation in COVID-19 Associated Pneumonia. [*https://clinicaltrialsgov/show/NCT04602442*](https://clinicaltrialsgov/show/NCT04602442) 2020.

114. Nct. Stem Cell Educator Therapy Treat the Viral Inflammation in COVID-19. [*https://clinicaltrialsgov/show/NCT04299152*](https://clinicaltrialsgov/show/NCT04299152) 2020.

115. Nct. A Study of Cell Therapy in COVID-19 Subjects With Acute Kidney Injury Who Are Receiving Renal Replacement Therapy. [*https://clinicaltrialsgov/show/NCT04445220*](https://clinicaltrialsgov/show/NCT04445220) 2020.

116. Nct. Treatment of Severe COVID-19 Pneumonia With Allogeneic Mesenchymal Stromal Cells (COVID_MSV). [*https://clinicaltrialsgov/show/NCT04361942*](https://clinicaltrialsgov/show/NCT04361942) 2020.

117. Nct. Treatment With Mesenchymal Stem Cells for Severe Corona Virus Disease 2019(COVID-19). [*https://clinicaltrialsgov/show/NCT04288102*](https://clinicaltrialsgov/show/NCT04288102)

[*https://pubmedncbinlmnihgov/33568628/?dopt=Abstract*](https://pubmedncbinlmnihgov/33568628/?dopt=Abstract) 2020.

118. Nct. Umbilical Cord Lining Stem Cells (ULSC) in Patients With COVID-19 ARDS. [*https://clinicaltrialsgov/show/NCT04494386*](https://clinicaltrialsgov/show/NCT04494386) 2020.

119. Nct. Umbilical Cord Tissue (UC) Derived Mesenchymal Stem Cells (MSCs) Versus Placebo to Treat Acute Pulmonary Inflammation Due to COVID-19. [*https://clinicaltrialsgov/show/NCT04490486*](https://clinicaltrialsgov/show/NCT04490486) 2020.

120. Nct. Cellular Immuno-Therapy for COVID-19 ARDS Randomized Clinical Trial. [*https://clinicaltrialsgov/show/NCT04865107*](https://clinicaltrialsgov/show/NCT04865107) 2021.

121. Nct. Off-the-shelf NK Cells (KDS-1000) as Immunotherapy for COVID-19. [*https://clinicaltrialsgov/show/NCT04797975*](https://clinicaltrialsgov/show/NCT04797975) 2021.

122. Nct. Allogenic UCMSCs as Adjuvant Therapy for Severe COVID-19 Patients. [*https://clinicaltrialsgov/show/NCT05132972*](https://clinicaltrialsgov/show/NCT05132972) 2021.

123. Nct. Bone Marrow Mesenchymal Stem Cell Derived Extracellular Vesicles Infusion Treatment for Mild-to-Moderate COVID-19: a Phase II Clinical Trial. [*https://clinicaltrialsgov/show/NCT05125562*](https://clinicaltrialsgov/show/NCT05125562) 2021.

124. Nct. Clinical Study for Subjects With COVID-19 Using Allogeneic Adipose Tissue-Derived Mesenchymal Stem Cells. [*https://clinicaltrialsgov/show/NCT05017298*](https://clinicaltrialsgov/show/NCT05017298) 2021.

125. Nct. ExoFlo™ Infusion for Post-Acute COVID-19 and Chronic Post-COVID-19 Syndrome. [*https://clinicaltrialsgov/show/NCT05116761*](https://clinicaltrialsgov/show/NCT05116761) 2021.

126. Nct. Menstrual Blood Stem Cells in Severe Covid-19. [*https://clinicaltrialsgov/show/NCT05019287*](https://clinicaltrialsgov/show/NCT05019287) 2021.

127. Nct. Mesenchymal Stem Cell Secretome In Severe Cases of COVID-19. [*https://clinicaltrialsgov/show/NCT05122234*](https://clinicaltrialsgov/show/NCT05122234) 2021.

128. Nct. Mesenchymal Stem Cells Therapy in Patients With COVID-19 Pneumonia. [*https://clinicaltrialsgov/show/NCT04713878*](https://clinicaltrialsgov/show/NCT04713878) 2021.

129. Nct. Randomized Double-Blind Phase 2 Study of Allogeneic HB-adMSCs for the Treatment of Chronic Post-COVID-19 Syndrome. [*https://clinicaltrialsgov/show/NCT05126563*](https://clinicaltrialsgov/show/NCT05126563) 2021.

130. Nct. Study of Allogeneic Adipose-Derived Mesenchymal Stem Cells for Non-COVID Acute Respiratory Distress Syndrome. [*https://clinicaltrialsgov/show/NCT04909879*](https://clinicaltrialsgov/show/NCT04909879) 2021.

131. Nct. Study of Allogeneic Adipose-Derived Mesenchymal Stem Cells for Treatment of COVID-19 Acute Respiratory Distress. [*https://clinicaltrialsgov/show/NCT04905836*](https://clinicaltrialsgov/show/NCT04905836) 2021.

132. Nct. Study of Allogeneic Adipose-Derived Mesenchymal Stem Cells to Treat Post COVID-19 "Long Haul" Pulmonary Compromise. [*https://clinicaltrialsgov/show/NCT04992247*](https://clinicaltrialsgov/show/NCT04992247) 2021.

133. Nct. Study of Intravenous Administration of Allogeneic Adipose-Derived Mesenchymal Stem Cells for COVID-19-Induced Acute Respiratory Distress. [*https://clinicaltrialsgov/show/NCT04728698*](https://clinicaltrialsgov/show/NCT04728698) 2021.

134. Nct. Treatment of Respiratory Complications Associated With COVID-19 Using Umbilical Cord Mesenchymal Stromal Cells. [*https://clinicaltrialsgov/show/NCT04869397*](https://clinicaltrialsgov/show/NCT04869397) 2021.

135. Nct. Treatment of Severe COVID-19 Patients Using Secretome of Hypoxia-Mesenchymal Stem Cells in Indonesia. [*https://clinicaltrialsgov/show/NCT04753476*](https://clinicaltrialsgov/show/NCT04753476) 2021.

136. Payares-Herrera C, Martinez-Munoz ME, Vallhonrat IL, et al. Double-blind, randomized, controlled, trial to assess the efficacy of allogenic mesenchymal stromal cells in patients with acute respiratory distress syndrome due to COVID-19 (COVID-AT): a structured summary of a study protocol for a randomised controlled trial. *Trials* 2021; **22**(1).

137. Rpcec. Stem cells in patients with lung lesions (COVID-19). [*https://trialsearchwhoint/Trial2aspx?TrialID=RPCEC00000322*](https://trialsearchwhoint/Trial2aspx?TrialID=RPCEC00000322) 2020.

138. Rynda A, Hancharou A, Antonevich N, et al. Safety of pooled mesenchimal stem cells in patients with severe COVID-19 pneumonia. *Allergy: European Journal of Allergy and Clinical Immunology* 2021; **76**(SUPPL 110): 208-9.

139. trt RBR. Clinical study to assess safety and efficacy related to the use of donor stem cells in the treatment of COVID-19. [*https://trialsearchwhoint/Trial2aspx?TrialID=RBR-65trt53*](https://trialsearchwhoint/Trial2aspx?TrialID=RBR-65trt53) 2021.

140. Agarwal S, June CH. Harnessing CAR T-cell Insights to Develop Treatments for Hyperinflammatory Responses in Patients with COVID-19. *Cancer Discov* 2020; **10**(6): 775-8.

141. Agrawal N, Singh R, Sharma SK, et al. Outcomes of COVID-19 in Hematopoietic Stem Cell Transplant Recipients: Multicenter Retrospective Analysis. *Indian Journal of Hematology and Blood Transfusion* 2021.

142. Ali G, Altareb M, Chaudhri N. Outcome of Haploidentical Hematopoietic Stem Cell Transplantation with a Donor and Recipient with SARS-CoV-2 Infection. *Clinical Lymphoma Myeloma & Leukemia* 2021; **21**: S325-S6.

143. Antonio PM, Cristina FP, Alejandro MQ, et al. PRELIMINARY RESULTS OF THE PHASE I/II RELEASE CLINICAL TRIAL WITH ADOPTIVE CELL THERAPY WITH MEMORY T-LYMPHOCYTES FOR PATIENTS WITH MODERATE/SEVERE COVID19 DISEASE. *Haematologica* 2021; **106**(10): 128-.

144. Basar R, Uprety N, Ensley E, et al. Generation of glucocorticoid-resistant SARS-CoV-2 T cells for adoptive cell therapy. *Cell Reports* 2021; **36**(3).

145. Nishiga M, Wu JC. Macrophages: Potential Therapeutic Target of Myocardial Injury in COVID-19. *Circulation Research* 2021: 47-9.

146. Ortiz de Landazuri I, Egri N, Muñoz-Sánchez G, et al. Manufacturing and Management of CAR T-Cell Therapy in “COVID-19’s Time”: Central Versus Point of Care Proposals. *Frontiers in Immunology* 2020; **11**.

147. Pera M. Message from the Editor: Stem Cell Science in the Time of COVID-19. *Stem Cell Reports* 2020; **14**(4): 529.

148. Pérez-Martínez A, Ferreras C, Mora-Rillo M, et al. A Phase i Dose-Escalation Single Center Study to Evaluate the Safety of Allogenic Memory T Cells Containing SARS-COV-2 Specific Lymphocytes As Adoptive Therapy in COVID19. *Bone Marrow Transplantation* 2021; **56**: 49-50.

149. Perez-Martinez A, Ferreras C, Mora-Rillo M, et al. A PHASE I/II DOSE-ESCALATION SINGLE CENTER STUDY TO EVALUATE THE SAFETY OF INFUSION OF MEMORY T CELLS AS ADOPTIVE THERAPY IN CORONAVIRUS PNEUMONIA AND /OR LYMPHOPENIA (RELEASE). *Cytotherapy* 2021; **23**(5): S29.

150. Pérez-Martínez A, Martín-Quirós A, Ferreras C, et al. A Phase I/II Dose-Escalation Multi-Center Study to Evaluate the Safety of Infusion of Natural Killer Cells or Memory T Cells As Adoptive Therapy in Coronavirus Pneumonia and/or Lymphopenia: (RELEASE NCT04578210). *Blood* 2021; **138**: 1765.

151. Piñana JL, López-Corral L, Martino R, et al. SARS-CoV-2-reactive antibody detection after SARS-CoV-2 vaccination in hematopoietic stem cell transplant recipients: Prospective survey from the Spanish Hematopoietic Stem Cell Transplantation and Cell Therapy Group. *American Journal of Hematology* 2022; **97**(1): 30-42.

152. Pineda-Terreros B, Acosta-Maldonado BL, Valero-Saldaña LM, et al. SARS-COV-2 Infection in A Hematopoietic Stem Cell Transplant Center: A Single Center Experience in Mexico. *Bone Marrow Transplantation* 2021; **56**: 268.

153. Qatawneh M, Aljazazi M, Altarawneh M, et al. Hematopoietic Stem Cell Transplantation During the Era of COVID-19 in Queen Rania Children's Hospital. *Mater Sociomed* 2021; **33**(2): 131-7.

154. Rajendra A, Gokarn A, Mirgh S, et al. SARS-CoV2 Infection in Hematopoietic Stem Cell Transplant recipients: A Case Series from a Tertiary Cancer Centre in India. *Indian Journal of Hematology and Blood Transfusion* 2021; **37**(4): 699-701.

155. Ram R, Hagin D, Freund T, et al. Safety and efficacy of the BNT162B2 MRNA covid-19 vaccine in patients after allogeneic HCT and CD19-based CAR-T therapy - A single center prospective cohort study. *HemaSphere* 2021; **5**(SUPPL 2): 101.

156. Ratajczak MZ, Kucia M. Stem Cells as Potential Therapeutics and Targets for Infection by COVID19 - Special Issue on COVID19 in Stem Cell Reviews and Reports. *Stem Cell Rev Rep* 2021; **17**(1): 1-3.

157. Rpcec. ATENEA-Co-300 study. [*https://trialsearchwhoint/Trial2aspx?TrialID=RPCEC00000317*](https://trialsearchwhoint/Trial2aspx?TrialID=RPCEC00000317) 2020.

158. Salehi MS, Pandamooz S, Jurek B. Epidermal Neural Crest Stem Cells as a Perspective for COVID-19 Treatment. *Stem Cell Reviews and Reports* 2021; **17**(1): 291-2.

159. Sbragia E, Mariottini A, Capobianco M, et al. COVID-19 in patients with aggressive MS treated with aHSCT: A multi-center study. *Multiple Sclerosis Journal* 2021; **27**(2 SUPPL): 630-1.

160. Senegaglia AC, Rebelatto CLK, Franck CL, et al. USE OF TOCILIZUMAB AND MESENCHYMAL STROMAL CELLS IN THE TREATMENT OF SEVERE COVID-19 - A CASE REPORT. *Cytotherapy* 2021; **23**(4): 38-9.

161. Shah GL, DeWolf S, Lee YJ, et al. Favorable outcomes of COVID-19 in recipients of hematopoietic cell transplantation. *Journal of Clinical Investigation* 2020; **130**(12): 6656-67.

162. Sharma A, Bhatt NS, St Martin A, et al. Clinical characteristics and outcomes of COVID-19 in haematopoietic stem-cell transplantation recipients: an observational cohort study. *The Lancet Haematology* 2021; **8**(3): e185-e93.

163. Shetty AK, Shetty PA, Zanirati G, Jin K. Further validation of the efficacy of mesenchymal stem cell infusions for reducing mortality in COVID-19 patients with ARDS. *npj Regenerative Medicine* 2021; **6**(1).

164. Sivapalan R, Liu J, Chakraborty K, et al. Virus Induced Lymphocytes (VIL) as a novel viral antigen-specific T cell therapy for COVID-19 and potential future pandemics. *Scientific reports* 2021; **11**(1): 15295.

165. Stanley K, Hanmod S, Simpson RJ, Katsanis E. Haploidentical hematopoietic cell transplantation is even more advantageous during the COVID-19 pandemic. *Pediatric Transplantation* 2021; **25**(3).

166. Tufan AC. Mesenchymal Stem Cells as a Treatment Strategy for Coronavirus Disease 2019 (COVID-19): Need for Authority Regulations and Clinical Guide-lines. *Current Stem Cell Research & Therapy* 2021; **16**(4): 465-80.

167. Vaiasicca S, Corradetti B. Stem cells from the amniotic fluid: a promising tool to face the cytokine storm associated to SARS-CoV-2 infections. *Faseb Journal* 2021; **35**.

168. Vasileiou S, Kuvalekar M, Workineh A, et al. Using allogeneic, off-the-shelf, SARS-COV-2-specific t cells to treat high risk patients with covid-19. *Blood* 2020; **136**(SUPPL 1): 5‐.

169. Vishnevetsky A, Hawkes C, Lechner-Scott J, Giovannoni G, Levy M, Pohl D. B cell therapy and the use of RNA-based COVID-19 vaccines. *Multiple Sclerosis and Related Disorders* 2021; **49**.

170. Zuo W, Zhao X. Natural killer cells play an important role in virus infection control: Antiviral mechanism, subset expansion and clinical application. *Clinical Immunology* 2021; **227**.

171. Fan Xiaole, Wang Shuiping, Wu Bingyi, et al. Discussion and prospect of infusion of NK cells in the treatment of SARS-CoV-2 infection. *Chinese Journal of Cellular and Molecular Immunology* 2020; **36**(5): 457-61.

172. Hou Xiaojn, Yang Min. Advances in the potential of natural killer cell-based immunotherapy for treatment of COVID-19. *Progress in Microbiology and Immunology* 2021; **49**(1).

173. Saleh M, Vaezi AA, Aliannejad R, et al. Cell therapy in patients with COVID-19 using Wharton’s jelly mesenchymal stem cells: a phase 1 clinical trial. *Stem Cell Research and Therapy* 2021; **12**(1).

174. Wu J, Zhou X, Tan Y, et al. Phase 1 trial for treatment of COVID-19 patients with pulmonary fibrosis using hESC-IMRCs. *Cell Proliferation* 2020; **53**(12).

175. Andrade HD, Kondo AT, Kerbauy L, et al. Treatment of COVID-19 Patients: Insights into the Use of Bone Marrow-Derived Mesenchymal Stem Cells. *Blood* 2021; **138**: 4302.

176. Pérez-Martínez A, Mora-Rillo M, Ferreras C, et al. Phase I dose-escalation single centre clinical trial to evaluate the safety of infusion of memory T cells as adoptive therapy in COVID-19 (RELEASE). *EClinicalMedicine* 2021; **39**.
