# Supplementary material for: Safety and efficiency of stem cell therapy for COVID-19: a systematic review and meta-analysis
Source: Glob Health Res Policy. 2022 Jun 23;7:19. doi: 10.1186/s41256-022-00251-5 (PMC9217728; doi:10.1186/s41256-022-00251-5)
Supplement: Supplementary file 3 — Additional file 3. Quality Assessment of 10 RCTs. 10 RCTs included in the systematic review were assessed for literature quality using the Risk-of-bias Tool 1.0. a: Risk of bias graph: authors' judgements’ about each risk of bias item presented as percentages. b: Risk of bias summary: authors' judgements about each risk of bias item. [file 41256_2022_251_MOESM3_ESM.pdf]

Figure S2. Quality Assesment of 10 RCTs by Risk-of-bias Tool 1.0

a

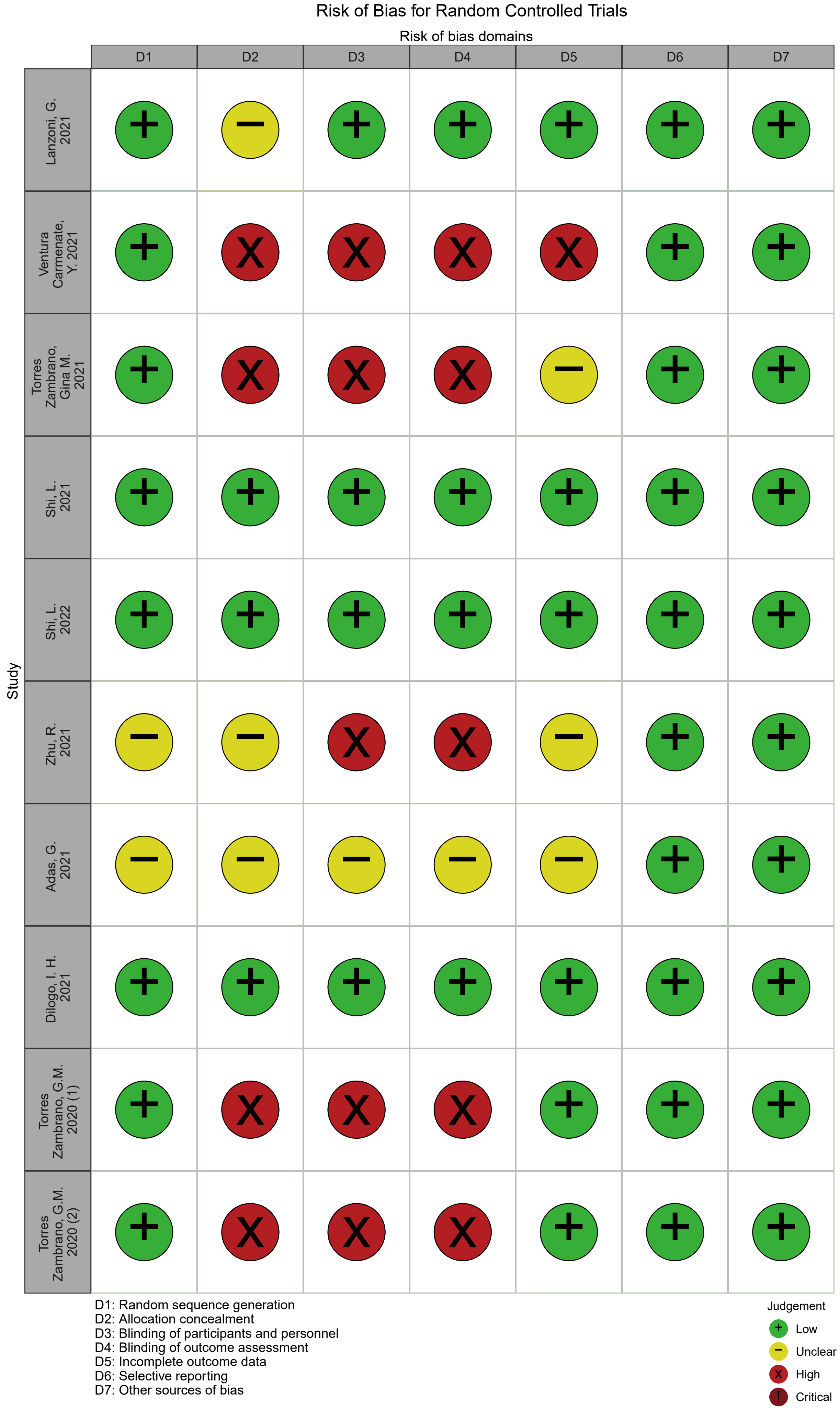

b

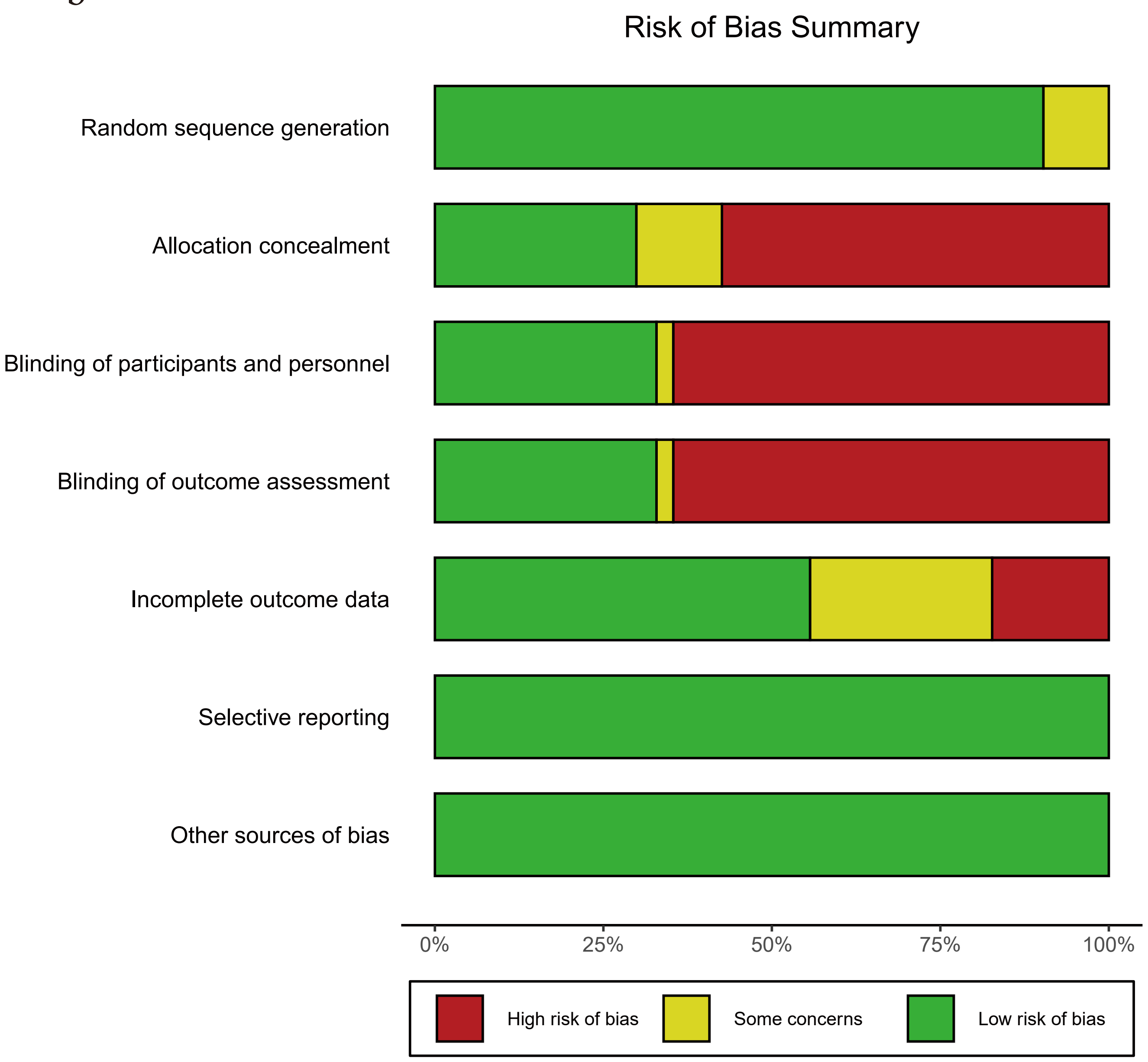

a: Risk of bias graph: authors' judgements about each risk of bias item presented as percentages
b: Risk of bias summary: authors' judgements about each risk of bias item

Torres Zambrano, G.M. 2020 (1) Renal Involvement in Patients with COVID-19 Pneumonia and Outcomes After Stem Cell Nebulization;
Torres Zambrano, G.M. 2020 (2) Features and outcomes of secondary sepsis and urinary tract infections in COVID-19 patients treated with stem cell nebulization.
