# Supplementary material for: Safety and efficiency of stem cell therapy for COVID-19: a systematic review and meta-analysis
Source: Glob Health Res Policy. 2022 Jun 23;7:19. doi: 10.1186/s41256-022-00251-5 (PMC9217728; doi:10.1186/s41256-022-00251-5)
Supplement: Supplementary file 4 — Additional file 4. Quality Assessment of 7 NRCTs. 7 NRCTs included in the systematic review were assessed for literature quality using the MINORS instrument. [file 41256_2022_251_MOESM4_ESM.docx]

**Additional file 4. Quality Assessment of 7 NRCTs**

| **Quality assessment for nonrandomized trials** | **Studies** | | | | | | |
| --- | --- | --- | --- | --- | --- | --- | --- |
|  | **Leng, Z.2020** | **Meng, F.2020** | **Shu, L.2020** | **Wei, F.2021** | **Häberle, H.2021** | **Xu, X.2021** | **O.Ercelen, N.2020** |
| A clearly stated aim | 2 | 2 | 2 | 2 | 2 | 2 | 2 |
| Inclusion of consecutive patients | 2 | 2 | 2 | 2 | 2 | 2 | 0 |
| Prospective collection of data | 2 | 2 | 2 | 2 | 0 | 2 | 0 |
| Endpoints appropriate to the aim of the study | 2 | 2 | 2 | 2 | 2 | 2 | 2 |
| Unbiased assessment of the study endpoint | 1 | 0 | 1 | 1 | 1 | 0 | 0 |
| Follow-up period appropriate to the aim of the study | 2 | 2 | 2 | 2 | 2 | 2 | 2 |
| Loss to follow up less than 5% | 2 | 2 | 2 | 2 | 2 | 2 | 2 |
| Prospective calculation of the study size | 0 | 0 | 0 | 0 | 0 | 0 | 0 |
| An adequate control group | 2 | 2 | 2 | 1 | 2 | 2 | 2 |
| Contemporary groups | 2 | 2 | 1 | 0 | 2 | 2 | 2 |
| Baseline equivalence of groups | 1 | 2 | 2 | 1 | 2 | 2 | 1 |
| Adequate statistical analyses | 1 | 1 | 1 | 2 | 1 | 2 | 0 |
| Total score | 19 | 19 | 19 | 17 | 18 | 20 | 13 |
| Average | 17.86 | | | | | | |
