# Supplementary material for: Safety and efficiency of stem cell therapy for COVID-19: a systematic review and meta-analysis
Source: Glob Health Res Policy. 2022 Jun 23;7:19. doi: 10.1186/s41256-022-00251-5 (PMC9217728; doi:10.1186/s41256-022-00251-5)
Supplement: Supplementary file 7 — Additional file 7. Serious Adverse Events. All serious adverse events reported in involved studies between experimental and control groups. [file 41256_2022_251_MOESM7_ESM.docx]

**Additional file 7. Serious Adverse Events**

| **SAEs** | **Experimental** | **Control** | **Total** | **Articles** |
| --- | --- | --- | --- | --- |
| Sepsis | 5 | 15 | 20 | (1) |
| Deaths with no cause reported | 6 | 14 | 20 | (1, 2) |
| Anemia | 10 | 8 | 18 | (1) |
| Disease progression | 5 | 9 | 14 | (1) |
| Acute Respiratory Distress Syndrome | 4 | 7 | 11 | (1, 3) |
| Multiple organ failure | 3 | 8 | 11 | (1, 3) |
| Metabolic alkalosis | 4 | 6 | 10 | (1) |
| Anoxia | 3 | 4 | 7 | (1) |
| Shock | 3 | 4 | 7 | (2, 3) |
| Cardiac arrest | 1 | 3 | 4 | (2) |
| Gastrointestinal bleeding | 2 | 1 | 3 | (2, 3) |
| Pneumothorax | 1 | 1 | 2 | (2, 4) |
| Abnormal blood clotting function |  | 1 | 1 | (3) |
| Hyperkalemia |  | 1 | 1 | (2) |
| Metabolic acidosis |  | 1 | 1 | (2) |
| Severe respiratory acidosis |  | 1 | 1 | (2) |
| Circulatory failure | 1 |  | 1 | (4) |
| Room punch | 1 |  | 1 | (2) |
| Respiratory failure | 1 |  | 1 | (5) |
| Dyspnea | 1 |  | 1 | (3) |
| Colon perforation | 1 |  | 1 | (2) |
| Psychosis |  | 1 |  | (3) |

1. Ventura-Carmenate Y, Alkaabi FM, Castillo-Aleman YM, Villegas-Valverde CA, Ahmed YM, Sanna P, et al. Safety and efficacy of autologous non-hematopoietic enriched stem cell nebulization in COVID-19 patients: a randomized clinical trial, Abu Dhabi 2020. Transl Med Commun. 2021;6(1):25.

2. Lanzoni G, Linetsky E, Correa D, Messinger Cayetano S, Alvarez RA, Kouroupis D, et al. Umbilical cord mesenchymal stem cells for COVID-19 acute respiratory distress syndrome: a double-blind, phase 1/2a, randomized controlled trial. Stem cells translational medicine. 2021;10(5):660‐73.

3. Xu X, Jiang W, Chen L, Xu Z, Zhang Q, Zhu M, et al. Evaluation of the safety and efficacy of using human menstrual blood-derived mesenchymal stromal cells in treating severe and critically ill COVID-19 patients: An exploratory clinical trial. Clin Transl Med. 2021;11(2):e297.

4. Shi L, Huang H, Lu X, Yan X, Jiang X, Xu R, et al. Effect of human umbilical cord-derived mesenchymal stem cells on lung damage in severe COVID-19 patients: a randomized, double-blind, placebo-controlled phase 2 trial. Signal Transduct Target Ther. 2021;6(1):58.

5. Wei F, Kong D, Li T, Li A, Tan Y, Fang J, et al. Efficacy and safety of umbilical cord mesenchymal stem cells for the treatment of patients with COVID-19. Clinics (Sao Paulo). 2021;76:e2604.
