# Supplementary material for: Safety and efficiency of stem cell therapy for COVID-19: a systematic review and meta-analysis
Source: Glob Health Res Policy. 2022 Jun 23;7:19. doi: 10.1186/s41256-022-00251-5 (PMC9217728; doi:10.1186/s41256-022-00251-5)
Supplement: Supplementary file 8 — Additional file 8. Results of Meta-analysis of Laboratory parameters. Results of Meta-analysis of WBC, neutrophiles, lymphocytes, platelets, CRP, IL-6, TNF-α, D-dimer, fibrinogen and ferritin in day 0–4 or day 5–8. a. Forest plot of WBC (day5–8): Std. Mean Difference (95% CI) and pooled estimates. b. Forest plot of neutrophils (day5–8): Std. Mean Difference (95% CI) and pooled estimates. c. Forest plot of lymphocytes (day5–8): Std. Mean Difference (95% CI) and pooled estimates. d. Forest plot of PLT (day0–4): Std. Mean Difference (95% CI) and pooled estimates. e. Forest plot of PLT (day5-8): Std. Mean Difference (95% CI) and pooled estimates. f. Forest plot of CRP (day0–4): Std. Mean Difference (95% CI) and pooled estimates. g. Forest plot of CRP (day5–8): Std. Mean Difference (95% CI) and pooled estimates. h. Forest plot of IL-6 (day5–8): Std. Mean Difference (95% CI) and pooled estimates. I. Forest plot of TNF-α (day5–8): Std. Mean Difference (95% CI) and pooled estimates. j. Forest plot of D-dimer (day0–4): Std. Mean Difference (95% CI) and pooled estimates. k. Forest plot of D-dimer (day5–8): Std. Mean Difference (95% CI) and pooled estimates. l. Forest plot of fibrinogen (day0–4): Std. Mean Difference (95% CI) and pooled estimates. m. Forest plot of fibrinogen (day5–8): Std. Mean Difference (95% CI) and pooled estimates. n. Forest plot of ferritin (day0–4): Std. Mean Difference (95% CI) and pooled estimates. o. Forest plot of ferritin (day5–8): Std. Mean Difference (95% CI) and pooled estimates. [file 41256_2022_251_MOESM8_ESM.pdf]

Additional file 8. Results of Meta-analysis of Laboratory parameters.

Study	Experimental Mean	Experimental SD	Experimental Total	Control Mean	Control SD	Control Total	Weight	Std. Mean Difference IV, Random, 95% CI
Lanzoni, G. 2021	13.43	4.6000	9	15.53	5.6000	9	14.0%	−0.39 [−1.33, 0.54]
Ventura−Carmenate, Y. 2021	7.60	17.0900	51	10.99	23.4800	35	66.1%	−0.17 [−0.60, 0.26]
Wei, F. 2021	7.20	2.6000	12	6.78	1.6600	13	19.8%	0.19 [−0.60, 0.97]
**Total (95% CI)**			**72**			**57**	**100.0%**	**−0.13 [−0.48, 0.22]**

Heterogeneity: $Tau^2 = 0$; $Chi^2 = 0.96$, df = 2 (P = 0.62); $I^2 = 0\%$
Test for overall effect: Z = −0.72 (P = 0.47)

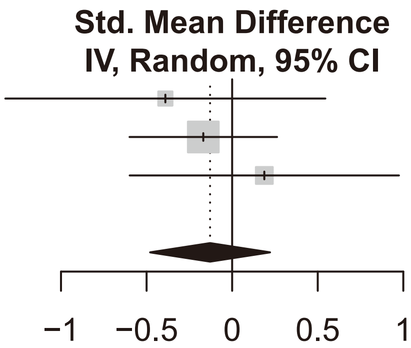

a. Forest plot of WBC (day5-8): Std. Mean Difference (95% CI) and pooled estimates.

Study	Experimental Mean	SD	Total	Control Mean	SD	Total	Weight	Std. Mean Difference IV, Random, 95% CI
Lanzoni, G. 2021	9.74	4.2700	8	13.40	5.9500	9	12.0%	−0.66 [−1.65, 0.32]
Ventura−Carmenate, Y. 2021	4.30	15.6700	51	7.27	23.4100	35	62.8%	−0.15 [−0.58, 0.28]
Wei, F. 2021	4.45	3.1000	12	4.69	2.4800	13	18.9%	−0.08 [−0.87, 0.70]
Nesrin O. Ercelen 2020	15.19	9.4900	8	9.90	4.3900	3	6.3%	0.56 [−0.80, 1.92]
**Total (95% CI)**			**79**			**60**	**100.0%**	**−0.16 [−0.50, 0.19]**

Heterogeneity: $Tau^2 = 0$; $Chi^2 = 2.12$, df = 3 (P = 0.55); $I^2 = 0\%$
Test for overall effect: Z = −0.90 (P = 0.37)

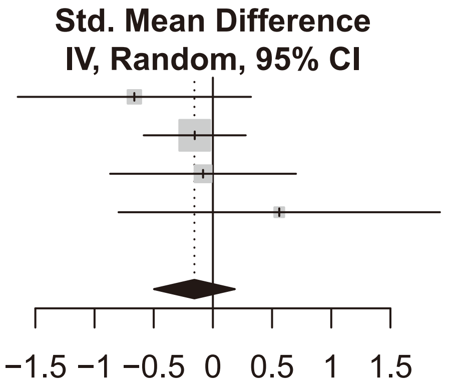

b. Forest plot of neutrophils (day5-8): Std. Mean Difference (95% CI) and pooled estimates.

Study	Experimental Mean	Experimental SD	Experimental Total	Control Mean	Control SD	Control Total	Weight	Std. Mean Difference IV, Random, 95% CI
Lanzoni, G. 2021	1.38	1.0700	8	0.80	0.4100	9	12.0%	0.70 [−0.29, 1.69]
Ventura−Carmenate, Y. 2021	2.10	2.9500	51	1.64	2.7200	35	63.2%	0.16 [−0.27, 0.59]
Wei, F. 2021	1.57	0.6000	12	1.35	0.9600	13	18.9%	0.26 [−0.53, 1.05]
Nesrin O. Ercelen 2020	1.32	0.8300	8	0.60	0.2200	3	6.0%	0.89 [−0.51, 2.29]
**Total (95% CI)**			**79**			**60**	**100.0%**	**0.29 [−0.06, 0.63]**

Heterogeneity: $Tau^2 = 0$; $Chi^2 = 1.71$, df = 3 (P = 0.63); $I^2 = 0\%$
Test for overall effect: Z = 1.64 (P = 0.10)

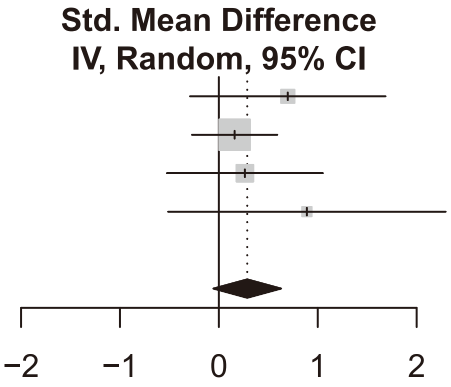

c. Forest plot of lymphocytes (day5-8): Std. Mean Difference (95% CI) and pooled estimates.

Study	Experimental Mean	Experimental SD	Experimental Total	Control Mean	Control SD	Control Total	Weight	Std. Mean Difference IV, Random, 95% CI
Meng, F. 2020	239.17	65.5400	9	244.28	75.9200	9	67.5%	−0.07 [−0.99, 0.86]
Nesrin O. Ercelen 2020	260.69	83.6600	8	242.33	62.9700	3	32.5%	0.21 [−1.12, 1.54]
**Total (95% CI)**			**17**			**12**	**100.0%**	**0.02 [−0.74, 0.78]**

Heterogeneity: $Tau^2 = 0$; $Chi^2 = 0.11$, df = 1 (P = 0.74); $I^2 = 0\%$

Test for overall effect: Z = 0.06 (P = 0.95)

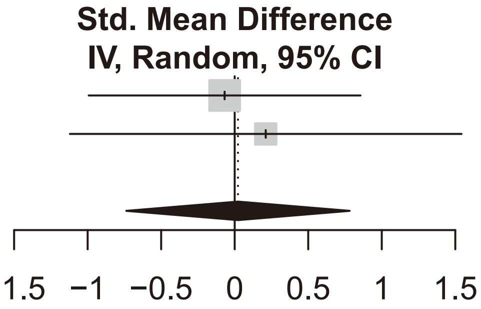

d. Forest plot of PLT (day0-4): Std. Mean Difference (95% CI) and pooled estimates.

Study	Experimental Mean	Experimental SD	Experimental Total	Control Mean	Control SD	Control Total	Weight	Std. Mean Difference IV, Random, 95% CI
Lanzoni, G. 2021	342.00	136.3300	9	397.89	135.5900	9	25.6%	−0.39 [−1.33, 0.54]
Meng, F. 2020	233.00	71.4800	9	224.56	38.5400	9	26.1%	0.14 [−0.79, 1.07]
Wei, F. 2021	182.80	68.0000	12	212.20	82.4000	13	35.6%	−0.37 [−1.17, 0.42]
Nesrin O. Ercelen 2020	266.00	115.4500	8	240.67	61.0300	3	12.6%	0.22 [−1.11, 1.55]
**Total (95% CI)**			**38**			**34**	**100.0%**	**−0.17 [−0.64, 0.30]**

Heterogeneity: $Tau^2 = 0$; $Chi^2 = 1.23$, df = 3 (P = 0.75); $I^2 = 0\%$
Test for overall effect: Z = −0.70 (P = 0.48)

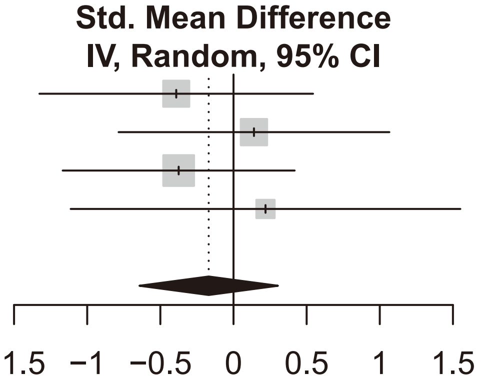

e. Forest plot of PLT (day5-8): Std. Mean Difference (95% CI) and pooled estimates

Study	Experimental Mean	Experimental SD	Experimental Total	Control Mean	Control SD	Control Total	Weight	Std. Mean Difference IV, Random, 95% CI
Meng, F. 2020	9.08	10.4700	9	7.00	9.6900	9	39.4%	0.20 [−0.73, 1.12]
Adas, G. 2021	103.45	92.4100	10	165.00	105.1900	10	41.8%	−0.60 [−1.50, 0.30]
Nesrin O. Ercelen 2020	147.50	113.3600	8	190.17	40.6200	3	18.8%	−0.38 [−1.72, 0.96]
**Total (95% CI)**			**27**			**22**	**100.0%**	**−0.24 [−0.83, 0.34]**

Heterogeneity: $Tau^2 = 0$; $Chi^2 = 1.49$, df = 2 (P = 0.47); $I^2 = 0\%$

Test for overall effect: Z = −0.82 (P = 0.41)

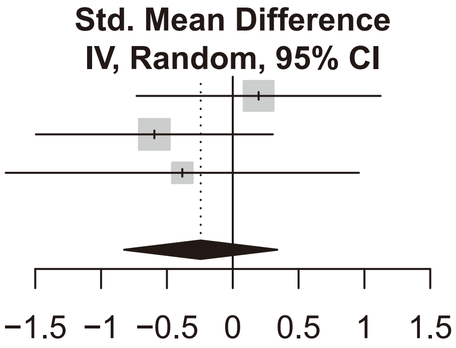

f. Forest plot of CRP (day0-4): Std. Mean Difference (95% CI) and pooled estimates.

Study	Experimental Mean	Experimental SD	Experimental Total	Control Mean	Control SD	Control Total	Weight	Std. Mean Difference IV, Random, 95% CI
Lanzoni, G. 2021	101.01	131.0500	8	112.55	104.7000	11	14.5%	−0.09 [−1.01, 0.82]
Meng, F. 2020	4.86	9.5800	9	2.57	2.2800	9	13.9%	0.31 [−0.62, 1.24]
Ventura−Carmenate, Y. 2021	3.60	101.4800	31	16.45	320.3500	24	33.4%	−0.06 [−0.59, 0.48]
Wei, F. 2021	3.09	4.0700	12	21.43	44.0200	13	17.9%	−0.56 [−1.36, 0.25]
Adas, G. 2021	99.20	90.8000	10	139.60	72.0500	10	15.0%	−0.47 [−1.36, 0.42]
Nesrin O. Ercelen 2020	77.18	78.5000	8	210.33	45.7300	3	5.3%	−1.68 [−3.26, −0.10]
**Total (95% CI)**			**78**			**70**	**100.0%**	**−0.25 [−0.62, 0.13]**

Heterogeneity: $Tau^2 = 0.035$; $Chi^2 = 5.93$, df = 5 (P = 0.31); $I^2 = 16\%$
Test for overall effect: Z = −1.30 (P = 0.19)

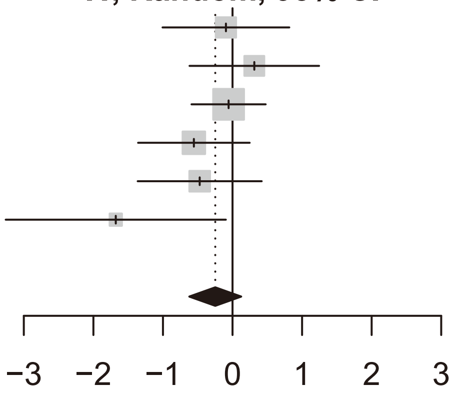

g. Forest plot of CRP (day5-8): Std. Mean Difference (95% CI) and pooled estimates.

Study	Experimental Mean	Experimental SD	Experimental Total	Control Mean	Control SD	Control Total	Weight	Std. Mean Difference IV, Random, 95% CI
Wei, F. 2021	8.63	13.8900	12	18.84	35.0000	13	55.6%	−0.37 [−1.16, 0.43]
Adas, G. 2021	91.30	82.1000	10	117.30	44.2000	10	44.4%	−0.38 [−1.26, 0.51]
**Total (95% CI)**			**22**			**23**	**100.0%**	**−0.37 [−0.96, 0.22]**

Heterogeneity: $Tau^2 = 0$; $Chi^2 = 0.00$, df = 1 (P = 0.98); $I^2 = 0\%$

Test for overall effect: Z = −1.23 (P = 0.22)

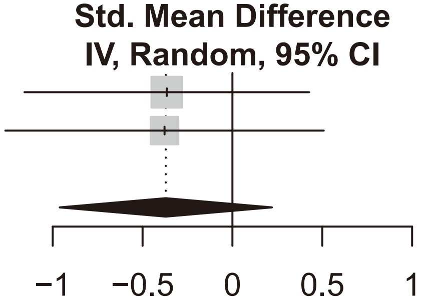

h. Forest plot of IL-6 (day5-8): Std. Mean Difference (95% CI) and pooled estimates.

Study	Experimental Mean	Experimental SD	Experimental Total	Control Mean	Control SD	Control Total	Weight	Std. Mean Difference IV, Random, 95% CI
Lanzoni, G. 2021	325.83	151.8400	11	890.80	1155.3000	11	49.0%	−0.66 [−1.52, 0.20]
Wei, F. 2021	11.20	2.1800	12	9.40	4.1200	13	51.0%	0.52 [−0.28, 1.32]
**Total (95% CI)**			**23**			**24**	**100.0%**	**−0.06 [−1.21, 1.10]**

Heterogeneity: $Tau^2 = 0.518$; $Chi^2 = 3.87$, df = 1 (P = 0.05); $I^2 = 74\%$
Test for overall effect: Z = −0.10 (P = 0.92)

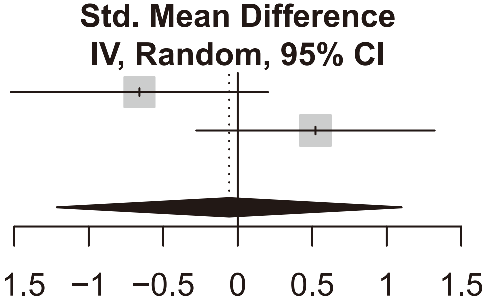

I. Forest plot of TNF-α (day5-8): Std. Mean Difference (95% CI) and pooled estimates.

Study	Experimental Mean	Experimental SD	Experimental Total	Control Mean	Control SD	Control Total	Weight	Std. Mean Difference IV, Random, 95% CI
Meng, F. 2020	0.43	0.3100	9	0.63	0.5900	9	37.4%	−0.40 [−1.34, 0.53]
Adas, G. 2021	2.55	1.5500	10	1.70	1.0600	10	38.7%	0.61 [−0.29, 1.51]
Nesrin O. Ercelen 2020	3.73	3.8200	8	7.34	4.1200	3	23.9%	−0.85 [−2.25, 0.55]
**Total (95% CI)**			**27**			**22**	**100.0%**	**−0.12 [−0.96, 0.73]**

Heterogeneity: $Tau^2 = 0.270$; $Chi^2 = 3.88$, df = 2 (P = 0.14); $I^2 = 48\%$
Test for overall effect: Z = −0.27 (P = 0.79)

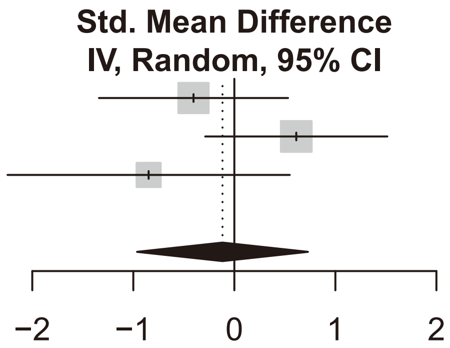

j. Forest plot of D-dimer (day0-4): Std. Mean Difference (95% CI) and pooled estimates.

Study	Experimental Mean	Experimental SD	Experimental Total	Control Mean	Control SD	Control Total	Weight	Std. Mean Difference IV, Random, 95% CI
Lanzoni, G. 2021	6.20	11.2900	9	4.69	3.3800	10	13.5%	0.18 [−0.73, 1.08]
Meng, F. 2020	0.63	0.4000	9	1.38	1.8200	9	12.3%	−0.54 [−1.49, 0.40]
Ventura−Carmenate, Y. 2021	0.20	3.9400	49	1.51	588.1600	31	54.5%	−0.00 [−0.45, 0.45]
Adas, G. 2021	3.20	2.7000	10	1.90	1.1000	10	13.6%	0.60 [−0.30, 1.50]
Nesrin O. Ercelen 2020	7.10	4.5500	8	4.61	2.9700	3	6.0%	0.54 [−0.82, 1.89]
**Total (95% CI)**			**85**			**63**	**100.0%**	**0.07 [−0.26, 0.40]**

Heterogeneity: $Tau^2 = 0$; $Chi^2 = 3.57$, df = 4 (P = 0.47); $I^2 = 0\%$
Test for overall effect: Z = 0.41 (P = 0.68)

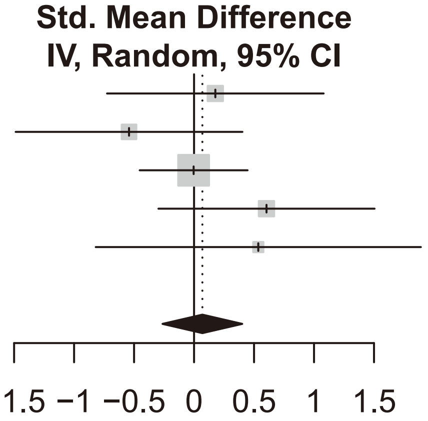

k. Forest plot of D-dimer (day5-8): Std. Mean Difference (95% CI) and pooled estimates.

Study	Experimental Mean	Experimental SD	Experimental Total	Control Mean	Control SD	Control Total	Weight	Std. Mean Difference IV, Random, 95% CI
Adas, G. 2021	451.40	119.3200	10	617.05	137.0300	10	66.1%	−1.23 [−2.21, −0.26]
Nesrin O. Ercelen 2020	591.81	215.4500	8	716.33	73.9900	3	33.9%	−0.59 [−1.95, 0.77]
**Total (95% CI)**			**18**			**13**	**100.0%**	**−1.02 [−1.81, −0.22]**

Heterogeneity: $Tau^2 = 0$; $Chi^2 = 0.57$, df = 1 (P = 0.45); $I^2 = 0\%$

Test for overall effect: Z = −2.51 (P = 0.01)

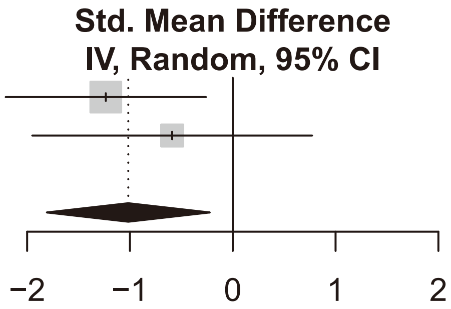

l. Forest plot of fibrinogen (day0-4): Std. Mean Difference (95% CI) and pooled estimates.

Study	Experimental Mean	Experimental SD	Experimental Total	Control Mean	Control SD	Control Total	Weight	Std. Mean Difference IV, Random, 95% CI	Std. Mean Difference IV, Random, 95% CI
Ventura−Carmenate, Y. 2021	350.00	352.4200	31	470.00	442.3900	13	46.7%	−0.31 [−0.96, 0.34]	
Adas, G. 2021	498.40	146.1000	10	561.30	144.1000	10	37.1%	−0.42 [−1.30, 0.47]	
Nesrin O. Ercelen 2020	451.50	211.2100	8	940.33	158.6300	3	16.3%	−2.23 [−3.98, −0.47]	
**Total (95% CI)**			**49**			**26**	**100.0%**	**−0.66 [−1.47, 0.15]**	

Heterogeneity: $Tau^2 = 0.258$; $Chi^2 = 4.09$, df = 2 (P = 0.13); $I^2 = 51\%$
Test for overall effect: Z = −1.59 (P = 0.11)

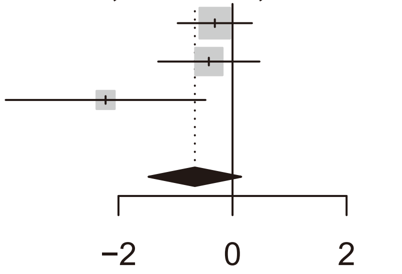

m. Forest plot of fibrinogen (day5-8): Std. Mean Difference (95% CI) and pooled estimates.

	Experimental			Control				Std. Mean Difference
**Study**	**Mean**	**SD**	**Total**	**Mean**	**SD**	**Total**	**Weight**	**IV, Random, 95% CI**
Meng, F. 2020	459.72	348.7000	9	502.00	302.6300	9	38.8%	−0.12 [−1.05, 0.80]
Adas, G. 2021	648.10	806.3700	10	812.60	729.8400	10	43.0%	−0.20 [−1.08, 0.67]
Nesrin O. Ercelen 2020	1059.04	1124.8900	8	507.33	232.1500	3	18.2%	0.51 [−0.85, 1.86]
**Total (95% CI)**			**27**			**22**	**100.0%**	**−0.04 [−0.62, 0.53]**

Heterogeneity: $Tau^2 = 0$; $Chi^2 = 0.79$, df = 2 (P = 0.67); $I^2 = 0\%$
Test for overall effect: Z = −0.15 (P = 0.88)

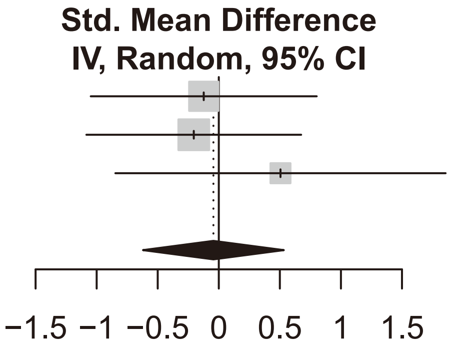

n. Forest plot of ferritin (day0-4): Std. Mean Difference (95% CI) and pooled estimates.

Study	Experimental Mean	Experimental SD	Experimental Total	Control Mean	Control SD	Control Total	Weight	Std. Mean Difference IV, Random, 95% CI
Meng, F. 2020	502.44	280.6000	9	571.92	508.0100	9	40.4%	−0.16 [−1.09, 0.76]
Adas, G. 2021	379.10	175.3000	10	1088.50	1082.4000	10	40.4%	−0.88 [−1.80, 0.05]
O.Ercelen, N.2020 2020	546.62	266.7100	8	724.33	670.1100	3	19.2%	−0.41 [−1.76, 0.93]
**Total (95% CI)**			**27**			**22**	**100.0%**	**−0.50 [−1.09, 0.09]**

Heterogeneity: $Tau^2 = 0$; $Chi^2 = 1.16$, df = 2 ($P = 0.56$); $I^2 = 0\%$
Test for overall effect: $Z = -1.66$ ($P = 0.10$)

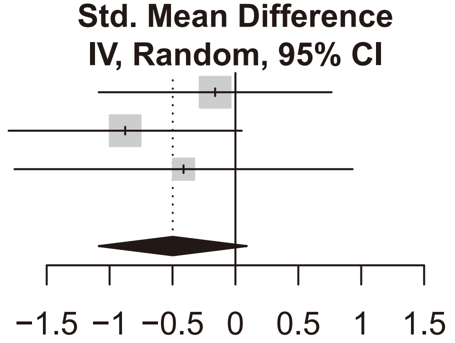

o. Forest plot of ferritin (day5-8): Std. Mean Difference (95% CI) and pooled estimates.
