# Supplementary material for: Safety and efficiency of stem cell therapy for COVID-19: a systematic review and meta-analysis
Source: Glob Health Res Policy. 2022 Jun 23;7:19. doi: 10.1186/s41256-022-00251-5 (PMC9217728; doi:10.1186/s41256-022-00251-5)
Supplement: Supplementary file 9 — Additional file 9. Results of subgroup analysis of outcomes. Results of Meta-analysis of AEs, mortality, hospital stay, neutrophils, lymphocytes, platelets, CRP and D-dimer according to the type of studies. a. Subgroup analysis of AEs b. Subgroup analysis of mortality c. Subgroup analysis of length of hospital stay d. Subgroup analysis of neutrophils (day5–8) e. Subgroup analysis of lymphocytes (day5–8) f. Subgroup analysis of PLT (day5–8) g. Subgroup analysis of CRP (day5–8) h. Subgroup analysis of D-dimer (day5–8). [file 41256_2022_251_MOESM9_ESM.pdf]

Study or Subgroup	Experimental Events	Experimental Total	Control Events	Control Total	Odds Ratio GLMM, Fixed + Random, 95% CI
**Type = RCT**					
Lanzoni, G. 2020	8	12	11	12	0.18 [0.02, 1.95]
Ventura−Carmenate, Y. 2020	50	69	51	70	0.98 [0.46, 2.07]
Shi, L. 2022	54	65	26	35	1.70 [0.63, 4.61]
Zhu, R. 2021	3	29	13	29	0.14 [0.03, 0.58]
**Total (fixed effect, 95% CI)**		**175**		**146**	**0.72 [0.43, 1.22]**
**Total (random effects, 95% CI)**					**0.57 [0.21, 1.60]**
Heterogeneity: $Tau^2 = 0.636$; $Chi^2 = 9.82$, df = 3 (P = 0.02); $I^2 = 69\%$					
Test for overall effect (fixed effect): Z = −1.22 (P = 0.22)					
Test for overall effect (random effects): Z = −1.06 (P = 0.29)					
**Type = NRCT**					
Xu, X. 2021	20	26	18	18	0.09 [0.00, 1.62]
Test for overall effect (fixed effect): Z = NA (P = NA)					
Test for overall effect (random effects): Z = NA (P = NA)					
**Total (fixed effect, 95% CI)**		**201**		**164**	**0.63 [0.38, 1.05]**
**Total (random effects, 95% CI)**					**0.39 [0.12, 1.33]**

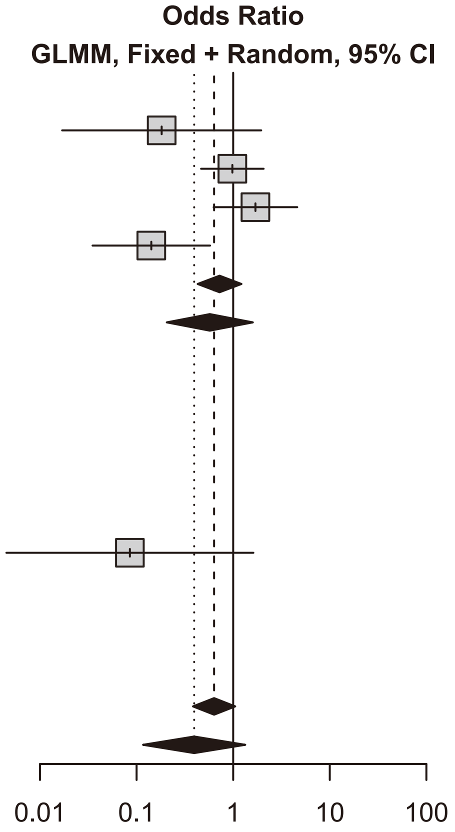

Heterogeneity: $Tau^2 = 1.061$; $Chi^2 = 9.63$, df = 4 (P = 0.05); $I^2 = 58\%$
Test for overall effect (fixed effect): Z = −1.77 (P = 0.08)
Test for overall effect (random effects): Z = −1.50 (P = 0.13)
Test for subgroup differences (fixed effect): $Chi^2 = 0.00$, df = 1 (P = 1.00)
Test for subgroup differences (random effects): $Chi^2 = 0.00$, df = 1 (P = 1.00)

a. Subgroup analysis of AEs

Study or Subgroup	Experimental Events	Experimental Total	Control Events	Control Total	Odds Ratio GLMM, Random, 95% CI
**Type = RCT**					
Lanzoni, G. 2021	1	11	7	12	0.07 [0.01, 0.75]
Ventura−Carmenate, Y. 2021	4	69	7	70	0.55 [0.15, 1.98]
Shi, L. 2021	0	65	0	35	0.54 [0.01, 27.90]
Zhu, R. 2021	0	29	2	29	0.19 [0.01, 4.06]
Adas, G. 2021	3	10	6	10	0.29 [0.04, 1.82]
Dilogo, I. H. 2021	10	20	16	20	0.25 [0.06, 1.02]
**Total (95% CI)**		**204**		**176**	**0.26 [0.12, 0.57]**
Heterogeneity: $Tau^2 = 0$; $Chi^2 = 2.38$, df = 5 (P = 0.79); $I^2 = 0\%$					
Test for overall effect: Z = −3.37 (P < 0.01)					
**Type = NRCT**					
Leng, Z. 2020	0	7	1	3	0.11 [0.00, 3.70]
Meng, F. 2020	0	9	0	9	1.00 [0.02, 55.80]
Shu, L. 2020	0	12	3	29	0.30 [0.01, 6.32]
Häberle, H. 2021	1	5	10	18	0.20 [0.02, 2.16]
Xu, X. 2021	2	26	6	18	0.17 [0.03, 0.95]
O.Ercelen, N. 2020	4	8	2	3	0.50 [0.03, 7.99]
**Total (95% CI)**		**67**		**80**	**0.17 [0.05, 0.57]**
Heterogeneity: $Tau^2 = 0$; $Chi^2 = 0.44$, df = 5 (P = 0.99); $I^2 = 0\%$					
Test for overall effect: Z = −2.88 (P < 0.01)					
**Total (95% CI)**		**271**		**256**	**0.24 [0.13, 0.45]**
Heterogeneity: $Tau^2 < 0.001$; $Chi^2 = 2.83$, df = 9 (P = 0.97); $I^2 = 0\%$					
Test for overall effect: Z = −4.37 (P < 0.01)					
Test for subgroup differences: $Chi^2 = 0.33$, df = 1 (P = 0.56)					

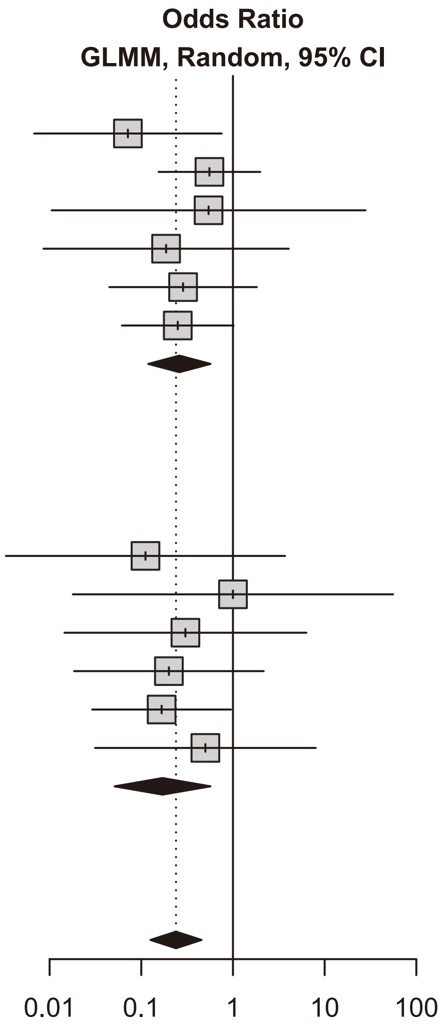

b. Subgroup analysis of mortality

Study or Subgroup	Experimental Mean	SD	Total	Control Mean	SD	Total	Weight	Std. Mean Difference IV, Random, 95% CI
**Type = NRCT**								
Meng, F. 2020	20.74	6.12	9	23.37	6.12	9	13.72%	−0.41 [−1.35, 0.53]
Test for overall effect: Z = −0.86 (P = 0.39)								
**Type = RCT**								
Torres Zambrano, Gina M. 2021	7.43	8.88	69	14.16	25.95	70	42.56%	−0.34 [−0.68, −0.01]
Zhu, R. 2021	11.00	4.68	29	15.00	6.24	29	28.95%	−0.72 [−1.25, −0.18]
Adas, G. 2021	55.52	26.19	10	45.62	10.02	10	14.78%	0.48 [−0.41, 1.37]
**Total (95% CI)**			**108**			**109**	**86.28%**	**−0.30 [−0.81, 0.20]**
Heterogeneity: $Tau^2 = 0.118$; $Chi^2 = 5.12$, df = 2 (P = 0.08); $I^2 = 61\%$								
Test for overall effect: Z = −1.18 (P = 0.24)								
**Total (95% CI)**			**117**			**118**	**100.00%**	**−0.34 [−0.73, 0.05]**
Heterogeneity: $Tau^2 = 0.065$; $Chi^2 = 5.12$, df = 3 (P = 0.16); $I^2 = 41\%$								
Test for overall effect: Z = −1.69 (P = 0.09)								
Test for subgroup differences: $Chi^2 = 0.04$, df = 1 (P = 0.85)								

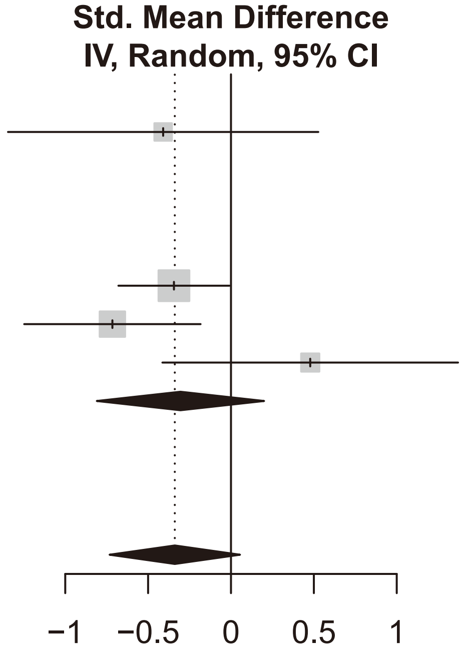

c. Subgroup analysis of length of hospital stay

Study or Subgroup	Experimental Mean	Experimental SD	Experimental Total	Control Mean	Control SD	Control Total	Weight	Std. Mean Difference IV, Random, 95% CI
Type = NRCT								
Lanzoni, G. 2021	9.74	4.2700	8	13.40	5.9500	9	12.0%	−0.66 [−1.65, 0.32]
Ventura−Carmenate, Y. 2021	4.30	15.6700	51	7.27	23.4100	35	62.8%	−0.15 [−0.58, 0.28]
Total (95% CI)			59			44	74.8%	−0.24 [−0.63, 0.16]
Heterogeneity: $Tau^2 = 0; Chi^2 = 0.86, df = 1 (P = 0.35); I^2 = 0\%$								
Test for overall effect: Z = −1.17 (P = 0.24)								
Type = RCT								
Wei, F. 2021	4.45	3.1000	12	4.69	2.4800	13	18.9%	−0.08 [−0.87, 0.70]
O.Ercelen, N.2020 2020	15.19	9.4900	8	9.90	4.3900	3	6.3%	0.56 [−0.80, 1.92]
Total (95% CI)			20			16	25.2%	0.08 [−0.60, 0.76]
Heterogeneity: $Tau^2 = 0; Chi^2 = 0.65, df = 1 (P = 0.42); I^2 = 0\%$								
Test for overall effect: Z = 0.23 (P = 0.82)								
**Total (95% CI)**			**79**			**60**	**100.0%**	**−0.16 [−0.50, 0.19]**
Heterogeneity: $Tau^2 = 0; Chi^2 = 2.12, df = 3 (P = 0.55); I^2 = 0\%$								
Test for overall effect: Z = −0.90 (P = 0.37)								
Test for subgroup differences: $Chi^2 = 0.61, df = 1 (P = 0.43)$								

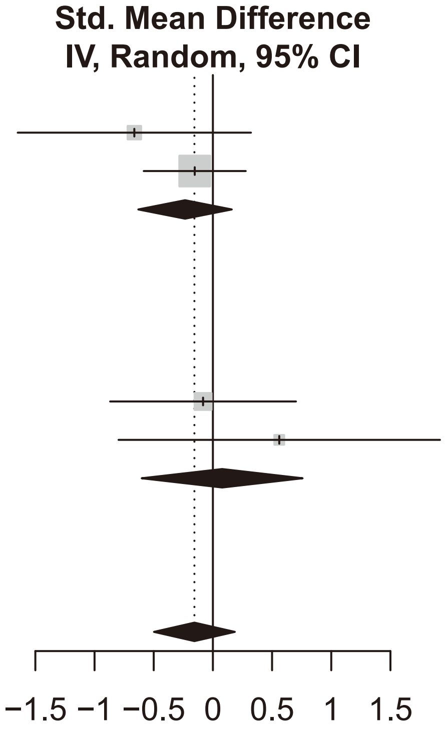

d. Subgroup analysis of neutrophils (day5-8)

Study or Subgroup	Experimental Mean	Experimental SD	Experimental Total	Control Mean	Control SD	Control Total	Weight	Std. Mean Difference IV, Random, 95% CI
**Type = RCT**								
Lanzoni, G. 2021	1.38	1.0700	8	0.80	0.4100	9	12.0%	0.70 [−0.29, 1.69]
Ventura−Carmenate, Y. 2021	2.10	2.9500	51	1.64	2.7200	35	63.2%	0.16 [−0.27, 0.59]
**Total (95% CI)**			**59**			**44**	**75.2%**	**0.25 [−0.15, 0.64]**
Heterogeneity: $Tau^2 = 0$; $Chi^2 = 0.95$, df = 1 (P = 0.33); $I^2 = 0\%$								
Test for overall effect: Z = 1.22 (P = 0.22)								
**Type = NRCT**								
Wei, F. 2021	1.57	0.6000	12	1.35	0.9600	13	18.9%	0.26 [−0.53, 1.05]
O.Ercelen, N.2020 2020	1.32	0.8300	8	0.60	0.2200	3	6.0%	0.89 [−0.51, 2.29]
**Total (95% CI)**			**20**			**16**	**24.8%**	**0.41 [−0.27, 1.10]**
Heterogeneity: $Tau^2 = 0$; $Chi^2 = 0.58$, df = 1 (P = 0.45); $I^2 = 0\%$								
Test for overall effect: Z = 1.18 (P = 0.24)								
**Total (95% CI)**			**79**			**60**	**100.0%**	**0.29 [−0.06, 0.63]**
Heterogeneity: $Tau^2 = 0$; $Chi^2 = 1.71$, df = 3 (P = 0.63); $I^2 = 0\%$								
Test for overall effect: Z = 1.64 (P = 0.10)								
Test for subgroup differences: $Chi^2 = 0.17$, df = 1 (P = 0.68)								

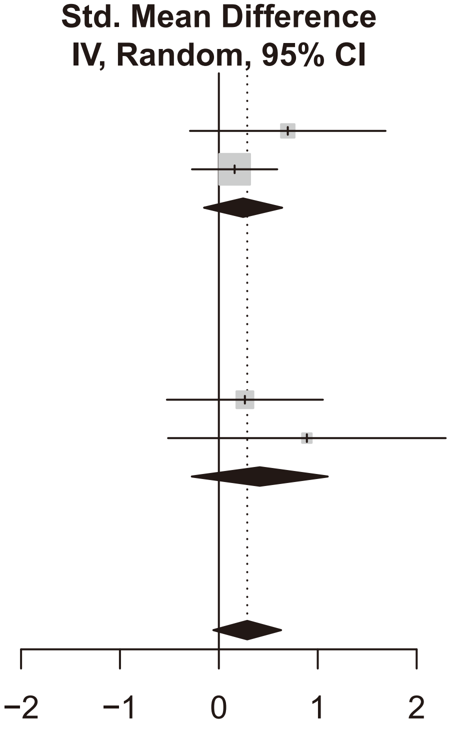

e. Subgroup analysis of lymphocytes (day5-8)

Study or Subgroup	Experimental Mean	Experimental SD	Experimental Total	Control Mean	Control SD	Control Total	Weight	Std. Mean Difference IV, Random, 95% CI
Type = RCT								
Lanzoni, G. 2021	342.00	136.3300	9	397.89	135.5900	9	25.6%	−0.39 [−1.33, 0.54]
Test for overall effect: Z = −0.82 (P = 0.41)								
Type = NRCT								
Meng, F. 2020	233.00	71.4800	9	224.56	38.5400	9	26.1%	0.14 [−0.79, 1.07]
Wei, F. 2021	182.80	68.0000	12	212.20	82.4000	13	35.6%	−0.37 [−1.17, 0.42]
O.Ercelen, N.2020 2020	266.00	115.4500	8	240.67	61.0300	3	12.6%	0.22 [−1.11, 1.55]
Total (95% CI)			29			25	74.4%	−0.09 [−0.64, 0.46]
Heterogeneity: $Tau^2 = 0$; $Chi^2 = 0.94$, df = 2 (P = 0.63); $I^2 = 0\%$								
Test for overall effect: Z = −0.33 (P = 0.74)								
**Total (95% CI)**			**38**			**34**	**100.0%**	**−0.17 [−0.64, 0.30]**
Heterogeneity: $Tau^2 = 0$; $Chi^2 = 1.23$, df = 3 (P = 0.75); $I^2 = 0\%$								
Test for overall effect: Z = −0.70 (P = 0.48)								
Test for subgroup differences: $Chi^2 = 0.29$, df = 1 (P = 0.59)								

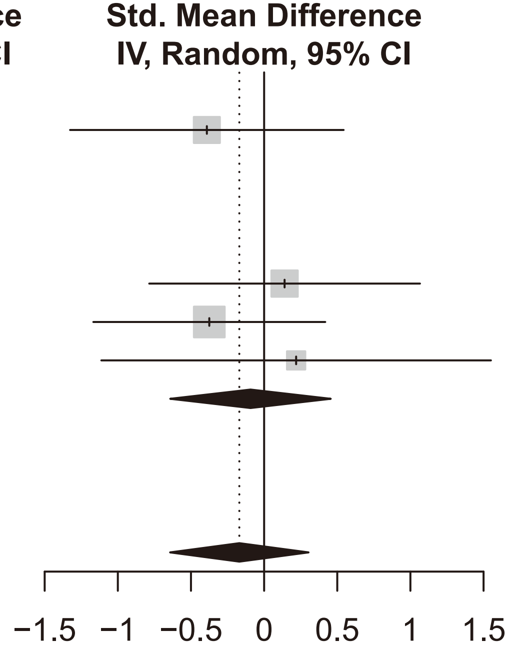

f. Subgroup analysis of PLT (day5-8)

Study or Subgroup	Experimental Mean	Experimental SD	Experimental Total	Control Mean	Control SD	Control Total	Weight	Std. Mean Difference IV, Random, 95% CI
Type = RCT								
Lanzoni, G. 2021	101.01	131.0500	8	112.55	104.7000	11	14.5%	−0.09 [−1.01, 0.82]
Ventura−Carmenate, Y. 2021	3.60	101.4800	31	16.45	320.3500	24	33.4%	−0.06 [−0.59, 0.48]
Adas, G. 2021	99.20	90.8000	10	139.60	72.0500	10	15.0%	−0.47 [−1.36, 0.42]
**Total (95% CI)**			**49**			**45**	**62.8%**	**−0.15 [−0.56, 0.26]**
Heterogeneity: $Tau^2 = 0$; $Chi^2 = 0.63$, df = 2 (P = 0.73); $I^2 = 0\%$								
Test for overall effect: Z = −0.73 (P = 0.47)								
Type = NRCT								
Meng, F. 2020	4.86	9.5800	9	2.57	2.2800	9	13.9%	0.31 [−0.62, 1.24]
Wei, F. 2021	3.09	4.0700	12	21.43	44.0200	13	17.9%	−0.56 [−1.36, 0.25]
O.Ercelen, N.2020 2020	77.18	78.5000	8	210.33	45.7300	3	5.3%	−1.68 [−3.26, −0.10]
**Total (95% CI)**			**29**			**25**	**37.2%**	**−0.48 [−1.43, 0.47]**
Heterogeneity: $Tau^2 = 0.408$; $Chi^2 = 4.9$, df = 2 (P = 0.09); $I^2 = 59\%$								
Test for overall effect: Z = −1.00 (P = 0.32)								
**Total (95% CI)**			**78**			**70**	**100.0%**	**−0.25 [−0.62, 0.13]**

Heterogeneity: $Tau^2 = 0.035$; $Chi^2 = 5.93$, df = 5 (P = 0.31); $I^2 = 16\%$

Test for overall effect: Z = −1.30 (P = 0.19)

Test for subgroup differences: $Chi^2 = 0.39$, df = 1 (P = 0.53)

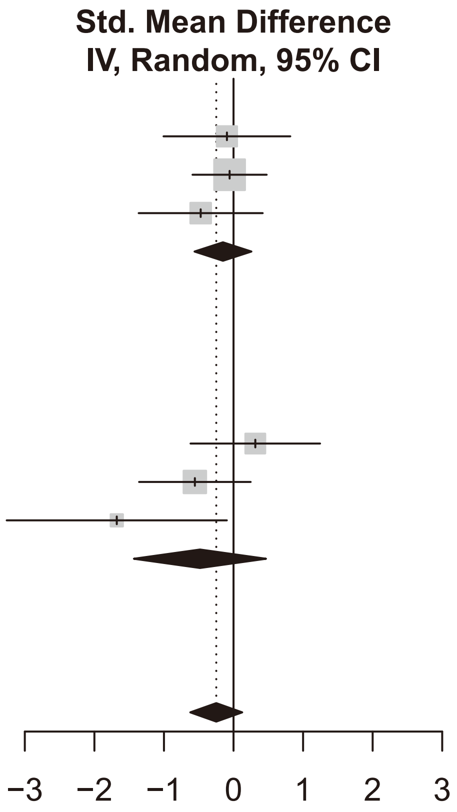

g. Subgroup analysis of CRP (day5-8)

Study or Subgroup	Experimental Mean	SD	Total	Control Mean	SD	Total	Weight	Std. Mean Difference IV, Random, 95% CI
Type = RCT								
Lanzoni, G. 2021	6.20	11.2900	9	4.69	3.3800	10	13.5%	0.18 [−0.73, 1.08]
Ventura−Carmenate, Y. 2021	0.20	3.9400	49	1.51	588.1600	31	54.5%	−0.00 [−0.45, 0.45]
Adas, G. 2021	3.20	2.7000	10	1.90	1.1000	10	13.6%	0.60 [−0.30, 1.50]
**Total (95% CI)**			**68**			**51**	**81.6%**	**0.13 [−0.24, 0.50]**
Heterogeneity: $Tau^2 = 0$; $Chi^2 = 1.41$, df = 2 (P = 0.49); $I^2 = 0\%$								
Test for overall effect: Z = 0.68 (P = 0.50)								
Type = NRCT								
Meng, F. 2020	0.63	0.4000	9	1.38	1.8200	9	12.3%	−0.54 [−1.49, 0.40]
O.Ercelen, N.2020 2020	7.10	4.5500	8	4.61	2.9700	3	6.0%	0.54 [−0.82, 1.89]
**Total (95% CI)**			**17**			**12**	**18.4%**	**−0.12 [−1.15, 0.91]**
Heterogeneity: $Tau^2 = 0.225$; $Chi^2 = 1.63$, df = 1 (P = 0.20); $I^2 = 39\%$								
Test for overall effect: Z = −0.22 (P = 0.82)								
**Total (95% CI)**			**85**			**63**	**100.0%**	**0.07 [−0.26, 0.40]**

Heterogeneity: $Tau^2 = 0$; $Chi^2 = 3.57$, df = 4 (P = 0.47); $I^2 = 0\%$

Test for overall effect: Z = 0.41 (P = 0.68)

Test for subgroup differences: $Chi^2 = 0.19$, df = 1 (P = 0.66)

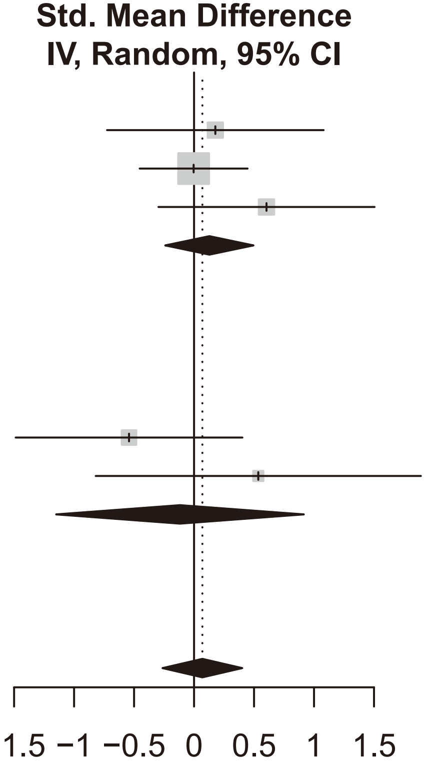

h. Subgroup analysis of D-dimer (day5-8)
